# Bimodal Fluorescent Conjugate Based on Prostate-Specific Membrane Antigen Ligands with the Chelating Agent DOTA and SulfoCy5 Dye: Synthesis, Radiolabeling, and Biological Activity

**DOI:** 10.3390/ijms27083502

**Published:** 2026-04-14

**Authors:** Aleksei E. Machulkin, Stanislav A. Petrov, Nina S. Butakova, Aleksandr S. Lunev, Kristina A. Petrosova, Radik R. Shafikov, Dmitry A. Skvortsov, Iurii A. Mitrofanov, Mariia N. Ivashkovaskaia, Elena K. Beloglazkina, Anton A. Larenkov

**Affiliations:** 1 Chemistry Department, Lomonosov Moscow State University, Leninskie Gory, Building 1/3, GSP-1, 119991 Moscow, Russia; 2 Department of Biochemistry, People’s Friendship University of Russia Named After Patrice Lumumba (RUDN University), 117198 Moscow, Russia; 3 FSBI State Research Center—Burnasyan Federal Medical Biophysical Center of Federal Medical Biological Agency, 123098 Moscow, Russia; 4 Shemyakin and Ovchinnikov Institute of Bioorganic Chemistry Russian Academy of Sciences, 117997 Moscow, Russia

**Keywords:** PSMA, prostate cancer, drug delivery, bimodal conjugates, DOTA-conjugates

## Abstract

Prostate-specific membrane antigen (PSMA) is an essential zinc-dependent metalloprotease classified within the type II transmembrane protein family, often referred to as glutamate carboxypeptidase II (GCPII). PSMA is recognized as a particularly promising target for both the diagnosis and therapeutic intervention of prostate cancer. In this study, we designed and synthesized PSMA-targeted DOTA-loaded bimodal conjugate **11** with SulfoCy5 fluorescent dye, performed in vitro characterization, and analyzed biodistribution in vivo. At 40–100 nM concentrations, the resulting conjugate demonstrated reliable visualization of tumor cells, on par with the reference PSMA-SylfoCy5 compound. In vivo biodistribution analysis of [^68^Ga]Ga-**11** in mice demonstrated a reduction in renal accumulation in comparison with dye-free conjugate [^68^Ga]Ga-**10**. The specificity of [^68^Ga]Ga-**11** for PSMA was confirmed in a murine LNCaP xenograft model: its effective accumulation in tumors and kidneys, as well as relatively rapid elimination from non-target tissues, make it a promising agent for PET imaging but not radionuclide therapy.

## 1. Introduction

Prostate cancer (PCa) is a multifaceted and biologically diverse disease, recognized as one of the most frequently diagnosed cancers among men globally, which continues to be a leading cause of cancer-related mortality in this population [1,2]. While prostate cancer is characterized as one of the slower-growing malignant tumors, its potential to become lethal increases significantly upon metastasis. Thus, the early detection of this disease is critical for the implementation of effective treatment strategies. Various functional imaging modalities utilized for the detection of prostate cancer include transrectal ultrasound, magnetic resonance imaging (MRI), computed tomography (CT), single photon emission computed tomography (SPECT), and positron emission tomography (PET). Nonetheless, the specificity of current imaging techniques employed in the assessment of PCa remains constrained [3,4].

The selection of treatment modalities for prostate cancer is dependent upon the disease’s progression stage. In cases of localized prostate cancer, therapeutic options range from radical prostatectomy to external beam radiation therapy. In the context of advanced or metastatic prostate cancer, androgen deprivation therapy (ADT) and chemotherapy are standard approaches [5]. Furthermore, therapeutic radiopharmaceuticals (RPs) may be employed, although only ^177^Lu-PSMA-617-based radiopharmaceuticals have demonstrated significant efficacy in treating prostate cancer [6,7]. Thus, the advancement of novel diagnostic and therapeutic agents targeting prostate cancer remains a critical area of ongoing research.

Prostate-specific membrane antigen (PSMA) demonstrates a markedly high presence on the cell membrane of prostate cancer cells, rendering it an appealing molecular target for precise delivery to the prostate [8,9]. Research indicates that this transmembrane receptor is overexpressed by as much as 1000-fold in prostate tumor cells in comparison to normal prostate tissue, with its expression levels exhibiting a direct correlation with both disease status and stage of progression [10,11]. Furthermore, aside from its prominent expression in prostate cancer cells, PSMA has also been implicated in the angiogenesis associated with several other solid tumors [12,13,14,15,16,17,18,19,20]. Due to the enrichment of prostate tumors in PSMA, it is actively used for the delivery of various therapeutic and diagnostic agents, including nanosized drug delivery systems [21,22,23,24,25,26,27,28,29,30].

Recently, PSMA-specific imaging agents have significantly improved the visualization of prostate cancer (PCa) by single-photon emission computed tomography (SPECT) and positron emission tomography (PET). Several highly effective therapeutic and diagnostic monomodal conjugates have been developed, such as FDA-approved [^177^Lu]Lu-PSMA-617 [31], [^68^Ga]Ga-PSMA-11 [32], and [^18^F]-DCFPyL [33], suitable for both preoperative planning and postoperative evaluation. Nonetheless, their efficacy during surgical procedures is limited, as SPECT and PET agents lack the sub-millimeter resolution necessary to visualize positive tumor margins or micrometastases within the surgical field.

The precision of metastatic tumor tissue detection and excision determines patient prognosis and survival rates. The current intraoperative imaging modalities predominantly rely on anatomical orientation and visual identification by the surgeon. The utilization of fluorophore-containing agents that target PSMA facilitates more informed surgical decision-making. This advancement is achieved through intraoperative fluorescence imaging, which provides high-resolution contrast between tumorous and healthy tissues [34,35]. Moreover, the combination of moieties required for tumor detection and visualization within a single bimodal conjugate remains a promising strategy [36,37,38,39,40,41,42,43]. The key characteristics of bimodal PSMA-targeted conjugates loaded with a chelating fragment and a fluorescent label, suitable for diagnostics and imaging, were previously discussed in detail [44,45]. In this case, these characteristics are largely determined by the structural fragments incorporated. From the side of the fluorescent agent, it should have a large Stokes shift, as well as a high molar absorption coefficient and quantum yield. From the point of the chelating fragment or isotope binding motif, it must effectively bind the administered radioisotope, without the release of free radioisotopes during the biodistribution of the conjugate. Both these fragments must be photochemically (dye) and chemically (chelator) stable in biological solutions. Combining two fragments in a single conjugate that enables visualization using various methods may be promising in terms of creating an agent for the initial diagnosis of a tumor focus, followed by its fluorescence-guided removal. Recent publications have reported a limited number of bimodal conjugates containing isotope-binding motifs and fluorescent dyes. Many of these examples involve modifying the standard PSMA-617 conjugate vector platform, containing a DOTA chelator, by introducing a lysine moiety as a branching point for a fluorescent dye introduction [46,47]. Another study [48] presented an example of a modification of PSMA-617 in which the conventional DOTA chelator was replaced with a fluorine-binding fragment, enabling the use of the F-18 radioisotope in conjunction with the fluorescent dyes FAM and FITC. In addition, an example of the creation of the NODAGA-sCy7.5-PSMAi conjugate was presented, featuring a simplified PSMA-vector system with the chelating agent NODAGA and the sulfoCy7.5 fluorescent dye, which was used for the targeted delivery of copper radioisotopes [49]. In this study, we decided to use an alternative PSMA-based vector platform that we had previously developed to simultaneously deliver a fluorescent dye and a standard DOTA chelating moiety, and we evaluated its interaction with PSMA-expressing prostate cancer cells in in vitro and in vivo models.

## 2. Results and Discussion

### 2.1. Synthesis

**Scheme 1:**
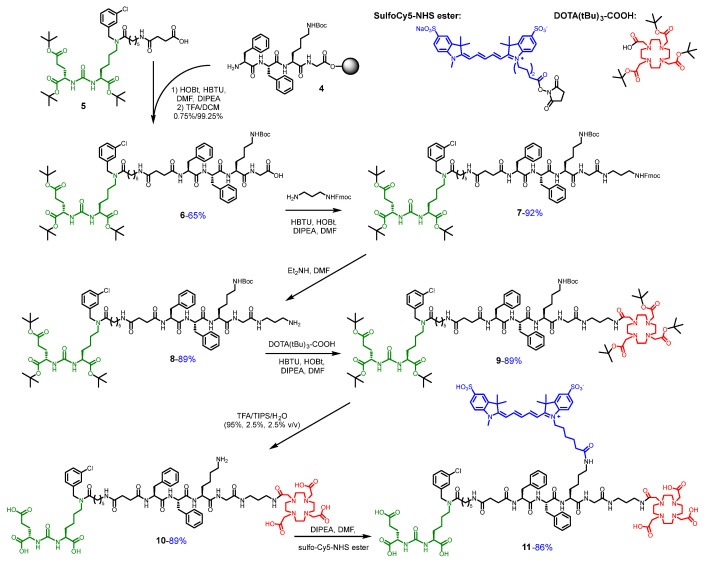
Synthesis of bimodal conjugate **10**, containing DOTA and SulfoCy5 fragments (green—Glu-urea-Lys (DCL) PSMA-vector; blue—SulfoCy5 dye; red—DOTA chelator.

Since the fluorescent label is the most labile fragment of the designed molecule, it should be introduced at the final stage of synthesis under mild conditions. Therefore, the precursor of the final compound must contain a chelating agent, free carboxyl groups in the urea fragment, and carboxyl groups in the chelating agent. This transformation can be achieved by a peptide synthesis reaction using an activated SulfoCy5-NHS ester on the ζ-NH_2_ group of the lysine fragment of the vector platform. The synthetic sequence for the double diagnostic conjugate **11** is shown in Scheme 1.

The synthesis of the starting building block **5** of the PSMA vector with the *m-chloro*-benzyl fragment was performed using previously described methods [50]. Peptide **4** was assembled using solid-phase peptide synthesis (SPPS) on 2-CTC (2-chlorotritylchloride resin) according to the scheme given in the Supplementary Materials (Scheme S1). The synthesis included the following steps: (1) immobilization of the N-substituted amino acid on a solid-phase substrate; (2) removal of the protective group; (3) modification of the NH_2_ group of the amino acid (steps 2 and 3 are repeated as many times as necessary to assemble the required peptide). Thus, peptide **4** was obtained: NH_2_-FFK(Boc)G-*2-CTC* (Scheme S1).

Next, vector fragment **5** was attached to the 2-CTC peptide NH_2_-FFK(Boc)G-*2-CTC* (**4**) immobilized on resin using activating agents HOBt/HBTU/DIPEA. After the reaction and washing of the solid-phase support, the modified peptide was removed from the polymer substrate by treatment with DCM/TFA (99.25–0.75%, *v*/*v*) (Scheme 1). As a result, compound **6** was isolated, which, according to ^1^H NMR, ^13^C NMR, LCMS, and HRMS spectra, is an individual substance. Then, the Fmoc-1,3-diaminopropane fragment was introduced into molecule **6** to synthesize molecule **11** (Scheme 1) or 3-aminopropylazide (Scheme S2) to obtain the final reference compound **14** (the complete scheme for obtaining this compound is given in the Supplementary Materials, Scheme S2). As a result of the reaction with Fmoc-1,3-diaminopropane, compound **7** was obtained with a yield of 92%, characterized by HRMS, LCMS, ^1^H NMR, and ^13^C NMR data.

Next, the Fmoc protecting group was removed by treating compound **7** with a mixture of Et_2_NH-DMF (20 equiv.) for 20 min, followed by purification by Et_2_O precipitation and reverse-phase column chromatography. Product **8** was isolated with 89% yield. For the bioconjugation reaction of compound **8** with DOTA(tBu)_3_-COOH, HBTU was used as the carboxyl group activating agent. As a result, compound **9** was obtained with a yield of 97%. The structure and purity of the product were confirmed by ^1^H NMR and ^13^C NMR data, HRMS, and LCMS.

Subsequent removal of *tert*-butyl and Boc protecting groups was performed by incubation with a mixture of TFA/TIPS/H_2_O (95%/2.5%/2.5%; *v*/*v*) for 4 h, which minimized the number of by-products. Purification was performed by reversed-phase column chromatography, and compound **10** was obtained with a yield of 92%.

The optimal near-infrared (NIR) dye should possess specific properties to ensure effective functionality. Primarily, a significant Stokes shift is crucial to minimize interference between the excitation and emission wavelengths. Furthermore, both the molar absorption coefficient and quantum yield must be sufficiently high to generate a robust fluorescent signal [51]. The dye must also exhibit water solubility to prevent aggregation in aqueous environments and demonstrate chemical stability and photostability in physiological conditions. Additionally, the dye molecule should incorporate appropriate functional groups to facilitate conjugation with targeting molecules. Typically, dyes exhibiting wavelengths within the near-infrared spectrum (650–900 nm) are employed for intraoperative visualization of different tumor types, such as breast cancer, soft tissue sarcomas, and metastatic lesions [52]. The utilization of NIR dyes offers several advantages, including superior tissue penetration depth relative to other dyes, diminished interference from blood within the surgical field, and low autofluorescence in the near-infrared range [53]. Various NIR dyes, such as Cy5, Cy7, Dylight800, derivatives of ICG, and IRDye800CW, have demonstrated significant potential in preclinical in vivo imaging studies [54]. Therefore, dyes with distinct characteristics can be appropriately selected for intraoperative imaging, with the final choice of the fluorescent label largely dependent upon the attributes of the detection camera utilized in the operating room. We selected the fluorescent dye SulfoCy5for tumor visualization because it has sufficiently polar fragments in the form of sulfo groups, sufficient Stokes’ shift, and fluorescence in the near-IR range. The SulfoCy5 fluorescent label was also chosen because it is readily available as an NHS ester and allows for comparison with the previously obtained PSMA-SulfoCy5 conjugate, including assessing the extent to which it would affect the biodistribution of synthesized conjugates. Similarly, this applies to the use of the chelating agent DOTA, for which conjugates with an *m*-Cl-benzyl derivative and an *L-Phe-L-Phe* linker had previously been studied, showing good accumulation in tumor tissues [30]. On the other hand, the choice of the chelating agent DOTA is based on its ability to chelate various metals, such as ^68^Ga, ^177^Lu, ^161^Tb, and copper isotopes. For this study, the most important factor was the effective binding of gallium isotopes by this chelating agent for further in vitro and in vivo investigations.

Thus, in the final step, a reaction was carried out between the SulfoCy5-NHS ester and compound **10,** and the final conjugate **11** was obtained with a yield of 86% (Scheme 1). The purity and structure of the product were confirmed by LCMS, HRMS, ^1^H NMR, and ^13^C NMR.

**Figure 1:**
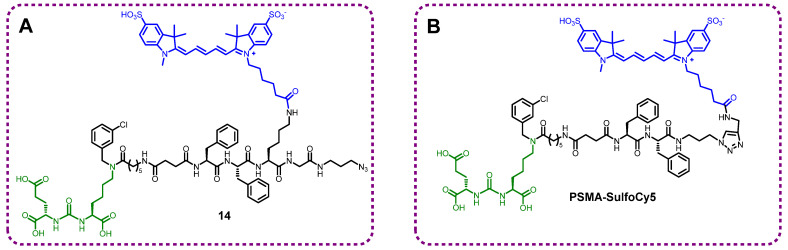
(**A**) Structure of monomodal conjugate **14** containing only SulfoCy5 residue; (**B**) the reference monomodal conjugate—PSMA-SulfoCy5.

Additionally, two reference compounds were synthesized for in vitro tests. A monomodal conjugate **14** (Figure 1) with a fluorescent label and a potentially functionalizable azido-group and the previously reported PSMA-SulfoCy5 [50] (the complete synthetic scheme is provided in the Supplementary Materials in Scheme S2).

### 2.2. Flow Cytometry

**Figure 2:**
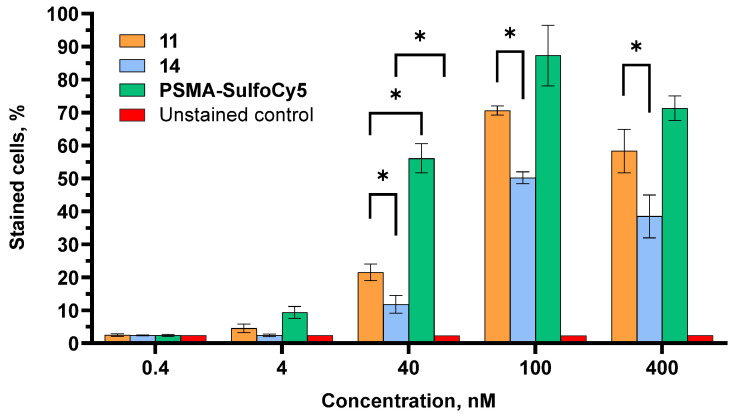
Results of imaging of LNCaP (PSMA+) cells with conjugates **11** and **14**, compared with the reference conjugate PSMA-SulfoCy5. Percentages of stained LNCaP cells. The samples are color-coded. Control—Autofluorescence of LNCaP cell lines. *—*p*-value < 0.05. Data are presented as mean ± SD.

The efficiency of PSMA-targeted conjugates was estimated with fluorescence-assisted flow cytometry. Analyses of the results showed that all the studied conjugates, together with the reference compound PSMA-SulfoCy5, stain the PSMA-expressing LNCaP prostate cancer cells. No or very low fluorescence signal was detected at 0.4 nM and 4 nM, whereas a dose-dependent increase in fluorescence intensity was observed at 40 nM and 100 nM, reaching a plateau between 100 nM and 400 nM. Although the reference conjugate PSMA-SulfoCy5 exhibited the highest fluorescence intensity, the newly synthesized conjugates demonstrated comparable staining of PSMA-expressing LNCaP cells in a nanomolar range of concentrations (Figure 2 and Figure S1). Importantly, the presence of DOTA in 11 did not result in a dramatic decrease in LNCaP binding, allowing further biological examination of the bimodal conjugate.

### 2.3. In Vivo Biodistribution Analysis

#### 2.3.1. Normal Mice Biodistribution

**Figure 3:**
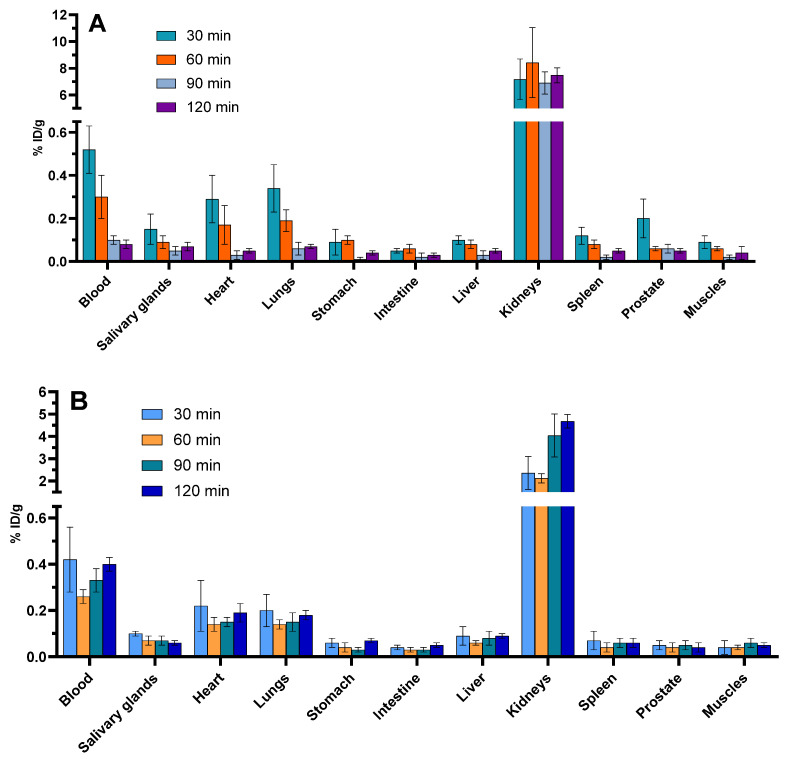
Biodistribution diagrams for [^68^Ga]Ga-**10** (**A**) and [^68^Ga]Ga-**11** (**B**) 30, 60, 90, and 120 min after drug administration, evaluated on a normal mouse model.

For compound **11** and its synthetic precursor **10**, a preliminary biological study was conducted to evaluate normal distribution in various organs in animal models in vivo (Figure 3, Table S1).

The results show that for conjugate **11**, the content in the bloodstream remains almost unchanged over time, unlike monoconjugate **10**, for which the maximum blood content is observed at 30 min and then steadily decreases. In addition, the concentration of bimodal conjugate **11** in the kidneys at all time points was significantly lower than that of compound **10**. Since accumulation in the kidneys and rapid elimination from the bloodstream are significant problems with most PSMA-targeted conjugates, compound **11** thus demonstrates potential for further detailed investigation.

#### 2.3.2. Xenograft Mice Model Biodistribution

**Figure 4:**
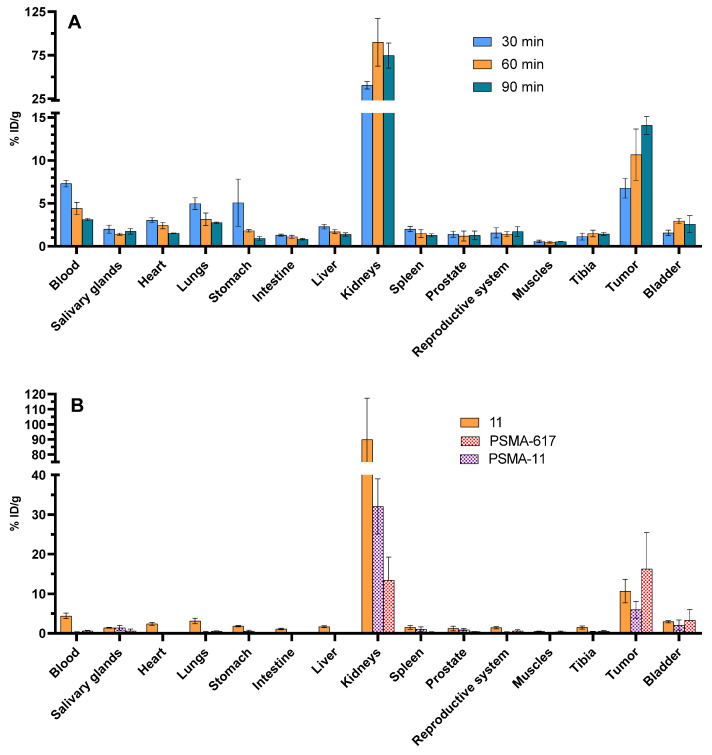
Biodistribution diagrams for [^68^Ga]Ga-**11** 30, 60, and 90 min after drug administration, evaluated on the LNCaP xenograft model (**A**) and comparison of biodistribution profiles for [^68^Ga]Ga-**11**, PSMA-617, and PSMA-11 60 min after drug administration (**B**), evaluated on the LNCaP xenograft model.

The distribution of the radiolabeled conjugate [^68^Ga]Ga-**11** in the bodies of Nu line mice with LNCaP line tumor xenografts is presented in Figure 4 (Table S2).

The distribution of the radiopharmaceutical [^68^Ga]Ga-**11** in the bodies of mice reveals several key pharmacokinetic patterns. The drug is rapidly eliminated from most organs and systems. This process is most intense in the stomach, where the level of radioactivity drops more than fivefold in just one hour—from 5.07% to 0.92%. A significant and stable decrease in concentration is also observed in the blood, heart, and lungs, which is typical for well-perfused tissues that do not express the target of the drug. At the same time, it is noteworthy that even 90 min after administration, a relatively high concentration of the drug remains in the blood—3.12%. This indicates a relatively slow overall clearance of the drug from the bloodstream, which may be due to its stability in plasma or binding to blood proteins. This fact is important for imaging: on the one hand, a high blood background may slightly reduce the contrast of the image, but on the other hand, it provides a longer “window” for delivering the drug to the tumor.

**Figure 5:**
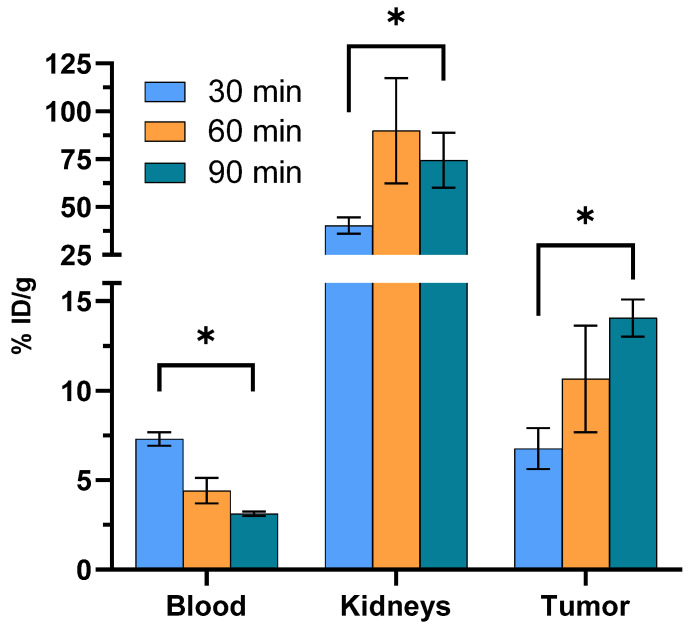
Distribution of the radiolabeled conjugate [^68^Ga]Ga-**11** in tissues with the most significant accumulation (blood, kidneys, and tumor). *—*p*-value < 0.05. Data are presented as mean ± SD. The most pronounced and extreme level of accumulation is observed in the kidneys. After just 30 min, the concentration in them reaches 40.39 ± 4.22%; after 60 min, it increases to 89.93 ± 27.44%, and even after 90 min, it remains incredibly high—74.45 ± 14.35%. This value exceeds the levels in other organs by an order of magnitude and is explained by the known phenomenon of receptor-specific fixation of PSMA ligands in renal tissue. The accumulation of conjugate **11** in renal tissue also exceeds the similar values of the reference conjugates PSMA-11 and PSMA-617 (Figure 4B).

However, against this background, several organs stand out where the drug is not simply retained but actively accumulates. The concentration of the radiopharmaceutical in the LNCaP tumor progressively and significantly increases throughout the experiment: from 6.76 ± 1.14% after 30 min to a statistically significant difference of 14.06 ± 1.04% after 90 min. This dynamic clearly demonstrates the drug’s ability to specifically bind to its target—the PSMA protein on the surface of tumor cells—and remain firmly attached to them, which is ideal behavior for a diagnostic agent (Figure 5). When comparing the accumulation data of the conjugate under study with standard PSMA-11 and PSMA-617 molecules 60 min after administration, it was found that [^68^Ga]Ga-**11** is inferior to PSMA-617 in terms of tumor accumulation but superior to the PSMA-11 conjugate in this respect (Figure 4B).

One of the approaches currently being actively developed to reduce renal accumulation in radiopharmaceutical conjugates involves the introduction of additional moieties that further influence the pharmacokinetics and biodistribution of the conjugate, in addition to its binding to PSMA on the surface of tumor cells due to the targeting properties of the PSMA vector. Among the introduced additional fragments, various albumin-binding motifs are the most effective [55,56,57,58]. The introduction of fluorescent dyes can also allow for the modulation of the pharmacokinetic parameters of targeted conjugates; however, as we can see, specifically in this example of conjugate **11**, this fluorescent dye does not exert a significant effect on the differential tumor/kidney accumulation, despite the initial optimistic results of the biodistribution of this conjugate in normal mouse models compared to its analogue conjugate **10** without the SulfoCy5 fluorescent dye. The fact is that the PSMA protein is present in large quantities not only in prostate tumors, but also in normal renal tubules, where the drug is directed, creating a powerful signal. For diagnostic imaging, this is a normal and expected pattern, but for potential therapeutic use (for example, when using isotopes such as lutetium-177, actinium-225, etc.), such intense accumulation in the kidneys is a major limiting factor due to the risk of damage to kidney tissue and the development of nephropathy with additional complications.

#### 2.3.3. Differential Accumulation Ratios

For a comparative analysis of accumulation between target organs and tissues to assess the possibility of contrast imaging using the radiolabeled conjugate [^68^Ga]Ga-**11** under study, differential accumulation coefficients were calculated and presented in Table 1.

The tumor/blood DAR values increased during the study, indicating migration of the radiolabeled conjugate [^68^Ga]Ga-**11** from the blood to the tumor xenografts, which can also be said about the tumor/muscle DAR values. However, DAR tumor/kidney values less than one throughout this study confirm that the kidneys will be visualized with greater contrast. In an analysis of absolute values, labeled conjugate [^68^Ga]Ga-**11** demonstrated comparable accumulation in tumor tissue compared to the reference compounds [^68^Ga]Ga-PSMA-11 and [^68^Ga]Ga-PSMA-617 60 min after administration. However, analysis of differential accumulation shows a greater accumulation of the conjugate under study in the blood after 60 min. At the same time, its nonspecific accumulation in muscle tissue is comparable to that of other compounds, and when compared to PSMA-11, the tumor/kidney DAR values are closely related. However, the tumor/kidney accumulation ratios differ significantly when compared to the PSMA-617 conjugate. The PSMA-617 conjugate, used for the targeted delivery of the radioisotope ^177^Lu, demonstrates significantly lower accumulation in renal tissue, both in absolute and relative terms. The current study has several limitations regarding its experimental scope that should be acknowledged. First, while the bimodal functionality of conjugate **11** was successfully validated in vitro using flow cytometry and in vivo via radiotracer biodistribution, we did not perform in vivo optical imaging. Future studies are required to evaluate the true potential of the SulfoCy5 moiety in an in vivo setting, such as fluorescence-guided tumor resection. Second, our in vivo evaluation was limited to a single PSMA-positive xenograft model (LNCaP). Testing the conjugate on a broader panel of tumor models with varying levels of PSMA expression would provide a more comprehensive assessment of its targeting sensitivity. Finally, although we observed high renal retention, the current study focused on the baseline pharmacokinetics and did not explore co-administration strategies (e.g., using competitive inhibitors) or structural modifications to mitigate this kidney uptake, which remains a critical direction for our future research.

Thus, the [^68^Ga]Ga-**11** drug demonstrates high specificity for the PSMA target, as confirmed by its effective accumulation in tumors and kidneys as well as its relatively rapid elimination from non-target tissues, making it a promising agent for PET imaging but not radionuclide therapy.

## 3. Materials and Methods

### 3.1. Synthesis

All solvents used were purified according to the procedures described in the book *Preparative Organic Chemistry* [59]. All starting compounds are commercially available reagents (Sigma-Aldrich (Taufkirchen, Germany), abcr (Karlsruhe, Germany), Carbosynth (Gardner, MA, USA), Lumiprobe (Moscow, Russia)) and were used without further purification. ^1^H NMR spectra were recorded on a Bruker Avance spectrometer (Bruker, Billerica, MA, USA) at 400 MHz, using CDCl_3_, DMSO-d6, and D_2_O as solvents. Chemical shifts are given in δ units with an accuracy of 0.01 ppm, and spin–spin interaction constants are given with an accuracy of 0.1 Hz using the residual solvent as an internal standard. ^13^C NMR spectra were recorded on a Bruker Avance spectrometer at 100.6 MHz, using DMSO-d6 and D_2_O as solvents. Chemical shifts are given in δ units with an accuracy of 0.01 ppm, using residual solvent as an internal standard.

For sample analysis, a Shimadzu Prominence LC-20 system (Shimadzu Corporation, Kyoto, Japan) was used with a Phenomenex Luna 3 μm C18 90A (150 × 4.6 mm) with a column thermostat at 40 °C and a fraction collector connected to a Shimadzu LCMS-2020 (Shimadzu Corporation, Kyoto, Japan) single quadrupole mass spectrometer with a dual ionization source (DUIS-ESI-APCI, ESI-APCI). Mobile phases are as follows: A—0.1% formic acid in water; B—9 mM ammonium formate in water; D—acetonitrile. Analytical liquid chromatography parameters are as follows: flow rate—1 mL/min (gradient: 0–0.5 min—5% D; 0.5–9.5 min—from 5% to 90% D; 9.5–12 min—90% D; 12–14.5 min—from 90% to 5% D) with optional UV detection for some compounds. Mass spectrometer parameters are as follows: gas flow rate of the desiccant 15.0 L/min, gas flow rate of the nebulizer 1.5 L/min, temperature of 250 °C, heating block temperature of 400 °C, interface voltage of 3.5 kV, and corona discharge voltage of 3.5 kV. Positive ions (mass range 250–2000 Da, in some cases 155–2000 Da) and negative ions (mass range 90–2000 Da) were registered simultaneously. The same chromatographic parameters were used for purification. The gradient was selected separately for each compound. Fractionation was based on UV detection. High-resolution mass spectra (HRMS) were recorded on a TripleTOF 5600+ quadrupole time-of-flight mass spectrometer (AB Sciex, Framingham, MA, USA), equipped with a TurboIon Spray ionization source and an LC-30 Nexera liquid chromatograph (Shimadzu, Kyoto, Japan). Samples were injected at a rate of 0.2 μL per sample into a 0.3 mL/min stream of methanol without chromatographic separation directly into the ion source. The spray voltage was ±5.5 kV, and the capillary temperature was 300 °C. The maximum injection time was 250 ms, averaged over 9 spectra, with a mass range of 100–3000 Da. For internal calibration, DMSO and diisooctyl phthalate signals (*m*/*z* 157.03515 and 413.26623) were used in positive mode, and dodecyl sulfate signal (*m*/*z* 265.14790) was used in negative mode.

Preparative chromatographic separation of substances was performed on an INTERCHIM puriFlash 4250 chromatograph (INTERCHIM, Montlucon Cedex, France). The solvent was evaporated using a rotary evaporator at reduced pressure at a bath temperature of 20–50 °C; column flash chromatography was performed using Macherey-nagel Silica gel 60 (MACHEREY-NAGEL, Dueren, Germany). The course of the reaction was monitored using thin-layer chromatography on a fixed layer of silica gel (TLC plates with pre-coated Merck silica gel 60 F254).

### 3.2. Flow Cytometry

The harvested cells were washed with PBS, dissociated using 1× trypsin-EDTA (Gibco, Grand Island, NY, USA), and washed three times with a PBS solution containing 3% Bovine Serum Albumin (BSA). A total of 4 × 10^5^ cells were stained with 0.4, 4, 40, 100, or 400 nM of each compound and diluted in a 3% BSA-containing PBS solution. Unstained control was treated with DMSO and diluted in the same ratio as the compounds. Cells were incubated at 4 °C for 1 h in a rotating rotor and then washed three times with a 3% BSA-containing PBS solution and suspended in PBS solution. Two biological replicates were obtained. Flow cytometry was performed using the SinoCyte X (provided by the Moscow State University Development Program NAUKA, Moscow, Russia) and analyzed with FlowJo v.10.8.1.

### 3.3. Conjugates Labeling with ^68^Ga

Compounds labeled with ^68^Ga were obtained using standard procedures described in article [60]. Briefly, 20 μg of a precursor, an aqueous solution of sodium acetate (0.6 M, 500 μL), and a conditioned (purified and concentrated) eluate from a ^68^Ge/^68^Ga generator (430 μL) were sequentially placed in an Eppendorf tube (Eppendorf SE, Hamburg, Germany). Hydrochloric acid with a concentration of 1.8 M (~70 μL) was used to regulate the pH value. The reaction mixture was incubated at 95 °C for 15 min. The radioactivity of the preparation was 30–100 MBq, and the pH was 4.2 ÷ 4.5.

The radiochemical purity of the obtained preparations was determined using radio-HPLC and radio-TLC methods. The main systems for TLC were as follows: iTLC-SG/50 mM H_3_Citraq; iTLC-SG/TFAaq 5% vol.; iTLC-SG/CH_3_OH-H_2_O (1:1), 1 M CH_3_COONH_4_. For HPLC, two methods are described in the previous paper [61]. In brief: a Knauer Smartline HPLC system (KNAUER, Berlin, Germany) equipped with an fLumo radiometric detector (Bertold, Bad Wildbad, Germany) and C18 reversed-phase columns was used. The column thermostat temperature was 40 °C. Method 1: 250 × 4.6 mm column (Jupiter, Phenomenex Inc., Torrance, CA, USA); isocratic flow (1.0 mL/min): 25% aqueous solution of TFA 0.1%, 75% acetonitrile. Method 2: 150 × 4.6 mm column (Advanced Chromatography Technologies Ltd., Aberdeen, UK) gradient flow (1.2 mL/min): 0–3–6–8–9–15 min = 100–100–0–0–100–100% A (A—0.05 M citric acid in water, B—acetonitrile). If necessary (e.g., if peaks other than unbound/colloidal ^68^Ga were observed after the labeling and quality control reaction), the preparations were transferred to a physiological saline solution using C18 Sep Pak cartridges (Waters, Milford, MA, USA).

### 3.4. In Vivo Evaluation of ^68^Ga Labeled Conjugates

#### 3.4.1. Cell Lines

To obtain xenograft models of prostate cancer, the PSMA+ human prostate cancer cell line LNCaP was selected. The LNCaP cell line is characterized by high levels of PSMA receptor expression [62]. Cell culture samples were obtained from certified cell culture banks. Frozen cell culture samples are stored in cryogenic storage. Cell cultures are cultivated under aseptic conditions in a CO_2_ incubator. Cell cultures are handled in a laminar flow cabinet, which protects the product from the operator. Tumor cells are cultivated and prepared for experiments in accordance with the recommendations provided by the cell culture bank upon receipt of cell samples.

The initial cell stock was thawed and transferred to T25 culture flasks (Corning, Corning, NY, USA) filled with complete growth medium recommended for a specific tumor cell line and prepared under aseptic conditions (Opti-MEM with the addition of fetal calf serum (10%) and antibiotics). The medium was conditioned by keeping it in a CO_2_ incubator (CO_2_ concentration 6%) for 15 min. After 24 h of incubation, the growth medium was replaced with fresh medium. As the cells grew and formed a monolayer, they were transferred to a larger number of culture flasks. To do this, the medium was removed, and 3 mL of a dissociating 0.25% trypsin-EDTA solution (PanEco, Moscow, Russia) was added to the flasks and left for 5 min. The completeness of cell detachment from the substrate was monitored using an inverted microscope. The detached cells were removed into labeled tubes and precipitated by centrifugation, and the supernatant was removed. The precipitated cells were resuspended in the prepared growth medium. Culture flasks were prepared and labeled, indicating the cell line, date of planting, and passage number. The flasks were filled with culture medium, and ~250,000 viable cells were added to each. The flasks were placed in an incubator and incubated until a complete monolayer was achieved. Two to three times a week, half of the growth medium was replaced with fresh medium. A complete monolayer contains about 5 million cells. This value is taken as the starting point when calculating the number of cells required for the experiment. The required number of cell monolayers was cultured, based on the fact that one animal would need to be injected with 4–6 × 10^6^ viable cells to model a tumor xenograft.

#### 3.4.2. Laboratory Animals

To evaluate the possibility of using coordination compounds for tumor visualization, biodistribution studies were conducted on animals with model prostate tumors. Sexually mature male Nu line mice, characterized by immunodeficiency caused by the absence of T lymphocytes, were selected as test systems. The animals were obtained from the state-certified laboratory animal breeding facility NPP “Pushchino” FIBS RAS. Immunodeficient animals were included in the experiments after a three-day adaptation period. Animal administration was carried out in accordance with the principles of bioethics [63] and GLP [64].

Immunodeficient mice were kept in plastic cages installed in a GA30CAGES 6LAYERS × 5 individually ventilated cage (IVC) system, which is equipped with a ventilation unit with a HEPA filter. Sterile granulated feed and distilled water were used for feeding. Access to water and feed was unrestricted. Air temperature and humidity were automatically maintained by the IVC system. The room was equipped with a UV air sterilizer. Animals were transported outside the IVC system in isolation cages. Work with animals was carried out in a laminar box, which protected the product from the operator, and in a specially designated surgical unit.

Human prostate cancer cells of the LNCaP line were cultured according to the cell bank recommendations to the required number based on the fact that one animal needs to be injected with ~5 × 10^6^ viable cells. On the day of modeling, the cells were removed from the surface of the flasks with a 0.25% trypsin-EDTA solution, and the entire batch of one line was combined in a sterile test tube. The cells were resuspended in the required amount of ready-made growth medium (Opti-MEM with the addition of fetal calf serum (10%) and antibiotics). The prepared cell suspension was dispensed into sterile syringes in 100 μL portions. The syringes were placed in a sterile box and transferred to a laminar box in the room where the transplantation procedure was performed on animals.

Cages containing the required number of animals were removed from the individually ventilated cage (IVC) system rack (3W, Beijing, China). The cages with animals were transferred to the room designated for the procedure and placed in a vis-à-vis laminar box (BMB-II-Laminar-S-1.2 (241.120) microbiological safety box, Laminar Systems, Moscow, Russia) for working with animals. All surfaces and operators’ hands were treated with an antiseptic solution. Nu/nu mice were removed from the isolation cage and placed on a sterile absorbent napkin. Carefully holding the mouse immobile, a subcutaneous injection of a suspension of LNCaP tumor cells (~5 × 10^6^ cells in 0.1 mL) was made into the scapular region. The subcutaneous location of the xenograft allows for non-invasive and highly accurate control of tumor growth [65]. The animals were then placed in a labeled cage, which was returned to the rack.

#### 3.4.3. Biodistribution and Pharmacokinetics Studies

To study the biodistribution of each conjugate, groups of mice (average body weight 27.2 ± 2.1 g) were formed, with 5 individuals for each time interval of biodistribution (30, 60, and 90 min after intravenous administration). Body weight and tumor size were used as determining factors for grouping the animals. Deviations in these parameters within the group did not exceed ±10%. The animals in each group were marked consecutively. The compounds under study were injected into the tail vein in a volume of 50 μL, which was optimal for reliable registration of radionuclide radiation by a gamma counter of volumetric activity. Thirty, 60, and 90 min after intravenous injection of [^68^Ga]-**11** conjugates, the mice were euthanized by decapitation after being placed under general anesthesia with a mixture of isoflurane and oxygen.

For radiometry, the following organ and tissue samples were selected in pre-weighed vials: (1) blood; (2) salivary glands; (3) heart; (4) lungs; (5) stomach; (6) intestines [small and large]; (7) liver; (8) kidneys; (9) spleen; (10) prostate; (11) reproductive system; (12) muscle tissue (thigh muscle); (13) bone tissue [tibia]; (14) bladder wall. For individual correction of the standard, the following was collected from each animal: (15) injection site (tail). Sample radiometry was performed using a Wizard 2480 automatic gamma counter (PerkinElmer, Shelton, CT, USA). The data obtained were used to calculate the proportion of radiopharmaceutical drug accumulation in organs (samples) using Formula (1). Radiopharmaceutical drug content was expressed as a percentage of the administered amount per organ and/or per 1 g of tissue (%/g):

(1) $$A=\frac{A_{sample}}{A_{ref}-A_{is}}\times 100,$$

where *A_sample_* is the sample count in cpm; *A_reference_* is the reference count (or the conversion of its aliquot count to 100% reference) in cpm. The reference count is adjusted for each animal individually by subtracting the count value of the “injection site” sample; *A_is_* is the “injection site” sample count in cpm.

The radiopharmaceutical conjugate content is expressed as a percentage of the amount administered to the entire organ and/or per 1 g of tissue (%/g). The following weight coefficients were used for calculations: the mass of all blood is 6.5% of the body weight of mice; the mass of all skeletal bone tissue is 10% of the body weight of the animal; the mass of all muscles is 45% of the body weight of the animal.

The *DARs* reflecting the specificity of accumulation are calculated using Formula (2):

(2) $$DAR=\frac{K_{pathology}}{K_{organ/tissue}}$$

where *K_pathology_* is the accumulation of the radiopharmaceutical drug in 1 g of tumor lesion; *K_organ/tissue_* is the accumulation of the radiopharmaceutical drug in 1 g of organ or tissue.

### 3.5. Statistical Analysis

The statistical significance evaluation was performed by the Mann–Whitney test using GraphPad Prism 9.0. (GraphPad Software, Inc., San Diego, CA, USA).

## 4. Conclusions

We synthesized a bimodal conjugate **11** containing the chelating agent DOTA and an additional fragment of a fluorescent dye SulfoCy5, which, on the one hand, can contribute to the visualization of tumor cells both in vitro and in vivo and, on the other hand, can influence the pharmacokinetic profile of the entire conjugate. To assess the influence of the chelating agent and of the fluorescent dye on characteristics of the conjugate, we synthesized two analogues of conjugate **11**: compound **14**, without a chelating fragment, and conjugate **10** without a fluorescent label. As previously reported, PSMA-11 and PSMA-617 were used as references in some experiments.

The bimodal conjugate **11** and the reference molecule PSMA-SulfoCy5 demonstrated similar binding to the PSMA-expressing LNCaP cells. In this case, the addition of the DOTA moiety resulted in only a negligible weakening effect on the binding efficiency of conjugate **11** to the PSMA-expressing cells.

The experimental results obtained on the in vivo biodistribution of the radiopharmaceutical conjugate [^68^Ga]Ga-**11** in mice with LNCaP line tumor xenografts allow us to identify its biodistribution profile and evaluate its potential as a diagnostic or theranostic agent.

The biodistribution profile of the radiolabeled conjugate [^68^Ga]Ga-**11** in mice with LNCaP tumor xenografts is as expected for PSMA ligands, with pronounced accumulation in the tumor (up to 14.06 %/g) and very high retention in the kidneys (up to 89.93%/g). Its key feature is a relatively slow clearance from the blood, resulting in moderate tumor/blood ratios. This compound served as a good base for further structural modification with the aim of improving the pharmacokinetic profile. This compound demonstrates classic PSMA ligand behavior without additional pharmacological modules. It provides stable accumulation in the tumor and high uptake in the kidneys, confirming its specificity for PSMA. However, moderate tumor/blood contrast ratios and the absence of pronounced renal clearance indicate that the fluorescent moiety does not significantly improve pharmacokinetics, serving primarily for imaging purposes.

## 5. Patents

Conjugates, synthesis, and their application were recently patented: RU2841078C1 and RU2823164C2.

## Figures and Tables

**Table 1:** Differential accumulation ratios (DARs) of the studied radiolabeled conjugate [^68^Ga]Ga-**11**, [^68^Ga]Ga-PSMA-11, and [^68^Ga]Ga-PSMA-617 in the body of Nu line mice with LNCaP tumor xenografts.

Time After [^68^Ga]Ga-11 Administration, min			[^68^Ga]Ga-X After 60 min		DAR
90	60	30	PSMA-11	PSMA-617	
4.50 ± 0.18	2.33 ± 0.30	0.94 ± 0.19	23.9 ± 16.6	26.2 ± 15.5	Tumor/blood
26.24 ± 1.43	24.64 ± 9.49	14.28 ± 5.91	37.4 ± 14.9	43.9 ± 36.9	Tumor/Muscles
0.20 ± 0.03	0.13 ± 0.02	0.17 ± 0.02	0.19 ± 0.08	1.21 ± 0.87	Tumor/Kidneys

## Data Availability

Data is contained within this article or the Supplementary Materials.

## Supplementary Materials

The following supporting information can be downloaded at https://www.mdpi.com/article/10.3390/ijms27083502/s1.

## Abbreviations

The following abbreviations are used in this manuscript:

DCL	(S)-2-(3-((S)-5-amino-1-carboxypentyl)ureido)pentanedioic acid;
DIPEA	N,N-Diisopropylethylamine;
DMSO	Dimethyl sulfoxide;
DOTA	2,2′,2″,2‴-(1,4,7,10-Tetraazacyclododecane-1,4,7,10-tetrayl)tetraacetic acid;
HBTU	N,N′,N′-Tetramethyl-O-(1H-benzotriazol-1-yl)uronium hexafluorophosphate;
HOBt	Hydroxybenzotriazole;
HRMS	High-resolution mass spectrometry;
LCMS	Liquid chromatography–mass spectrometry;
NHS	N-hydroxysuccinimide;
NMR	Nuclear magnetic resonance;
TFA	Trifluoroacetic acid;
TIPS	Triisopropyl silane.
