# Supplementary material for: Bimodal Fluorescent Conjugate Based on Prostate-Specific Membrane Antigen Ligands with the Chelating Agent DOTA and SulfoCy5 Dye: Synthesis, Radiolabeling, and Biological Activity
Source: Int J Mol Sci. 2026 Apr 14;27(8):3502. doi: 10.3390/ijms27083502 (PMC13116809; doi:10.3390/ijms27083502)
Supplement: Supplementary file 1 [file ijms-27-03502-s001.zip › ijms-4225303-supplementary.pdf]

Supporting information

# Bimodal fluorescent conjugate based on prostate-specific membrane antigen ligands with the chelating agent DOTA and SulfoCy5 dye: synthesis, radiolabeling, and biological activity.

*Machulkin A.E.* [a, c,*], *Petrov S.A.* [a], *Butakova N.S.* [a], *Lunev A.S.* [b], *Petrosova K.A.* [b], *Radik R. Shafikov* [a,d], *Dmitrii A. Skvortsov* [a], *Mitrofanov I.A.* [b], *Ivashkovskaya M.N.* [b], *Beloglazkina E.K.* [a], *Larenkov A.A.* [b].

[a] *Lomonosov Moscow State University, Chemistry Dept., Leninskie Gory, Building 1/3, GSP-1, Moscow, 119991, Russian Federation*

[b] *FSBI State Research Center–Burnasyan Federal Medical Biophysical Center of Federal Medical Biological Agency, Moscow, 123098, Russian Federation*

[c] *Department of Biochemistry, People's Friendship University of Russia Named After Patrice Lumumba (RUDN University), Moscow, 117198, Russian Federation.*

[d] *Shemyakin and Ovchinnikov Institute of Bioorganic Chemistry Russian Academy of Sciences, Moscow, 117997, Russia.*

**Keywords:** PSMA, prostate cancer, drug delivery, bimodal conjugates, DOTA-conjugates.

***Corresponding author.**

**E-mail address:** alekseymachulkin@rambler.ru.

## Synthesis

### *Synthesis of peptide sequence*

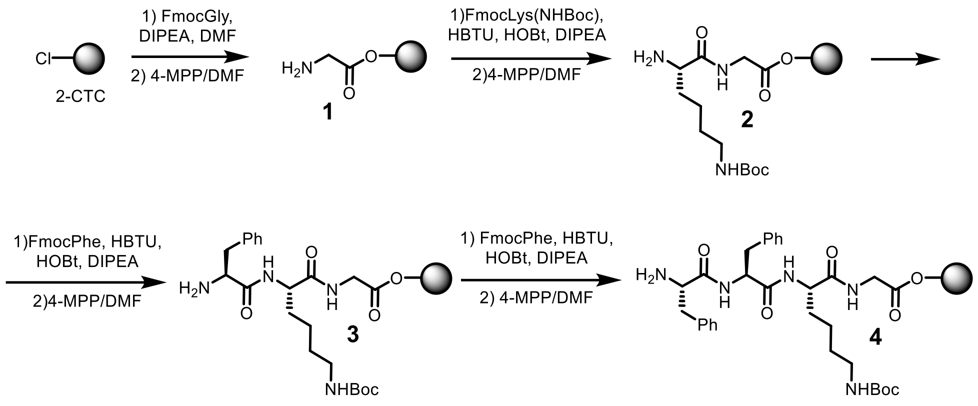


**Scheme S1.** Synthesis of peptide sequence on 2-CTC resin.

**General method for obtaining a peptide fragment 4 introduced to the PSMA-ligand linker by a solid-phase method**

**Activation of CTC-2.** 2-CTC resin (1 eq; 1.2-1.4 mmol/g; 100-200 mesh) was added to DCM (10 ml/1 g of resin) and stirred for 10 min, then purged with Ar, followed by dropwise addition of $SOCl_2$ (3 equiv.), then DMF (5% V/V to $SOCl_2$) was added and stirred at 40°C for 4 hours. The resin was filtered and transferred to a polypropylene reactor, washed with ×3 DMF (10 ml/1 g, 1 min), ×3 DCM (10 ml/1 g, 1 min). The amount of solvent for the reaction and washing was 10 ml/1 g of resin.

**Addition of the first amino acid residue.** To 2-CTC rsin (1 equiv; 1.2-1.4 mmol/g; 100-200 mesh) in DMF (10 ml/1 g), Fmoc-protected amino acid (2 equiv. relative to the upper capacity of CTC-2), DIPEA (10 equiv.) were added and left under stirring for 2 hours. The resin was then filtered, washed with ×3 MeOH (10 ml/1 g, 5 min), ×3 DCM (10 ml/1 g, 1 min), ×3 DMF (10 ml/1 g, 1 min), ×3 DCM (10 ml/1 g, 1 min).

**Removal of the Fmoc-group.** The peptide sequence on 2-CTC (1 equiv) was washed with ×2 DMF (10 ml/1 g, 1 min), then 4-methylpiperidine solution in DMF was added (20%/80% V/V, respectively, 15 ml) and left with stirring for 15 min, then the resin was filtered, washed with ×3 DMF (10 ml/1 g, 1 min), then 4-methylpiperidine in DMF (20%/80% V/V, respectively, 10 ml/1 g) and left to stir for 15 min. Afterwards, the resin was filtered off, washed ×3 times with DMF (10 ml/1 g, 1 min), ×3 times with DCM (10 ml/1 g, 1 min).

**Addition of the second and subsequent amino acid residues.** Fmoc-protected amino acid (2 equiv.), HOBt (0.5 equiv.), HBTU (2 equiv.), DIPEA (3 equiv.) to the peptide sequence on the 2-CTC resin (1 equiv.) in DMF (10 mL/1 g) and left it under stirring for 2 hours. The resin was then filtered off and washed with ×3 DMF (10 mL/1 g, 1 min) and ×3 DCM (10 mL/1 g, 1 min).

**$NH_2$-FFK(NHBoc)G-*2-CTC* - 4.**

From 2-CTC resin (1000 mg, 1.2-1.4 mmol), FmocGly-OH (0.832 g; 2.8 mmol), DIPEA (2.44 ml; 14 mmol) for attaching the first amino acid; FmocLys(L)(NHBoc)-OH (1.312 g; 2.8 mmol), HBTU (1.062 g; 2.8 mmol), HOBt (95 mg; 0.7 mmol) and DIPEA (0.73 mL; 4.2 mmol) for the attachment of the second amino acid; FmocPhe(L)-OH (1.085 g; 2.8 mmol), HBTU (1.062 g; 2.8 mmol), HOBt (95 mg; 0.7 mmol) and DIPEA (0.73 mL; 4.2 mmol) for the attachment of the third amino acid; FmocPhe(L)-OH (1.085 g; 2.8 mmol), HBTU (1.062 g; 2.8 mmol), HOBt (95 mg; 0.7 mmol) and DIPEA (0.73 ml; 4.2 mmol) for the attachment of the fourth amino acid; the amino acid sequence NH2-FFK(NHBoc)G-2-CTC – 4 was obtained.

***Synthesis of conjugate 11***

**Compound 5.**

Compound **5** was synthesized previously in the article[1].

**Synthesis of compound 6.**

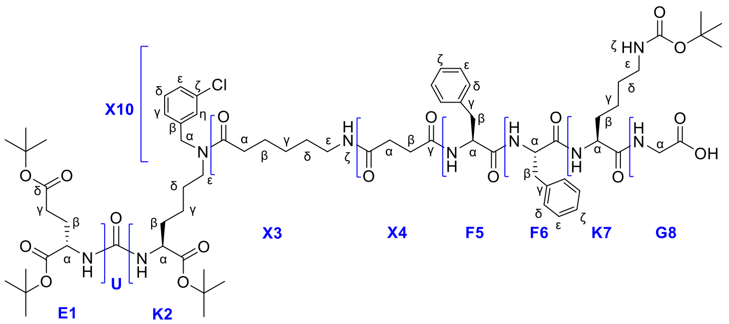


To the tetrapeptide **4** NH2-FFK(NHBoc)G- on 2-CTC resin (1 equiv.; 200 mg; 0.13 mmol) in DMF (5 mL) in a polypropylene reactor, **compound 5** (1.2 equiv.; 129 mg; 0.156 mmol), HOBt (0.5 equiv.; 9 mg; 0.066 mmol), HBTU (2 equiv.; 100 mg; 0.26 mmol), and DIPEA (3 equiv.; 71 µl; 0.4 mmol) were added. The mixture was left to stir for 4 hours. The solvent was removed by filtration on a porous reactor filter, and the resin was washed with DMF (3×5 ml), DCM (3×5 ml), and then dried from the solvent residue.

After that, a mixture of DCM/TFA (99.25%/0.75%, 6.5 ml) was added to the resin and left with stirring for 15 minutes, then the solution was filtered off from the resin. The solvent was removed under reduced pressure, and the residue was reevaporated three times with DCM (5 ml). The product was then purified by column chromatography (Puriflash, column with PF-15C18AQ-F0025 (15 µm, 40 g), eluent: $H_2O$(80%)/MeCN(20%) => $H_2O$(0%)/MeCN (100%)

for 15 minutes, followed by MeCN (100%) for 5 minutes. As a result compound **6** was obtained with 65% yield (121 mg).

**$^1$H NMR (400 MHz, DMSO-*d$_6$*, δ):**12.58 (br.s., 1H, G8COOH), 8.25-8.13 (m, 2H, F5NH+F6NH), 8.04 (t, J=5.9 Hz, G8NH), 7.94-7.82 (m, 2H, K7NH+X3NHk(*m*+*n*)), 7.42-7.09 (m, 14H, X10Hd*n*+X10He*n*+X10Hd*m*+ X10He*m*+F6He+F6Hd+X10Ht*mn*+F5He+F6Hk+F5Hk+F5Hd+X10Hg*mn*), 6.78 (t, J=5.1 Hz, 1H,K7NHk), 6.35-6.22 (m, 2H, K2NH+E1NH, *m*+*n*), 4.60-4.44 (m, 3H, F6Ha+X10Ha(*n*+*m*)), 4.43-4.33 (m, 1H, F5Ha), 4.30-4.20 (m, 1H, K7Ha), 4.08-4.00 (m, 1H, E1Ha), 4.00-3.90 (m, 1H, K2Ha), 3.77 (dd, J=16.3, 5.9 Hz, 1H, G8Ha(a)), 3.71 (dd, J=16.3, 5.9 Hz, 1H, G8Ha(b)), 3.21 (t, J=7.3 Hz, *n*) & 3.17 (t, J=7.3 Hz, *m*) (2H, K2He, *m*+*n, m*/*n*=3/2), 3.13-3.05 (m, 1H, F6Hb(a)), 3.05-2.81 (m, 6H, F6Hb(b)+X3He+K7He+F5Hb(a)), 2.69-2.58 (m, 1H, F5Hb(b)), 2.39-2.12 (m, 8H, X4Hb+E1Hg+X4Ha+X3Ha), 1.92-1.80 (m, 1H, E1Hb(a)), 1.77-1.12 (m, 19H, E1Hb(b)+K2Hb(a)+K7Hb(a)+ K7Hb(b)+K2Hb(b)+K7Hd+X3Hb+X3Hd +K2Hd+K2Hg+X3Hg, *m*+*n*), 1.40 -1.32 (m, 36H, tBu).

**$^{13}$C NMR (100 MHz, DMSO-*d$_6$*, δ)**: 172.27 (K2C(*n*)), 172.23 (K2C(*m*)), 172.20 (X3C(*n*)+X4Cg(*n*)), 172.19 (X3C(*m*)+X4Cg(*m*)), 171.96 (E1C), 171.79 (G8C), 171.50 (E1Cd+X4C), 171.40 (F6C), 171.09 (F5C), 170.85 (K7C), 157.19 (U(*m*)), 157.17 (U(*n*)), 155.63 (K7Boc), 141.19 (X10Cb(*m*)), 140.78 (X10Cb(*n*)), 138.06 (F6Cg), 137.96 (F5Cg), 133.45 (X10Ck(*n*)), 133.10 (X10Ck(*m*)), 130.64 (X10Cd(*n*)), 130.29 (X10Cd(*m*)), 129.20 (F6Cd), 129.11 (F5Cd), 128.16 (F6Ce), 128.06 (F5Ce), 127.23 (X10Ct(*m*)), 127.19 (X10Ce(*n*)), 126.89 (X10Ce(*m*)), 126.31 (X10Ct(*n*)+F6Ck), 126.27 (F5Ck), 126.09 (X10Cg(*m*)), 124.99 (X10Cg(*n*)), 80.64 (E1tBu), 80.47 (K2tBu(*m*)), 80.38 (K2tBu(*n*)), 79.83 (E1dtBu), 77.43 (K7BoctBu), 54.43 (F5Ca), 54.24 (F6Ca), 53.04 (K2Ca(*n*)), 52.89 (K2Ca(*m*)), 52.51 (K7Ca), 52.20 (E1Ca), 49.64 (X10Ca(*n*)), 47.12 (X10Ca(*m*)), 46.83 (K2Ce(*m*)), 45.25 (K2Ce(*n*)), 40.72 (G8Ca), 39.10 (K7Ce(*mn*), 38.67 (X3Ce(*m*)), 38.66 (X3Ce(*n*)), 36.97 (F5Cb), 36.94 (F6Cb), 32.35 (X3Ca(*n*)), 31.98 (X3Ca(*m*)), 31.87 (K7Cb), 31.83 (K2Cb), 30.94 (E1Cg), 30.82 (X4Ca), 30.68 (X4Cb), 29.31 (K7Cd), 29.10 (X3Cd(*m*)), 29.01 (X3Cd(*n*)), 28.32 (tBuK7), 27.78 (tBuE1), 27.68 (tBuK2+ K2Cd(*m*)), 27.66 (tBuE1d), 27.61 (E1Cb), 26.73 (K2Cd(*n*)), 26.33 (X3Cg(*m*)), 26.23 (X3Cg(*n*)), 24.78 (X3Cb(*m*)), 24.63 (X3Cb(*n*)), 22.60 (K7Cg), 22.46 (K2Cg(*n*)) 22.28 (K2Cg(*m*)).

**LCMS:** Purity - 100% (positive ions). Retention time – 13.079 min.

**ESI-MS**: $C_{72}H_{106}{}^{35}ClN_9O_{17}$: m/z calculated for $[M+2H^+]^{2+}$: 702.88, found: 703.40

**HRMS (m/z, ESI):** calculated for $C_{72}H_{106}{}^{35}ClN_9O_{17}$ - $[M+H]^+$ 1404.7468, found: 1404.7513.

**Synthesis of compound 7.**

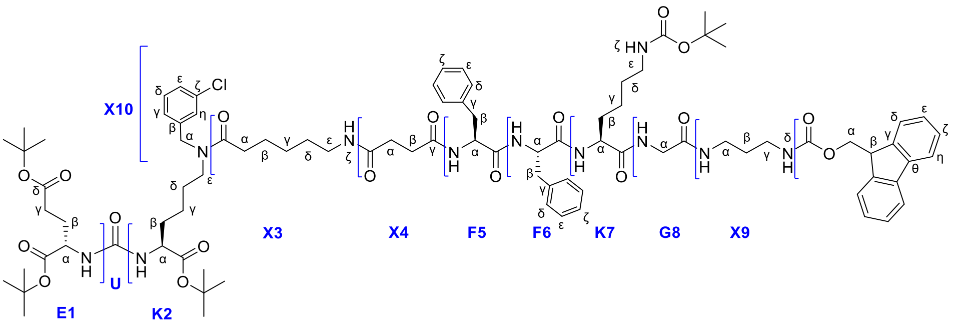


TFA×$NH_2(CH_2)_3$NHFmoc (1.01 equiv.; 47 mg; 0.114 mmol) was added to a solution of compound **6** (1 equiv.; 158 mg; 0.112 mmol) in 5 mL of DMF, followed by DIPEA (2.5 equiv.; 49 µl; 0.281 mmol) in 5 ml of DMF, and then HOBt (0.8 equiv.; 12 mg; 0.09 mmol), HBTU (1.3 equiv.; 55 mg; 0.146 mmol). The mixture was stirred for 12 hours under an inert atmosphere of argone. Next, the solvent was removed under reduced pressure and re-evaporated with 5 ml of DCM twice. Next, the residue was dissolved in 30 ml of DCM and extracted; 1) $H_2O$ (2×30 ml), 2) brine (2×30 ml). Then the organic fraction was dried over $Na_2SO_4$. After that, the solvent was removed. The residue was purified by column chromatography (Puriflash on a column (50µ 40g)); eluent: DCM(100%)/MeOH(0%) => DCM(90%)/MeOH(10%) for 30 minutes, followed by MeOH (100%) for 5 minutes. As a result, compound **7** was obtained as a pale yellow amorphous substance (169 mg, 92% yield).

**$^1$H NMR (400 MHz, DMSO-*d*$_6$, δ):** 8.25 (d, J=7.3 Hz, 1H, F5NH), 8.21 (d, J=8.3 Hz, 1H, F6NH*mn*), 8.00-7.94 (m, 1H, G8NH), 7.94-7.82 (m, 4H, X3NHk*n*+FmocHt+K7NH), 7.73-7.58 (m, 1H, X9NH+FmocHd), 7.45-7.35 (m, FmocHk +X10Hd*n*), 7.42-7.09 (m, 14H, X10Hd*n*+X10He*n*+X10Hd*m* +X10He*m*+F6He+F6Hd +X10Ht*mn*+ F5He+F6Hk+F5Hk+F5Hd+X10Hg*mn*), 6.77 (t, J=5.1 Hz, 1H,K7NHk), 6.34-6.21 (m, 2H, K2NH+E1NH, *m*+*n*), 4.59-4.41 (m, 3H, F6Ha+X9Ha(*n*+*m*)), 4.41-4.32 (m, 1H, F5Ha), 4.31-4.22 (m, 3H, FmocHa), 4.22-4.08 (m, 3H, FmocHb+K7Ha), 4.08-4.00 (m, 1H, E1Ha), 4.00-3.88 (m, 1H, K2Ha), 3.69 (dd, J=16.3, 5.9 Hz, 1H, G8Ha(a)), 3.60 (dd, J=16.3, 5.9 Hz, 1H, G8Ha(b)), 3.25-3.10 (m, 4H, K2He*mn*), 3.10-2.85 (m, 11H, F6Hb(a)+X9Hg+X3He(*mn*)+X9Ha +K7He+F6Hb(b)+ F5Hb(a)), 2.69-2.58 (m, 1H, F5Hb(b)), 2.38-2.12 (m, 8H, X4Hb+E1Hg+X4Ha+X3Ha), 1.92-1.80 (m, 1H, E1Hb(a)), 1.77-1.12 (m, 21H, E1Hb(b)+X9Hb+K2Hb(a)+K7Hb(a)+K7Hb(b)+K2Hb(b)+K7Hd +X3Hb+X3Hd+K2Hd+K2Hg+X3Hg, *m*+*n*), 1.40 -1.32 (m, 36H, tBu).

**$^{13}$C NMR (100 MHz, DMSO-$d_6$, δ)**: 172.51 (X4Cg(*nm*)), 172.25 (K2C(*n*)), 172.21 (K2C(*m*)), 172.14 (X3C(*n*)), 172.12 (X3C(*m*)), 171.94 (E1C), 171.68 (K7C), 171.61 (X4C), 171.54 (F6C), 171.46 (E1Cd), 171.30 (F5C), 168.53 (G8C), 158.94 (C(O)TFA), 158.59 (C(O)TFA), 157.15 (U(*m*)), 157.14 (U(*n*)), 156.15 (C(O)Fmoc), 155.61 (K7Boc), 143.94 (FmocCg), 141.17 (X10Cb(*m*)), 140.77 (X10Cb(*n*)+FmocCte), 137.98 (F6Cg), 137.95 (F5Cg), 133.44 (X10Ck(*n*)), 133.09 (X10Ck(*m*)), 130.61 (X10Cd(*n*)), 130.25 (X10Cd(*m*)), 129.12 (F6Cd), 129.06 (F5Cd), 128.16 (F6Ce), 128.04 (F5Ce), 127.64 (FmocCk), 127.22 (X10Ct(*m*)), 127.16 (X10Ce(*n*)), 127.10 (FmocCt), 126.88 (X10Ce(*m*)), 126.30 (F6Ck+X10Ct(n)), 126.23 (F5Ck), 126.07 (X10Cg(m)), 125.18 (FmocCe), 124.94 (X10Cg(*n*)), 120.15 (FmocCd), 80.59 (E1tBu), 80.42 (K2tBu(*m*)), 80.33 (K2tBu(*n*)), 79.78 (E1dtBu), 77.39 (K7BoctBu), 65.31 (FmocCa), 54.65 (F5Ca), 54.39 (F6Ca), 53.10 (K7Ca), 53.01 (K2Ca(*n*)), 52.87 (K2Ca(*m*)), 52.19 (E1Ca), 49.61 (X10Ca(*n*)), 47.11 (X10Ca(*m*)), 46.79 (FmocCb+K2Ce(*m*)), 45.22 (K2Ce(*n*)), 42.14 (G8Ca), 39.10 (K7Ce), 38.68 (X3Ce(*m*)), 38.59 (X3Ce(*n*)), 38.04 (X9Cg), 37.11 (F5Cb), 36.82 (F6Cb), 36.33 (X9Ca), 32.33 (X3Ca(*n*)), 31.95 (X3Ca(*m*)), 31.83 (K2Cb), 31.32 (K7Cb), 30.92 (E1Cg), 30.75 (X4Ca), 30.61 (X4Cb), 29.39 (X9Cb), 29.29 (K7Cd), 29.09 (X3Cd(*m*)), 29.00 (X3Cd(*n*)), 28.29 (tBuK7), 27.75 (tBuE1), 27.66 (tBuK2+ K2Cd(*m*)), 27.63 (tBuE1d+E1Cb), 26.71 (K2Cd(*n*)), 26.33 (X3Cg(*m*)), 26.23 (X3Cg(*n*)), 24.76 (X3Cb(*m*)), 24.61 (X3Cb(*n*)), 22.66 (K7Cg), 22.44 (K2Cg(*n*)) 22.26 (K2Cg(*m*)).

**LCMS:** Purity - 100% (positive ions). Retention time – 13.436 min.

**ESI-MS**: $C_{90}H_{124}ClN_{11}O_{18}$: m/z calculated for $[M+2H^+]^{2+}$: 842.45, found: 842.70

**HRMS (m/z, ESI):** calculated for $C_{90}H_{124}ClN_{11}O_{18}$ - $[M+Na^+]^+$ 1705.8738, found: 1705.8699

**Synthesis of compound 8.**

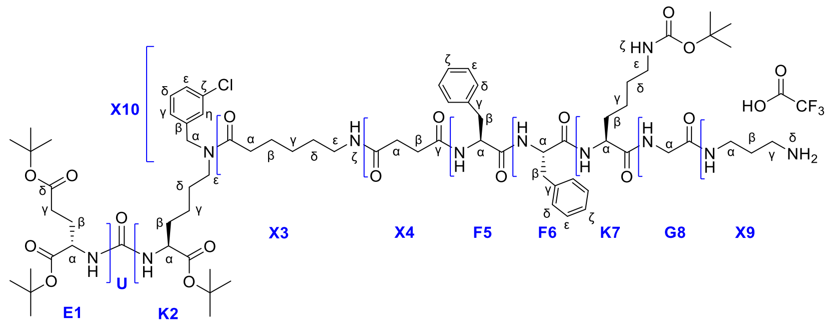


Compound **7** (1 equiv; 160 mg; 0.095 mmol) was dissolved in $Et_2NH$/DMF (20 equiv $Et_2NH$, 5 mL DMF) and stirred for 20 minutes under an inert atmosphere, then the solvent was removed under reduced pressure and reevaporated with 5 ml of DCM three times. The product was precipitated with 2 ml $Et_2O$ and washed twice with $Et_2O$ (2 mL). The residue was purified by reverse-phase column chromatography (Puriflash PF-15C18AQ-F0025 (15μ 40g), eluent:

$H_2O$×TFA(0.1%) (80%)/MeCN(20%) => $H_2O$×TFA(0.1%) (0%)/MeCN(100%) for 30 minutes followed by MeCN (100%) for 5 minutes. Compound **8** was obtained as a ×TFA salt in the form of a white amorphous substance (133 mg, 89% yield).

**$^1$H NMR (400 MHz, DMSO-*d$_6$*, δ):** 8.31 (d, J=7.3 Hz, 1H, F5NH), 8.23 (d, J=8.3 Hz, 1H, F6NH*mn*), 8.07-8.00 (m, 1H, G8NH), 7.97 (t, J=5.4 Hz, *m*) & 7.94 (t, J=5.4 Hz, *n*) (1H, X3NHk, *m*+*n*), 7.90-7.78 (m, 1H, K7NH+X9NH), 7.66 (br.s, 3H, X9$NH_3^+$d), 7.42-7.09 (m, 14H, X10Hd*n*+X10He*n*+X10Hd*m* +X10He*m*+F6He+F6Hd +X10Ht*mn*+ F5He+F6Hk+F5Hk+F5Hd+X10Hg*mn*), 6.79 (t, J=5.1 Hz, 1H,K7NHk), 6.34-6.21 (m, 2H, K2NH+E1NH, *m*+*n*), 4.60-4.41 (m, 3HF6Ha+X9Ha(*n*+*m*)), 4.41-4.32 (m, 1H, F5Ha), 4.18-4.08 (m, 1H, K7Ha), 4.08-4.00 (m, 1H, E1Ha), 4.00-3.88 (m, 1H, K2Ha), 3.69 (dd, J=16.3, 5.9 Hz, 1H, G8Ha(a)), 3.61 (dd, J=16.3, 5.9 Hz, 1H, G8Ha(b)), 3.25-3.10 (m, 4H, K2He*mn*+X9Hg), 3.10-2.85 (m, 7H, F6Hb(a)+X3He(*mn*)+K7He+F6Hb(b)+F5Hb(a)), 2.82-2.71 (m, 2H, X9Ha), 2.69-2.58 (m, 1H, F5Hb(b)), 2.38-2.12 (m, 8H, X4Hb+E1Hg+X4Ha+X3Ha), 1.92-1.80 (m, 1H, E1Hb(a)), 1.77-1.12 (m, 21H, E1Hb(b)+X9Hb+K2Hb(a)+K7Hb(a)+K7Hb(b)+K2Hb(b)+K7Hd +X3Hb+X3Hd+K2Hd+K2Hg+X3Hg, *m*+*n*), 1.40 -1.32 (m, 36H, tBu).

**$^{13}$C NMR (100 MHz, DMSO-*d$_6$*, δ)**: 172.73 (X4Cg(*nm*)), 172.27 (K2C(*n*)), 172.24 (K2C(*m*)), 172.15 (X3C(*nm*)), 171.96 (E1C), 171.84 (K7C), 171.69 (X4C), 171.64 (F6C), 171.49 (E1Cd), 171.45 (F5C), 169.14 (G8C), 158.94 (C(O)TFA), 158.59 (C(O)TFA), 157.20 (U(*m*)), 157.18 (U(*n*)), 155.64 (K7Boc), 141.19 (X10Cb(*m*)), 140.78 (X10Cb(*n*)), 138.02 (F6Cg), 137.98 (F5Cg), 133.46 (X10Ck(*n*)), 133.11 (X10Ck(*m*)), 130.62 (X10Cd(*n*)), 130.27 (X10Cd(*m*)), 129.13 (F6Cd), 129.06 (F5Cd), 128.21 (F6Ce), 128.09 (F5Ce), 127.22 (X10Ct(*m*)), 127.19 (X10Ce(*n*)), 126.89 (X10Ce(*m*)), 126.36 (F6Ck), 126.32 (X10Ct(*n*)), 126.29 (F5Ck), 126.09 (X10Cg(*m*)), 124.98 (X10Cg(*n*)), 80.60 (E1tBu), 80.42 (K2tBu(*m*)), 80.34 (K2tBu(*n*)), 79.80 (E1dtBu), 77.43 (K7BoctBu), 54.79 (F5Ca), 54.48 (F6Ca), 53.22 (K7Ca), 53.04 (K2Ca(*n*)), 52.90 (K2Ca(*m*)), 52.22 (E1Ca), 49.77 (X10Ca(*n*)), 47.14 (X10Ca(*m*)), 46.84 (K2Ce(*m*)), 45.25 (K2Ce(*n*)), 42.19 (G8Ca), 39.10 (K7Ce), 38.72 (X3Ce(*m*)), 38.69 (X3Ce(*n*)), 36.77 (F5Cb+F6Cb+ X9Cg+X9Ca), 32.33 (X3Ca(*n*)), 31.96 (X3Ca(*m*)), 31.81 (K2Cb), 31.24 (K7Cb), 30.94 (E1Cg), 30.78 (X4Ca), 30.59 (X4Cb), 29.32 (K7Cd), 29.10 (X3Cd(*m*)), 29.00 (X3Cd(*n*)), 28.31 (X9Cb+tBuK7), 27.76 (tBuE1), 27.67 (tBuK2+ K2Cd(*m*)), 27.65 (tBuE1d+E1Cb), 26.72 (K2Cd(*n*)), 26.34 (X3Cg(*m*)), 26.25 (X3Cg(*n*)), 24.76 (X3Cb(*m*)), 24.62 (X3Cb(*n*)), 22.69 (K7Cg), 22.45 (K2Cg(*n*)) 22.29 (K2Cg(*m*)).

**LCMS:** Purity - 100% (negative ions). Retention time – 19.203 min.

**ESI-MS**: $C_{75}H_{114}{}^{35}ClN_{11}O_{16}$: m/z calculated for $[M\text{-}H^+]^-$: 1458.8, found: 1458.8

**HRMS (m/z, ESI):** calculated for $C_{75}H_{114}{}^{35}ClN_{11}O_{16}$- $[M+H^{+}]^{+}$ 1460.8206, found: 1460.8217

**Synthesis of compound 9.**

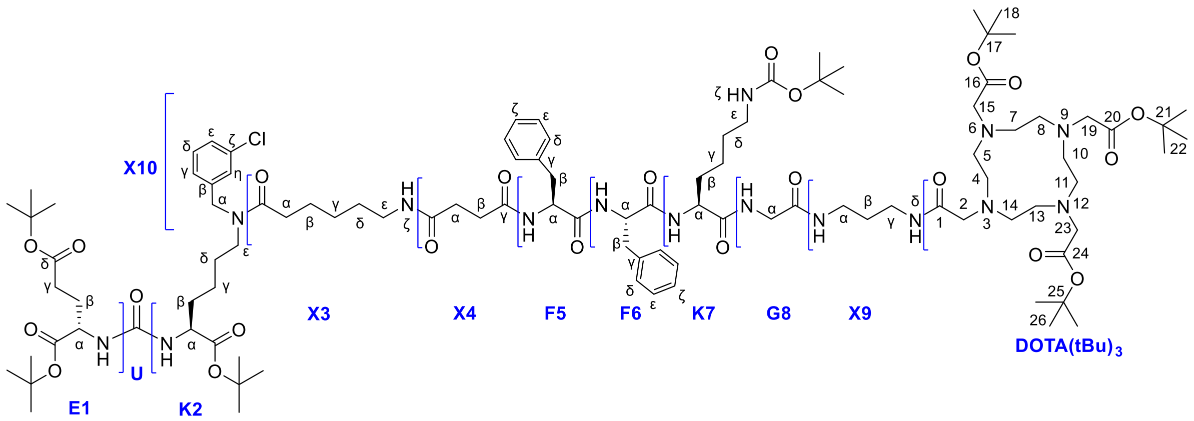


DIPEA (3 equiv; 17 µL; 95.31 µmol), HOBt (0.8 equiv; 3.5 mg; 25.42 µmol), HBTU (1.3 equiv; 16 mg; 41.3 µmol) were added to the solution of DOTA(tBu)3-COOH (1 equiv; 17 mg; 31.77 µmol) in DMF (5 mL). The mixture was stirred for 30 minutes, then the compound **8** (1 equiv; 50 mg; 31.77 µmol) was added and stirred for another 12 hours. The solvent was then removed under reduced pressure, the residue was dissolved in DCM (15 mL), and extraction was performed: 1) $H_2O$ (1×15 mL), 2) $NaHCO_3$ (2×15 mL). 2) saturated NaCl solution (1×15 mL). The organic fraction was dried over $Na_2SO_4$, then the solvent was removed under reduced pressure. The product was precipitated with petroleum ether (3 mL). Compound **9** was obtained as a white-yellow powder (62 mg, 97% yield).

**$^{1}$H NMR (400 MHz, DMSO-*d₆*, δ)**: 8.31 (d, J=7.3 Hz, 1H, F5NH), 8.23 (d, J=8.3 Hz, 1H, F6NH*mn*), 8.11-8.03 (m, 1H, G8NH), 7.99-7.87 (m, 2H, K7NH+X3NHk(*m*+*n*)), 7.87-7.77 (m, 1H, X9NH), 7.68 (t, J=5.5 Hz, X9NHd), 7.42-7.09 (m, 14H, X10Hd*n*+X10He*n*+X10Hd*m* +X10He*m*+F6He+F6Hd+X10Ht*mn*+F5He+F6Hk+F5Hk+F5Hd+X10Hg*mn*), 6.77 (t, J=5.1 Hz, 1H,K7NHk), 6.34-6.21 (m, 2H, K2NH+E1NH, *m*+*n*), 4.60-4.42 (m, 3HF6Ha+X9Ha(*n*+*m*)), 4.41-4.32 (m, 1H, F5Ha), 4.18-4.08 (m, 1H, K7Ha), 4.08-4.00 (m, 1H, E1Ha), 4.00-3.88 (m, 1H, K2Ha), 3.73-3.56 (m, 2H, G8Ha), 3.80-1.60 (br.m, 24H, DOTA), 3.25-3.10 (m, 4H, K2He*mn*+X9Hg), 3.10-2.85 (m, 9H, F6Hb(a)+X9Ha+X3He(*mn*)+ +K7He+F6Hb(b)+F5Hb(a)), 2.70-2.60 (m, 1H, F5Hb(b)), 2.40-2.10 (m, 8H, X3Ha*m*+X4Hb*mn*+E1Hg+X4Ha*mn* +X3Ha*n*), 1.92-1.80 (m, 1H, E1Hb(a)), 1.77-1.12 (m, 21H, E1Hb(b)+X9Hb+K2Hb(a)+K7Hb(a)+K7Hb(b)+K2Hb(b)+K7Hd +X3Hb+X3Hd+K2Hd+K2Hg+X3Hg, *m*+*n*), 1.43 (s, 9H, 22), 1.40 (s, 18H, 18+26), 1.40-1.34 (m, 27H, tBu).

**$^{13}$C NMR (100 MHz, DMSO-$d_6$, δ)**: 172.73 (X4Cg(*mn*)), 172.61 (1), 172.22 (K2C(*n*)+16+24+K2C(*m*)), 172.15 (X3C(*nm*)), 171.94 (E1C), 171.68 (X4C(*mn*)+K7C), 171.62 (F6C), 171.48 (E1Cd), 171.41 (20), 171.35 (F5C), 168.50 (G8C), 157.17 (U(*mn*)), 155.62 (K7Boc), 141.18 (X10Cb(*m*)), 140.77 (X10Cb(*n*)), 137.98 (F6Cg+F5Cg), 133.45 (X10Ck(*n*)), 133.10 (X10Ck(*m*)), 130.64 (X10Cd(*n*)), 130.27 (X10Cd(*m*)), 129.09 (F6Cd), 129.05 (F5Cd), 128.21 (F6Ce), 128.08 (F5Ce), 127.22 (X10Ct(*m*)), 127.18 (X10Ce(*n*)), 126.90 (X10Ce(*m*)), 126.36 (F6Ck), 126.30 (X10Ct(*n*)+F5Ck), 126.09 (X10Cg(*m*)), 124.98 (X10Cg(*n*)), 81.13 (21), 81.00 (17+25), 80.61 (E1tBu), 80.45 (K2tBu(*m*)), 80.36 (K2tBu(*n*)), 79.81 (E1dtBu), 77.42 (K7BoctBu), 55.87 (2), 55.38 (19), 55.27 (15+23), 54.84 (F5Ca), 54.53 (F6Ca), 53.17 (K7Ca), 53.02 (K2Ca(*n*)), 52.88 (K2Ca(*m*)), 52.20 (E1Ca), 49.62 (X10Ca(*n*)), 47.12 (X10Ca(*m*)), 46.82 (K2Ce(*m*)), 45.26 (K2Ce(*n*)), 42.11 (G8Ca), 39.50 (series of broad peaks, DOTAcyclic), 39.10 (K7Ce), 38.71 (X3Ce(*m*)), 38.66 (X3Ce(*n*)), 36.83 (F5Cb), 36.68 (F6Cb), 36.41 (X9Cg+X9Ca), 32.33 (X3Ca(*n*)), 31.96 (X3Ca(*m*)), 31.84 (K2Cb), 31.29 (K2Cb), 30.93 (E1Cg), 30.77 (X4Ca), 30.59 (X4Cb), 29.33 (K7Cd), 29.10 (X3Cd(*m*)), 29.01 (X3Cd(*n*)+X9Cb), 28.30 (tBuK7), 27.77 (tBuE1), 27.67 (22), 27.62 (18+26+tBuK2+K2Cd(*m*)+tBuE1d +(E1Cb), 26.72 (K2Cd(*n*)), 26.34 (X3Cg(*m*)), 26.24 (X3Cg(*n*)), 24.77 (X3Cb(*m*)), 24.64 (X3Cb(*n*)), 22.69 (K7Cg), 22.47 (K2Cg(*n*)) 22.28 (K2Cg(*m*)).

**LCMS:** Purity – 97.4% (positive ions). Retention time – 13.944 min.

**ESI-MS**: $C_{75}H_{114}ClN_{11}O_{16}$: m/z calculated for $[M+2H^+]^{2+}$: 1008.60, found: 1008.95

**HRMS (m/z, ESI):** calculated for $C_{103}H_{164}{}^{35}ClN_{15}O_{23}$- $[M+Na^+]^+$ 2037.1705, found: 2037.1684

**Synthesis of compound 10.**

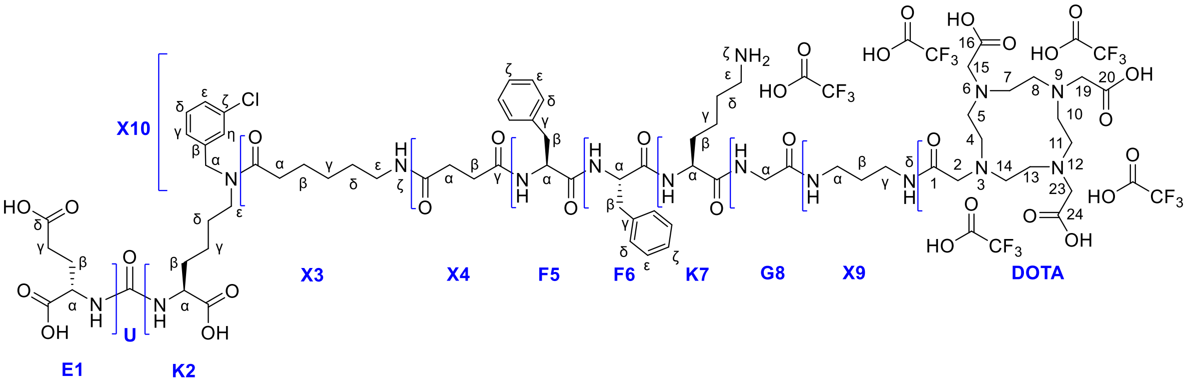


Compound **9** (1 equiv; 62 mg; 30.75 μmol) was dissolved in a mixture consisting of TFA/TIPS/$H_2O$ (95%/2.5%/2.5%, V = 4 ml). The mixture was stirred for 4 hours. The solvent was then removed. The product was precipitated with 2 ml of $Et_2O$ and washed twice with $Et_2O$ (2 ml). Then the residue was purified using reverse-phase chromatography (Puriflash PF-15C18AQ-F0012 (15μ 20g), eluent: $H_2O$×TFA(0.1%) (90%)/MeCN(10%) => $H_2O$×TFA(0.1%)

(0%)/MeCN(100%) for 30 minutes followed by MeCN (100%) for 5 minutes. Compound **10** was obtained as a white powder (60.5 mg, 92% yield) as a salt ×5 TFA.

**$^{1}$H NMR (400 MHz, DMSO-*d$_6$*, δ)**: 8.50-8.40 (m, 1H, X9NHd), 8.27 (d, J = 7.5 Hz, 1H, F5NH*mn*), 8.23 (d, J = 8.3 Hz 1H, F6NH*mn*), 8.02-7.87 (m, 3H, G8NH+K7NH+X3NHk*m*+*n*), 7.84-7.76 (m, 1H, X9NH), 7.76-7.64 (br.s., 3H, K7NHk), 7.42-7.10 (m, 14H, X10Hd*n*+X10He*n*+X10Hd*m* +X10He*m*+F6He +F6Hd+X10Ht*mn*+F5He+F6Hk +F5Hk+F5Hd +X10Hg*mn*), 6.39-6.25 (m, 2H, K2NH+E1NH, *m*+*n*), 4.60-4.41 (m, 3H, F6Ha+X10Ha(*n*+*m*)), 4.41-4.31 (m, 1H, F5Ha), 4.24-4.14 (m, 1H, K7Ha), 4.14-3.97 (m, 4H, E1Ha+2+K2Ha), 3.97-3.82 (br.s, 2H, 19), 3.76-3.46 (m, 6H, G8Ha+15+23), 3.45-3.22 (br.m, 8H, Hcyclic), 3.22-2.83 (m, 19H, K2He*mn*+F6Hb(a)+Hcyclic+X9Hg+X9Ha+X3He(*mn*) +F6Hb(b)+F5Hb(a)), 2.80-2.70 (m, 2H, K7He), 2.69-2.58 (m, 1H, F5Hb(b)), 2.40-2.10 (m, 8H, X3Ha*m*+X4Hb*mn*+E1Hg+X4Ha*mn*+ X3Ha*n*), 1.98-1.84 (m, 1H, E1Hb(a)), 1.77-1.12 (m, 21H, E1Hb(b)+K2Hb(a))+X9Hb +K7Hb(a)+K7Hb(b)+K2Hb(b)+K7Hd+X3Hb+X3Hd+K2Hd+K2Hg+X3Hg+K7Hg, *m*+*n*).

**$^{13}$C NMR (100 MHz, DMSO-*d$_6$*, δ)**: 174.58 (K2C(*n*)), 174.55 (K2C(*m*)), 174.25 (E1C(*nm*)), 173.82 (E1Cd), 172.53 (X4Cg(n)), 172.51 (X4Cg(m)), 172.22 (X3C(*nm*)), 171.83 (broad peak 16+24), 171.67 (K7C), 171.65 (F5C), 171.58 (X4C), 171.33 (F6C), 169.71 (br., 20), 168.70 (G8C), 165.20 (br., 1), 158.64 (C(O)TFA), 158.29 (C(O)TFA), 157.37 (U), 141.27 (X10Cb(*m*)), 140.86 (X10Cb(*n*)), 138.03 (F6Cg), 137.97 (F5Cg), 133.45 (X10Ck(*n*)), 133.10 (X10Ck(*m*)), 130.66 (X10Cd(*n*)), 130.29 (X10Cd(*m*)), 129.16 (F6Cd), 129.09 (F5Cd), 128.24 (F6Ce), 128.12 (F5Ce), 127.23 (X10Ct(*m*)), 127.18 (X10Ce(*n*)), 126.90 (X10Ce(*m*)), 126.41 (F6Ck), 126.32 (X10Ct(n)+F5Ck), 126.13 (X10Cg(m)), 125.01 (X10Cg(*n*)), 117.64 ($CF_3$), 114.72 ($CF_3$), 54.70 (F5Ca+19), 54.41 (F6Ca), 54.03 (br., 19), 52.75 (K7Ca), 52.57 (br., 15+23), 52.33 (K2Ca(*n*)), 52.21 (K2Ca(*m*)), 51.73 (E1Ca), 50.54 (series of broad peaks, DOTA-cycle.), 49.67 (X10Ca(*n*)), 48.37 (series of broad peaks, DOTA-cycle), 47.96 (series of broad peaks, DOTA-cycle.), 47.19 (X10Ca(*m*)), 46.92 (K2Ce(*m*)), 45.22 (K2Ce(*n*)), 42.08 (G8Ca), 38.72 (K7Ce+X3Ce(*m*)), 38.63 (X3Ce(*n*)), 36.95 (F5Cb), 36.80 (F6Cb+X9Cg), 36.42 (X9Ca), 32.34 (X3Ca(*n*)), 31.92 (X3Ca(*m*)), 31.82 (K2Cb), 31.05 (K7Cb), 30.81 (X4Ca), 30.64 (X4Cb), 29.97 (E1Cg), 29.14 (X3Cd(*m*)), 29.05 (X3Cd(*n*)), 28.82 (X9Cb), 27.82 (K2Cd(*m*)), 27.57 (E1Cb), 26.70 (K2Cd(*n*)+K7Cd), 26.33 (X3Cg(*m*)), 26.26 (X3Cg(*n*)), 24.78 (X3Cb(*m*)), 24.63 (X3Cb(*n*)), 22.55 (K7Cg) 22.39 (K2Cg(n)), 22.20 (K2Cg(*m*)).

**LCMS:** Purity – 100% (positive ions). Retention time – 12.172 min.

**ESI-MS**: $C_{74}H_{108}ClN_{15}O_{21}$: m/z calculated for $[M+2H^+]^{2+}$: 789.88, found: 790.65

**HRMS (m/z, ESI):** calculated for $C_{74}H_{108}{}^{35}ClN_{15}O_{21}$- $[M+K^+]^+$ 1616.7164, found: 1616.7115

**Synthesis of compound 11.**

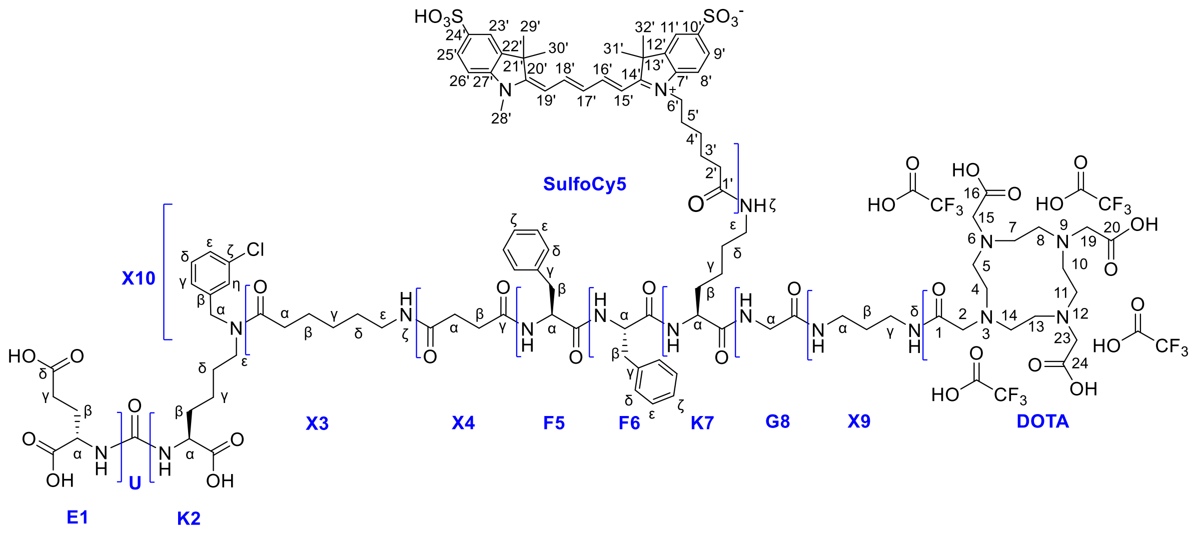


Compound **10** (1 equiv; 20 mg; 9.3 μmol) and DIPEA (10 equiv; 12 mg; 16.2 μmol) were dissolved in DMF (2 mL), and the mixture was purged with argon. Sulfo-Cy5 NHS-ester (1.05 equiv; 7.6 mg; 9.77 μmol) was added to the mixture. The mixture was stirred for 12 hours, after which the solvent was evaporated under reduced pressure. The residue was purified by column chromatography (Puriflash on a PF-15C18AQ-F0012 column (15 μm, 20 g), eluent: $H_2O$ (90%)/MeCN (10%) => $H_2O$ (0%)/MeCN (100%). Within 20 min after MeCN (100%) for 5 min, compound **11** was obtained as a blue powder (21.4 mg, 86% yield) as a salt ×4 TFA.

**$^1$H NMR (600 MHz, DMSO-$d_6$, δ)**: 12.62 (br.s., COOH), 8.48-8.12 (m, 5H, X9NHd+18'+16'+F5NH+F6NH), 8.02-7.89 (m, 2H, G8NH+X3NHk(*m*+*n*)), 7.88-7.78 (m, 3H, K7NH+11'+23'), 7.78-7.67 (m, 2H, K7NHk+X9NH), 7.67-7.60 (m, 2H, 9'+25'), 7.42-7.09 (m, 16H, X10Hd*n*+X10He*n*+X10Hd*m*+8'+26'+X10He*m*+F6He+F6Hd+X10Ht*mn*+F5He+ F6Hk+F5Hk+F5Hd+X10Hg*mn*), 6.55 (t, J = 12.2 Hz, 1H, 17'), 6.40-6.19 (m, 4H, K2NH+19'+E1NH+15', *m*+*n*), 4.60-4.41 (m, 3H, F6Ha+X10Ha(*n*+*m*)), 4.40-4.30 (m, 1H, F5Ha), 4.25-3.94 (m, 7H, K7Ha+E1Ha+6'+2+K2Ha), 3.94-3.77 (m, 2H, 19), 3.73-3.22 (m, 14H, G8Ha+15+23+Hcyclic), 3.58 (s, 3H, 28'), 3.24-2.85 (m, 21H, K2He*mn*+F6Hb(a)+Hcyclic +X9Hg+X9Ha +X3He(*mn*)+K7He+F6Hb(b)+F5Hb(a)), 2.70-2.59 (m, 1H, F5Hb(b)), 2.40-2.10 (m, 8H, X4Hb+E1Hg+X4Ha+X3Ha), 2.02 (t, J = 6.7 Hz, 2H, 2'), 1.95-1.85 (m, 1H, E1Hb(a)), 1.77-1.68 (m, 1H, E1Hb(b)), 1.68 (S, 12H, 29'+30'+31'+32'), 1.67-1.12 (m, 26H, X9Hb+K2Hb(a)+K7Hb(a)+K7Hb(b) +5'+K2Hb(b)+K7Hd+X3Hb +3'+X3Hd+K2Hd +4'+K2Hg +X3Hg+K7Hg, *m*+*n*).

**$^{13}$C NMR (100 MHz, DMSO-$d_6$, δ)**: 174.49 (K2C(*n*)), 174.45 (K2C(*m*)), 174.16 (E1C(*nm*)), 173.78 (20'), 173.73 (E1Cd), 172.80 (14'), 172.52 (X4Cg(n)), 172.49 (X4Cg(m)), 172.15 (X3C(*mn*)), 171.83 (br. peak, 16+24), 171.78 (1'), 171.48 (K7C), 171.57 (F5C), 171.52 (X4C),

171.26 (F6C), 168.66 (G8C), 168.61 (br., 20), 165.20 (br., 1), 158.25 (C(O)TFA), 157.93 (C(O)TFA), 157.28 (U), 154.26 (18'), 154.17 (16'), 145.30 (24'), 144.83 (10'), 142.74 (27'), 142.18 (7'), 141.20 (X10Cb(*m*)), 140.80 (X10Cb(*n*)), 140.49 (22'), 140.46 (12'), 137.96 (F6Cg), 137.92 (F5Cg), 133.37 (X10Ck(*n*)), 133.02 (X10Ck(*m*)), 130.59 (X10Cd(*n*)), 130.22 (X10Cd(*m*)), 129.09 (F6Cd), 129.01 (F5Cd), 128.16 (F6Ce), 128.04 (F5Ce), 127.16 (X10Ct(*m*)), 127.11 (X10Ce(*n*)), 126.82 (X10Ce(*m*)), 126.32 (F6Ck), 126.26 (X10Ct(n)+F5Ck), 126.06 (X10Cg(m)+25'), 126.02 (9'), 125.68 (17'), 124.95 (X10Cg(*n*)), 119.95 (11'), 119.85 (23'), 110.13 (8'+26'), 103.69 (15'), 103.36 (19'), 54.66 (F5Ca+19), 54.38 (F6Ca), 53.94 (br., 2), 53.02 (K7Ca), 52.53 (br., 15+23), 52.26 (K2Ca(*n*)), 52.15 (K2Ca(*m*)), 51.66 (E1Ca), 50.55 (series of broad peaks, DOTA-cycle.), 49.63 (X10Ca(*n*)), 48.90 (21'), 48.84 (13'), 48.32 (series of broad peaks, DOTA-cycle), 47.98 (series of broad peaks, DOTA-cycle), 47.16 (X10Ca(*m*)), 46.87 (K2Ce(*m*)), 45.32 (K2Ce(*n*)), 43.36 (6'), 42.09 (G8Ca), 38.66 (X3Ce(*m*)), 38.59 (X3Ce(*n*)), 38.23 (K7Ce), 36.85 (F5Cb), 36.71 (F6Cb+X9Cg), 36.33 (X9Ca), 35.09 (2'), 32.27 (X3Ca(*n*)), 31.86 (X3Ca(*m*)), 31.76 (K2Cb), 31.24 (28'), 31.18 (K7Cb), 30.76 (X4Ca), 30.57 (X4Cb), 29.90 (E1Cg), 29.06 (X3Cd(*m*)), 28.95 (X3Cd(*n*)), 28.86 (5'), 28.73 (X9Cb), 27.78 (K2Cd(*m*)), 27.52 (E1Cb), 27.12 (29'+30'), 26.92 (31'+32'), 26.73 (K2Cd(*n*)), 26.68 (K7Cd), 26.26 (X3Cg(*m*)), 26.18 (X3Cg(*n*)), 25.67 (4'), 24.92 (3'), 24.71 (X3Cb(*m*)), 24.56 (X3Cb(*n*)), 22.65 (K7Cg), 22.50 (K2Cg(*n*)) 22.33 (K2Cg(*m*)).

**LCMS:** Purity - 100% (positive ions). Retention time – 10.837 min.

**ESI-MS**: $C_{106}H_{144}ClN_{17}O_{28}S_2$: m/z calculated for $[M+3H^+]^{3+}$: 735.32, found: 735.65.

.

### *Synthesis of conjugate 14*

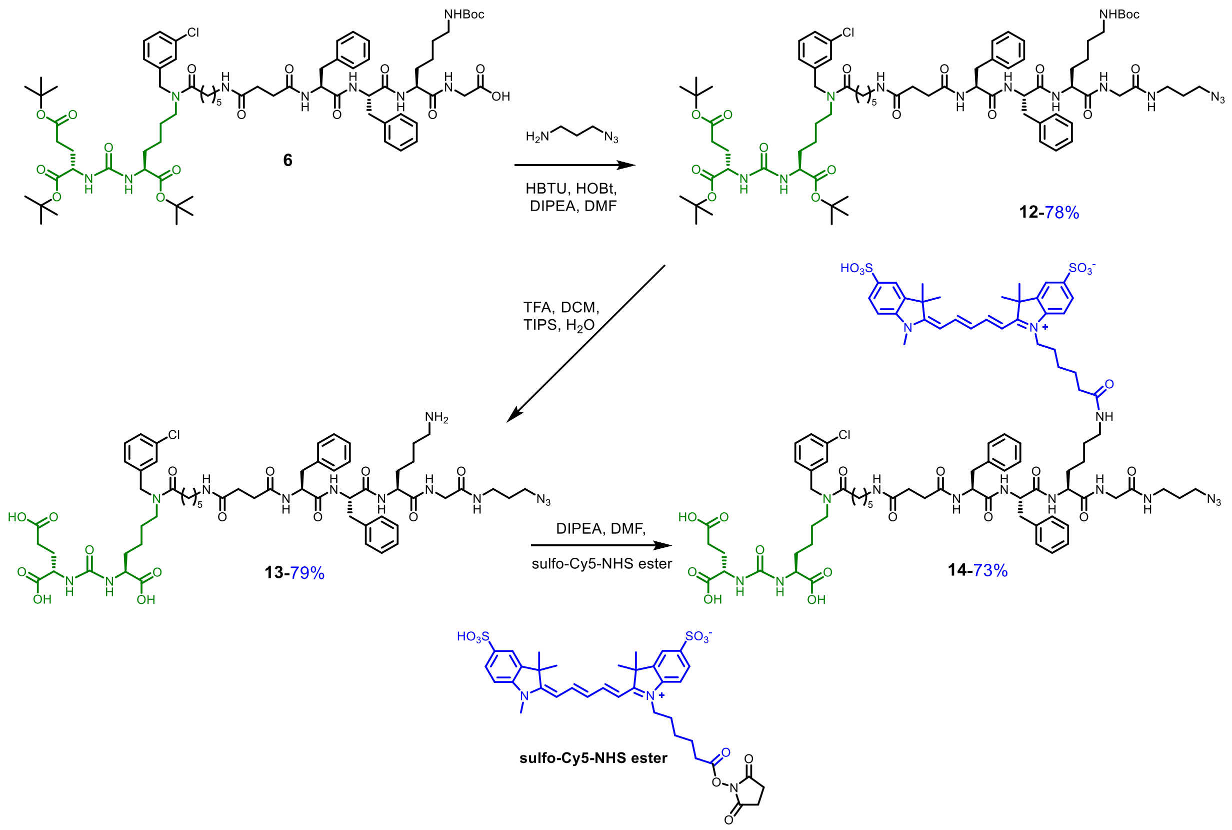


**Scheme S2.** Synthetic scheme of a reference monomodal conjugate **14** obtaining.

**Synthesis of compound 12.**

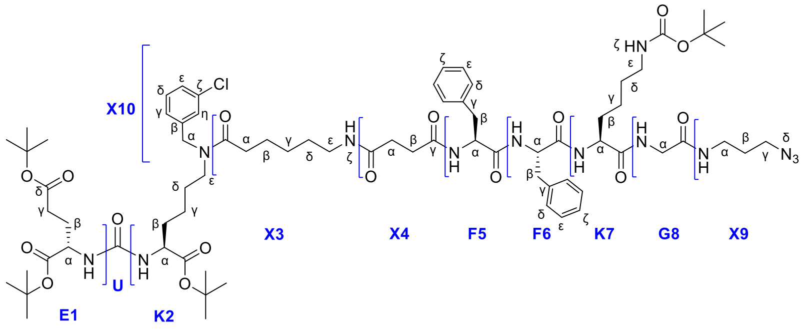


DIPEA (2 equiv.; 32 µl; 0.185 mmol), HOBt (1.5 equiv.; 19 mg; 0.139 mmol) and HBTU (1.5 equiv; 53 mg; 0.139 mmol) were added to the solution of compound **6** (1 equiv; 130 mg; 0.093 mmol) in DMF (10 mL). The mixture was stirred for 30 min in an inert atmosphere of argon. Then $NH_2$-$(CH_2)_3$-$N_3$ (2 equiv; 18 mg; 0.185 mmol) was added and the mixture was left to stir for 12 hours. The solvent was then removed under reduced pressure and twice reevaporated with DCM. The residue was dissolved in 20 ml of DCM and extracted; 1) $H_2O$ (2×30 ml), 2) saturated NaCl solution (2×30 ml). The organic fraction was then dried over $Na_2SO_4$. The solvent was then removed. The residue was further purified by column chromatography (Puriflash on PF-15C18AQ-F00040 column (15 µm 40g); eluent: $H_2O$(80%)/MeCN(20%) => $H_2O$(0%)/MeCN

(100%) for 10 minutes, followed by MeCN (100%) for 5 minutes. As a result compound **12** was obtained with 78% yield (108 mg).

**$^1$H NMR (400 MHz, DMSO-*d$_6$*, δ):** 8.28 (d, J=7.7 Hz, 1H, F5NH), 8.20 (d, J=7.6 Hz, 1H, F6NH), 7.99 (t, J=5.9 Hz, G8NH), 7.95-7.84 (m, 2H, K7NH+X3NHk(*m*+*n*)),7.73 (t, J=5.5 Hz, X10NH), 7.42-7.09 (m, 14H, X10Hd*n*+X10He*n*+X10Hd*m* +X10He*m*+F6He+F6Hd+X10Ht*mn*+F5He+F6Hk+F5Hk+F5Hd+X10Hg*mn*), 6.78 (t, J=5.1 Hz, 1H,K7NHk), 6.34-6.21 (m, 2H, K2NH+E1NH, *m*+*n*), 4.60-4.42 (m, 3HF6Ha+X9Ha(*n*+*m*)), 4.41-4.32 (m, 1H, F5Ha), 4.18-4.08 (m, 1H, K7Ha), 4.08-4.00 (m, 1H, E1Ha), 4.00-3.88 (m, 1H, K2Ha), 3.70 (dd, J=16.3, 5.9 Hz, 1H, G8Ha(a)), 3.60 (dd, J=16.3, 5.9 Hz, 1H, G8Ha(b)), 3.33-3.29 (m, 2H, X9Hg), 3.21 (t, J=7.3 Hz, *n*) & 3.17 (t, J=7.3Hz, *m*) (2H, K2He, *m*+*n, m*/*n*=3/2), 3.14-3.05 (m, 3H, X9Ha+F6Hb(a)), 3.05-2.81 (m, 6H, F6Hb(b)+X3He+K7He+F5Hb(a)), 2.69-2.58 (m, 1H, F5Hb(b)), 2.38-2.12 (m, 8H, X4Hb+E1Hg+X4Ha+X3Ha), 1.92-1.80 (m, 1H, E1Hb(a)), 1.77-1.12 (m, 21H, E1Hb(b)+X9Hb+K2Hb(a)+K7Hb(a)+K7Hb(b)+K2Hb(b)+K7Hd +X3Hb+X3Hd+K2Hd+K2Hg+X3Hg, *m*+*n*), 1.40 -1.32 (m, 36H, tBu).

**$^{13}$C NMR (100 MHz, DMSO-*d$_6$*, δ)**: 172.55 (X4Cg(*nm*)), 172.23 (K2C(*n*)), 172.19 (K2C(*m*)), 172.12 (X3C(*n*)), 172.10 (X3C(*m*)), 171.91 (E1C), 171.69 (K7C), 171.63 (X4C), 171.53 (F6C), 171.44 (E1Cd), 171.36 (F5C), 168.64 (G8C), 157.13 (U(*m*)), 157.12 (U(*n*)), 155.59 (K7Boc), 141.16 (X10Cb(*m*)), 140.75 (X10Cb(*n*)), 137.98 (F6Cg), 137.93 (F5Cg), 133.43 (X10Ck(*n*)), 133.08 (X10Ck(*m*)), 130.59 (X10Cd(*n*)), 130.23 (X10Cd(*m*)), 129.09 (F6Cd), 129.04 (F5Cd), 128.16 (F6Ce), 128.03 (F5Ce), 127.21 (X10Ct(*m*)), 127.15 (X10Ce(*n*)), 126.86 (X10Ce(*m*)), 126.30 (X10Ct(*n*)+F6Ck), 126.23 (F5Ck), 126.06 (X10Cg(*m*)), 124.94 (X10Cg(*n*)), 80.57 (E1tBu), 80.40 (K2tBu(*m*)), 80.31 (K2tBu(*n*)), 79.75 (E1dtBu), 77.36 (K7BoctBu), 54.67 (F5Ca), 54.39 (F6Ca), 53.19 (K7Ca), 52.98 (K2Ca(*n*)), 52.86 (K2Ca(*m*)), 52.18 (E1Ca), 49.62 (X10Ca(*n*)), 48.30 (X9Cg), 47.09 (X10Ca(*m*)), 46.79 (K2Ce(*m*)), 45.22 (K2Ce(*n*)), 42.18 (G8Ca), 39.10 (K7Ce(*mn*), 38.66 (X3Ce(*m*)), 38.61 (X3Ce(*n*)), 36.88 (F5Cb), 36.71 (F6Cb), 35.86 (X9Ca), 32.32 (X3Ca(*n*)), 31.94 (X3Ca(*m*)), 31.82 (K2Cb), 31.20 (K7Cb), 30.91 (E1Cg), 30.74 (X4Ca), 30.58 (X4Cb), 29.28 (K7Cd), 29.08 (X3Cd(*m*)), 28.99 (X3Cd(*n*)), 28.32 (X9Cb), 28.28 (tBuK7), 27.74 (tBuE1), 27.64 (tBuK2+ K2Cd(*m*)), 27.62 (tBuE1d+E1Cb), 26.69 (K2Cd(*n*)), 26.31 (X3Cg(*m*)), 26.22 (X3Cg(*n*)), 24.74 (X3Cb(*m*)), 24.59 (X3Cb(*n*)), 22.64 (K7Cg), 22.44 (K2Cg(*n*)) 22.24 (K2Cg(*m*)).

**LCMS:** Purity – 100% (negative ions). Retention time – 12.551 min.

**ESI-MS**: $C_{75}H_{112}{}^{35}ClN_{13}O_{16}$: m/z calculated for $[M\text{-}H^+]^-$: 1484.80, found: 1484.85

**HRMS (m/z, ESI):** calculated for $C_{75}H_{112}{}^{35}ClN_{13}O_{16}$- $[M+H^+]^+$ 1486.8111, found: 1486.8180.

**Synthesis of compound 13.**

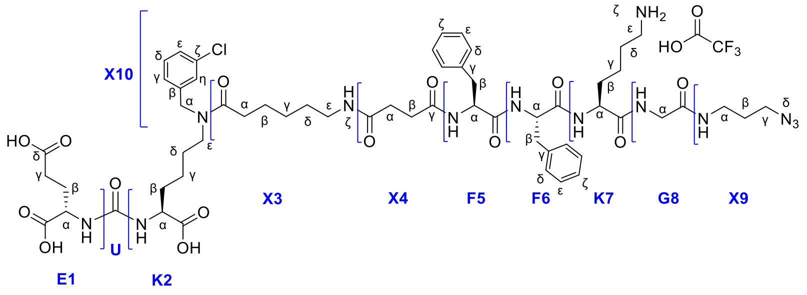


Compound **12** (1 equiv; 36 mg; 0.024 mmol) was dissolved in a mixture of DCM/TFA/TIPS/H2O (46.25%/46.25%/2.5%/5%; V/V, respectively, total volume - 2 mL). Then the reaction was stirred for 3 hours, subsequently the solvent was removed under reduced pressure and re-evaporated with DCM three times (5 ml). The product was precipitated with $Et_2O$ and washed twice with $Et_2O$ (1 mL). The residue was purified by column chromatography (Puriflash on a PF-15C18AQ-F0004 column (15 μm, 6 g), eluent: $H_2O$×TFA(0.1%) (80%)/MeCN (20%) => $H_2O$×TFA(0.1%) (0%)/MeCN (100%) for 15 minutes, followed by MeCN (100%) for 5 minutes. Thus, compound **13** was obtained as a salt with ×1 TFA (23 mg, 79% yield).

**$^1$H NMR (400 MHz, DMSO-*d*$_6$, δ):** 12.48 (br.s., 3H, COOH), 8.24 (d, J=7.7 Hz, 1H, F5NH), 8.19 (d, J=7.6 Hz, 1H, F6NH), 8.05-7.94 (m, 2H, G8NH+K7NH), 7.94-7.86 (m, 1H, X3NHk(*m*+*n*)), 7.80 (t, J=5.5 Hz, X9NH), 7.72-7.56 (br.s., 3H, K7NHk$_3^+$ 7.42-7.09 (m, 14H, X10Hd*n*+X10He*n*+X10Hd*m* +X10He*m*+F6He+F6Hd+X10Ht*mn* +F5He+ F6Hk+F5Hk+F5Hd+X10Hg*mn*), 6.41-6.23 (m, 2H, K2NH+E1NH, *m*+*n*), 4.60-4.42 (m, 3HF6Ha+X10Ha(*n*+*m*)), 4.41-4.32 (m, 1H, F5Ha), 4.22-4.13 (m, 1H, K7Ha), 4.13-3.97 (m, 2H, E1Ha+K2Ha), 3.75-3.58 (m, 2H, G8Ha), 3.36-3.29 (m, 2H, X9Hg), 3.24-2.85 (m, 9H, K2He+X9Ha+F6Hb(a))+F6Hb(b)+X3He+F5Hb(a)), 2.80-2.70 (m, 2H, K7He), 2.70-2.58 (m, 1H, F5Hb(b)), 2.40-2.10 (m, 8H, X4Hb+E1Hg+X4Ha+X3Ha), 1.98-1.83 (m, 1H, E1Hb(a)), 1.77-1.12 (m, 21H, E1Hb(b)+X9Hb+K2Hb(a)+K7Hb(a)+K7Hb(b)+K2Hb(b)+K7Hd +X3Hb+X3Hd+K2Hd+K2Hg+X3Hg+K7Hg, *m*+*n*).

**$^{13}$C NMR (100 MHz, DMSO-*d*$_6$, δ)**: 174.55 (K2C(*n*)), 174.51 (K2C(*m*)), 174.21 (E1C(*nm*)), 173.80 (E1Cδ), 172.48 (X4Cγ(*n*)), 172.45 (X4Cγ(*m*)), 172.23 (X3C(*mn*)), 171.65 (K7C), 171.60 (F5C), 171.53 (X4C), 171.36 (F6C), 168.70 (G8C), 157.34 (U), 141.23 (X10Cβ(*m*)), 140.83 (X10Cβ(*n*)), 138.00 (F6Cγ), 137.88 (F5Cγ), 133.44 (X10Cζ(*n*)), 133.09 (X10Cζ(*m*)), 130.64 (X10Cδ(*n*)), 130.27 (X10Cδ(*m*)), 129.15 (F6Cδ), 129.05 (F5Cδ), 128.20 (F6Cε), 128.09 (F5Cε), 127.21 (X10Cη(*m*)), 127.16 (X10Cε(*n*)), 126.88 (X10Cε(*m*)), 126.37 (F6Cζ), 126.30 (X10Cη(*n*)+F5Cζ), 126.11 (X10Cγ(*m*)), 124.99 (X10Cγ(*n*)),, 54.61 (F5Cα), 54.37 (F6Cα), 52.84 (K7Cα), 52.28 (K2Cα(*n*)), 52.18 (K2Cα(*m*)), 51.71 (E1Cα), 49.67 (X10Cα(*n*)),

48.34 (X9Cγ), 47.21 (X10Cα(*m*)), 46.94 (K2Cε(*m*)), 45.37 (K2Cε(*n*)), 42.15 (G8Cα), 38.73 (K7Cε), 38.69 (X3Cε(*m*)), 38.61 (X3Cε(*n*)), 36.92 (F6Cβ), 36.73 (F5Cβ), 35.92 (X9Cα), 32.34 (X3Cα(*n*)), 31.92 (X3Cα(*m*)),, 31.83 (K2Cβ), 30.92 (K7Cβ), 30.82 (X4Cα), 30.63 (X4Cβ), 29.95 (E1Cγ),, 29.11 (X3Cδ(*m*)),, 29.00 (X3Cδ(*n*)), 28.34 (X9Cβ), 27.81 (K2Cδ(*m*)), 27.58 (E1Cβ), 26.76 (K2Cδ(*n*)), 26.66 (K7Cδ), 26.30 (X3Cγ(*m*)), 26.22 (X3Cγ(*n*)), 24.76 (X3Cβ(*m*)), 24.61 (X3Cβ(*n*)), 22.54 (K2Cγ(*n*)), 22.35 (K2Cγ(*m*)), 22.13 (K7Cγ).

**LCMS:** Purity – 100% (negative ions). Retention time – 11.463 min.

**ESI-MS**: $C_{58}H_{80}{}^{35}ClN_{13}O_{14}$: m/z calculated for $[M\text{-}H^+]^-$: 1216.56, found: 1216.55

**HRMS (m/z, ESI):** calculated for $C_{58}H_{80}{}^{35}ClN_{13}O_{14}$ - $[M+H^+]^+$ 1218.5709, found: 1218.5700.

**Synthesis of compound 14.**

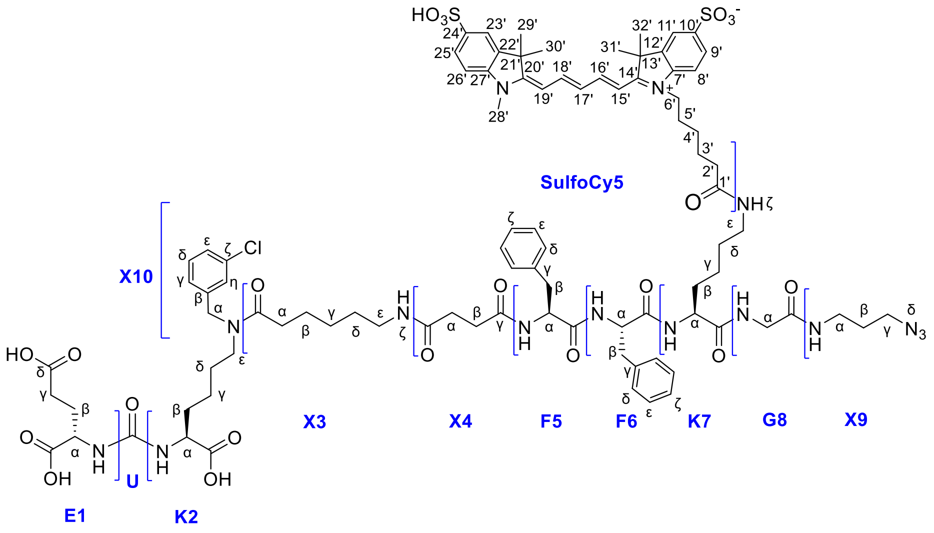


Compound **13** (1 equiv; 15 mg; 11.3 µmol) and DIPEA (8 equiv; 16 µl; 90 µmol) were dissolved in DMF (2 mL), and the reaction was purged with argon. Sulfo-Cy5 NHS-ester (1.05 equiv; 9 mg; 11.8 µmol) was added to the mixture. The reaction was stirred for 12 hours, after which the solvent was evaporated under reduced pressure. The residue was purified by column chromatography (Puriflash on a PF-15C18AQ-F0012 column (15 µm, 20 g), eluent: $H_2O$ (90%)/MeCN (10%) => $H_2O$ (0%)/MeCN (100%). Within 20 min after MeCN (100%) for 5 min, compound 14 was obtained as a blue powder (17.7 mg, 86% yield).

**$^1$H NMR (400 MHz, DMSO-*d₆*, δ)**: 12.62 (br.s., COOH), 8.35 (t, J = 12.9 Hz, 2H, 18'+16'), 8.30-8.23 (m, 1H, F5NH), 8.23-8.16 (m, 1H, F6NH), 8.05-7.97 (m, 1H, G8NH), 7.97-7.86 (m, 2H, K7NH+X3NHk(*m*+*n*)), 7.85-7.78 (m, 2H, 11'+23'), 7.78-7.68 (m, 2H, K7NHk+X9NH), 7.67-7.59 (m, 2H, 9'+25'), 7.42-7.09 (m, 16H, X10Hd*n*+X10He*n*+X10Hd*m* +8'+26'+X10He*m*+F6He+F6Hd+X10Ht*mn*+F5He+ F6Hk+F5Hk+F5Hd+X10Hg*mn*), 6.55 (t, J = 12.2 Hz, 1H, 17'), 6.40-6.19 (m, 4H, K2NH+19'+E1NH+15', *m*+*n*), 4.60-4.41 (m, 3H,

F6Ha+X10Ha(*n*+*m*)), 4.40-4.30 (m, 1H, F5Ha), 4.17-3.96 (m, 7H, K7Ha+E1Ha+6’+K2Ha), 3.74-3.57 (m, 2H, G8Ha), 3.31 (t, J = 6.8 Hz, 2H, X9Hg), 3.23-2.85 (m, 11H, K2He*mn*+F6Hb(a)+X9Ha+X3He(*mn*)+K7He+F6Hb(b)+F5Hb(a)), 2.70-2.59 (m, 1H, F5Hb(b)), 2.38-2.10 (m, 8H, X4Hb+E1Hg+X4Ha+X3Ha), 2.02 (t, J = 6.7 Hz, 2H, 2’), 1.95-1.85 (m, 1H, E1Hb(a)), 1.77-1.68 (m, 1H, E1Hb(b)), 1.68 (S, 12H, 29’+30’+31’+32’), 1.67-1.12 (m, 27H, E1Hb(b) + X9Hb + K2Hb(a) + K7Hb(a) + K7Hb(b) + 5’+ K2Hb(b) + K7Hd + X3Hb + 3’ + X3Hd + K2Hd + 4’+ K2Hg + X3Hg + K7Hg, *m*+*n*).

**LCMS:** Purity – 100% (negative ions). Retention time – 10.449 min.

**ESI-MS**: $C_{90}H_{116}ClN_{15}O_{21}S_2$: m/z calculated for $[M-2H^+]^{2-}$: 920.37, found: 920.6

**HRMS (m/z, ESI):** calculated for $C_{90}H_{116}{}^{35}ClN_{15}O_{21}S_2$ - $[M-H^+]^-$ 1840.7527, found: 1840.7459.

# Spectra

Compound **6**

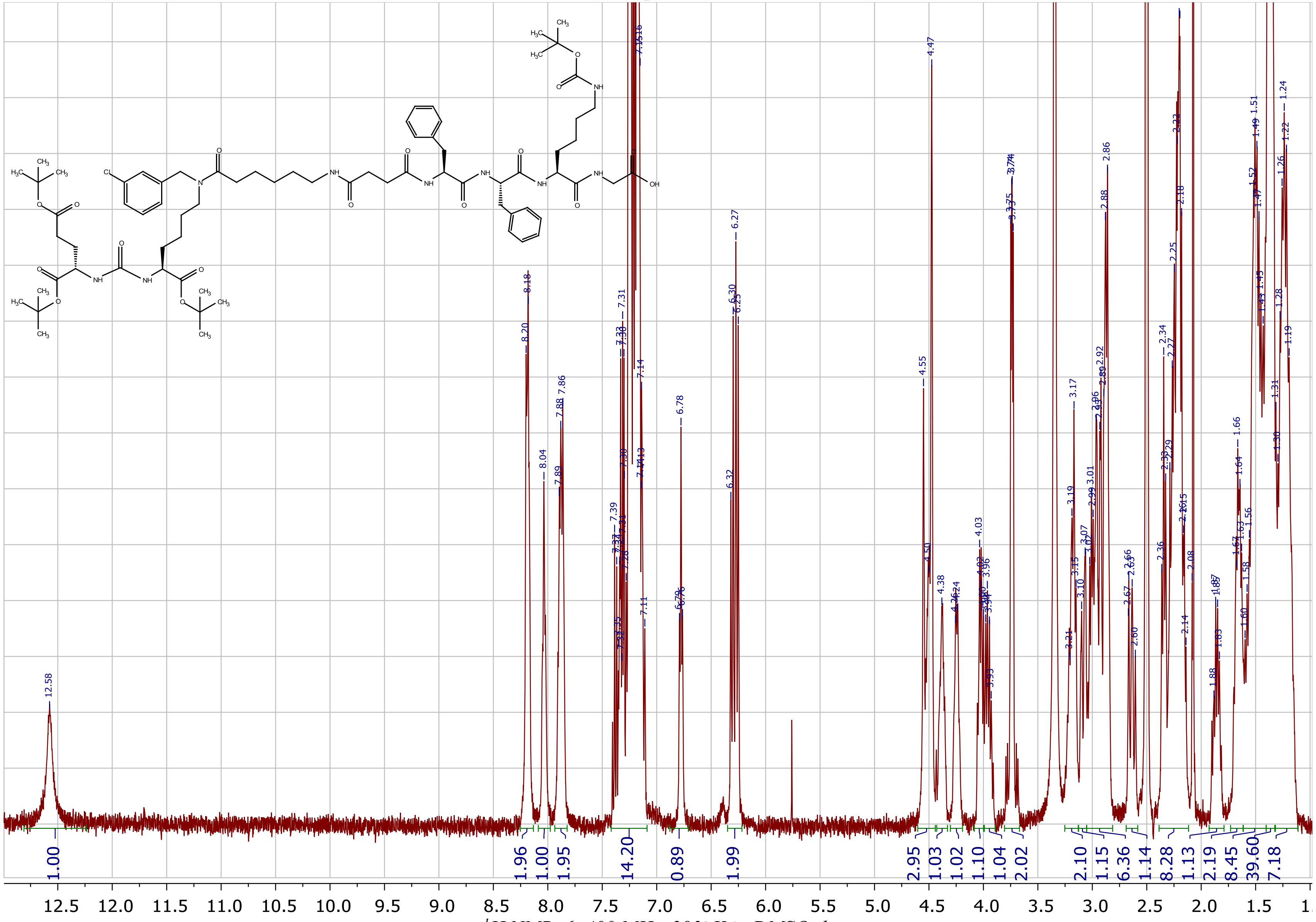


$^{1}H$ *NMR* ***6****, 400 MHz, 293º K in DMSO-$d_6$*

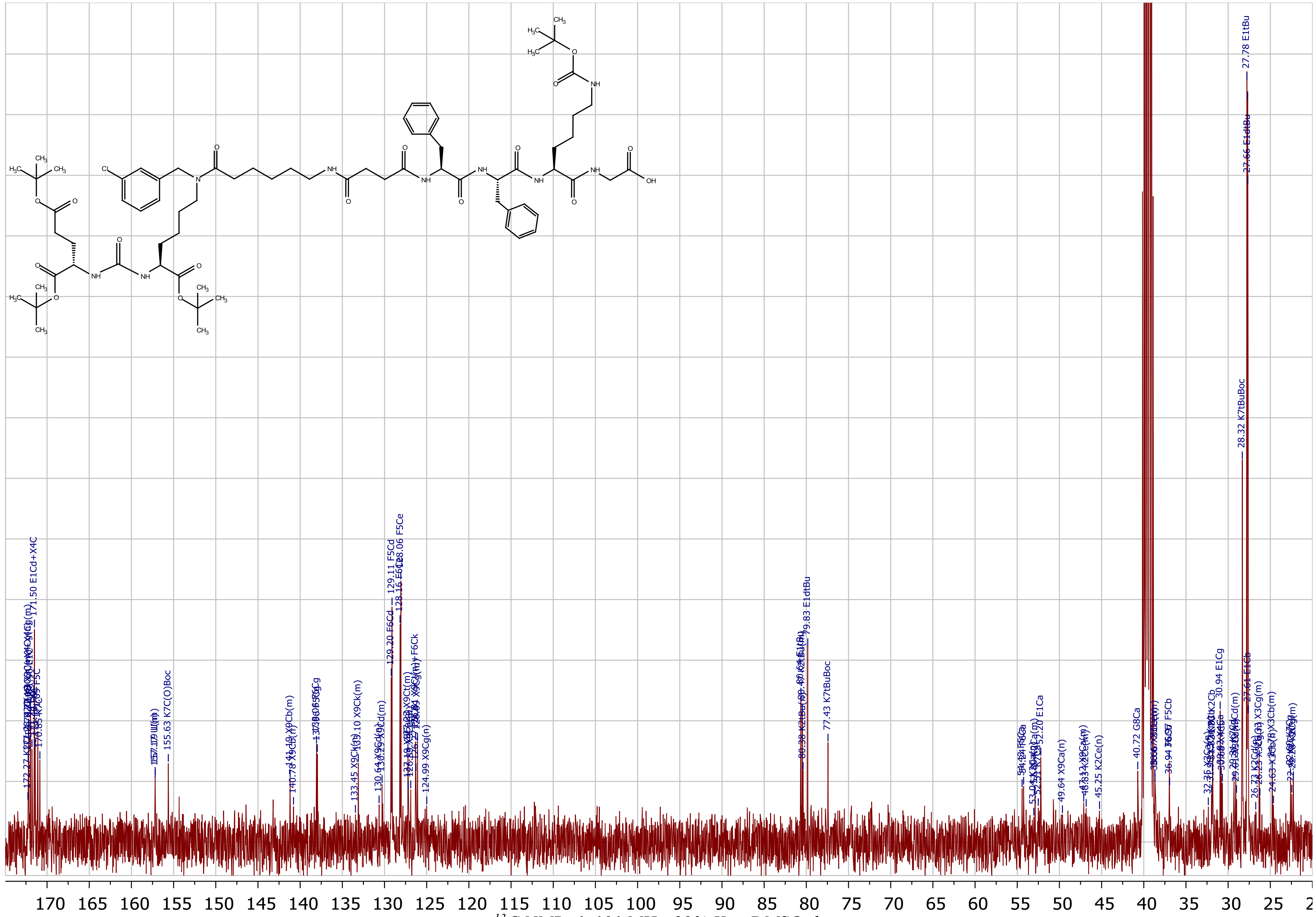

$^{13}C$ NMR **6**, 101 MHz, 293° K in DMSO-$d_6$

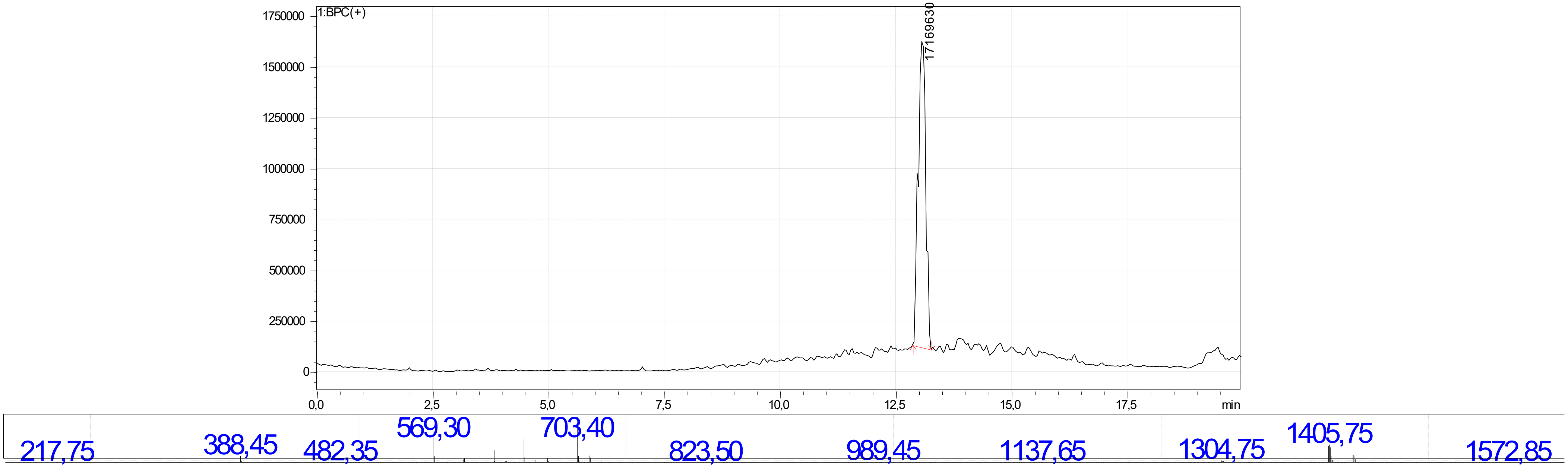


*HPLC-MS (ESI)* ***6***.

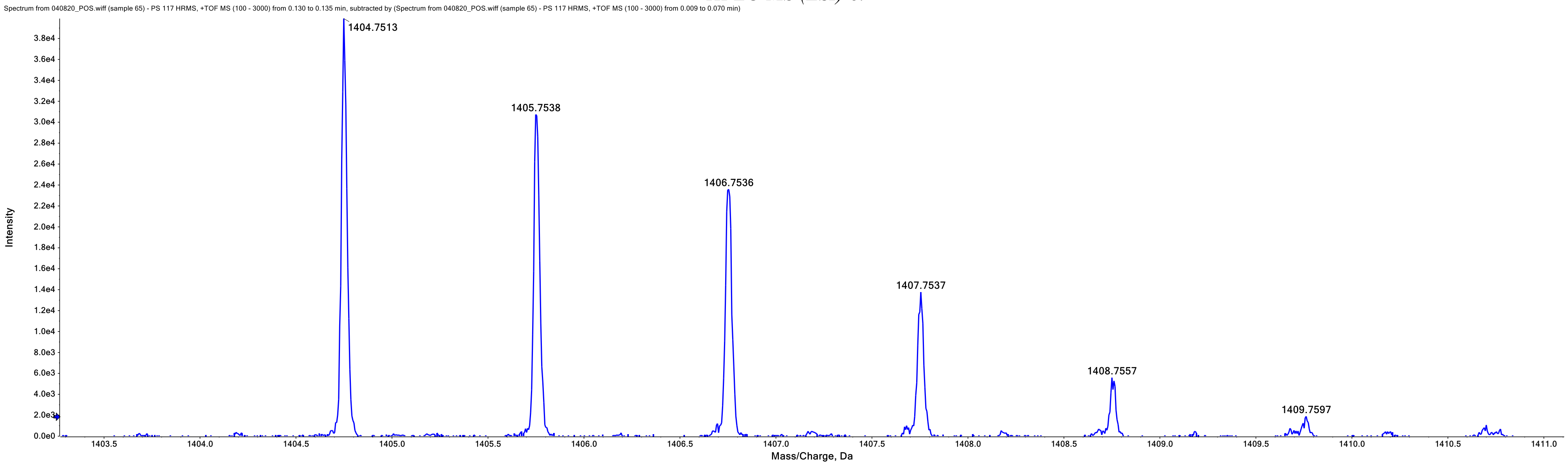


*HRMS* ***6***

Compound **7**

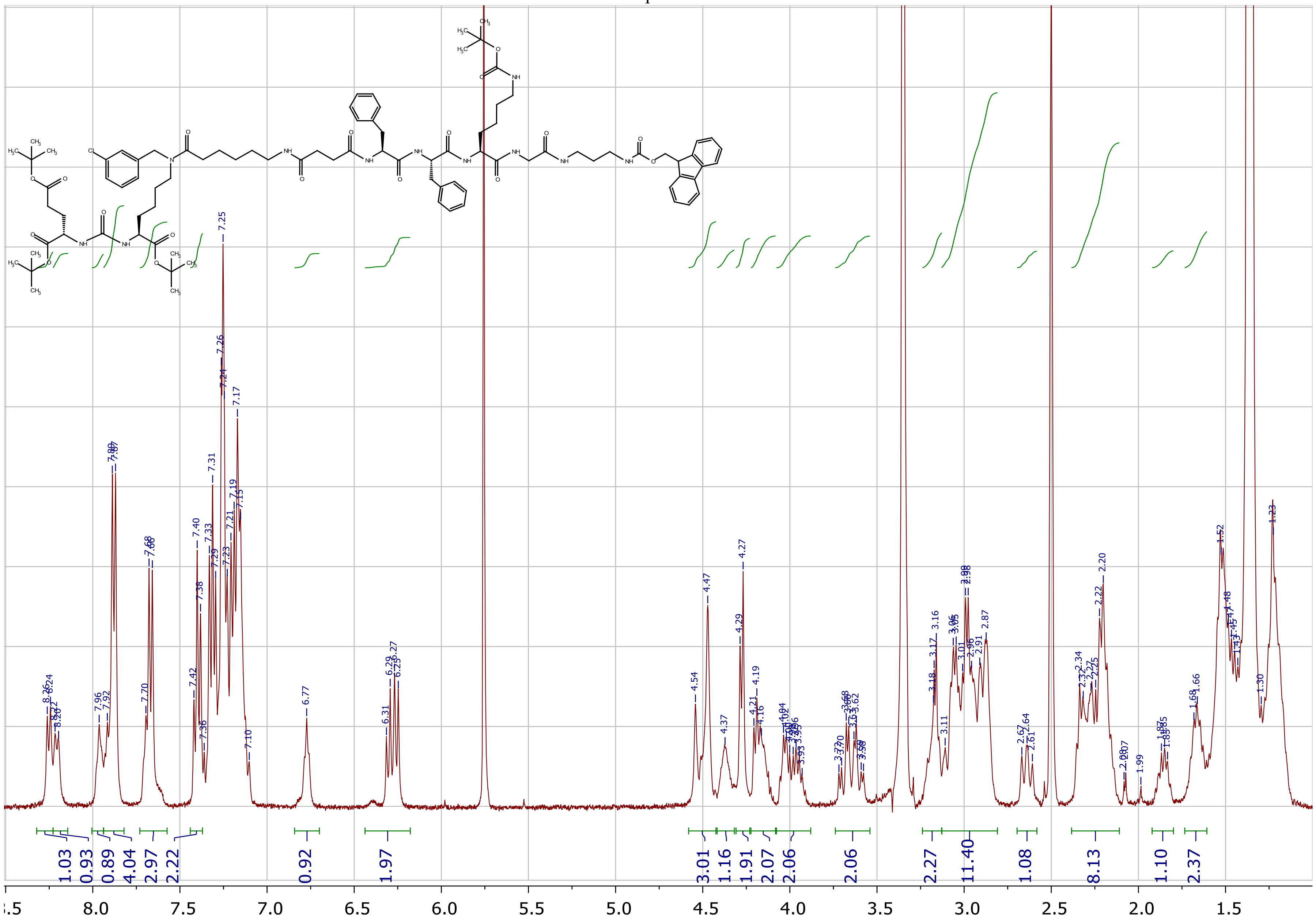

$^{1}H$ NMR **7**, 400 MHz, 293° K in DMSO-$d_6$

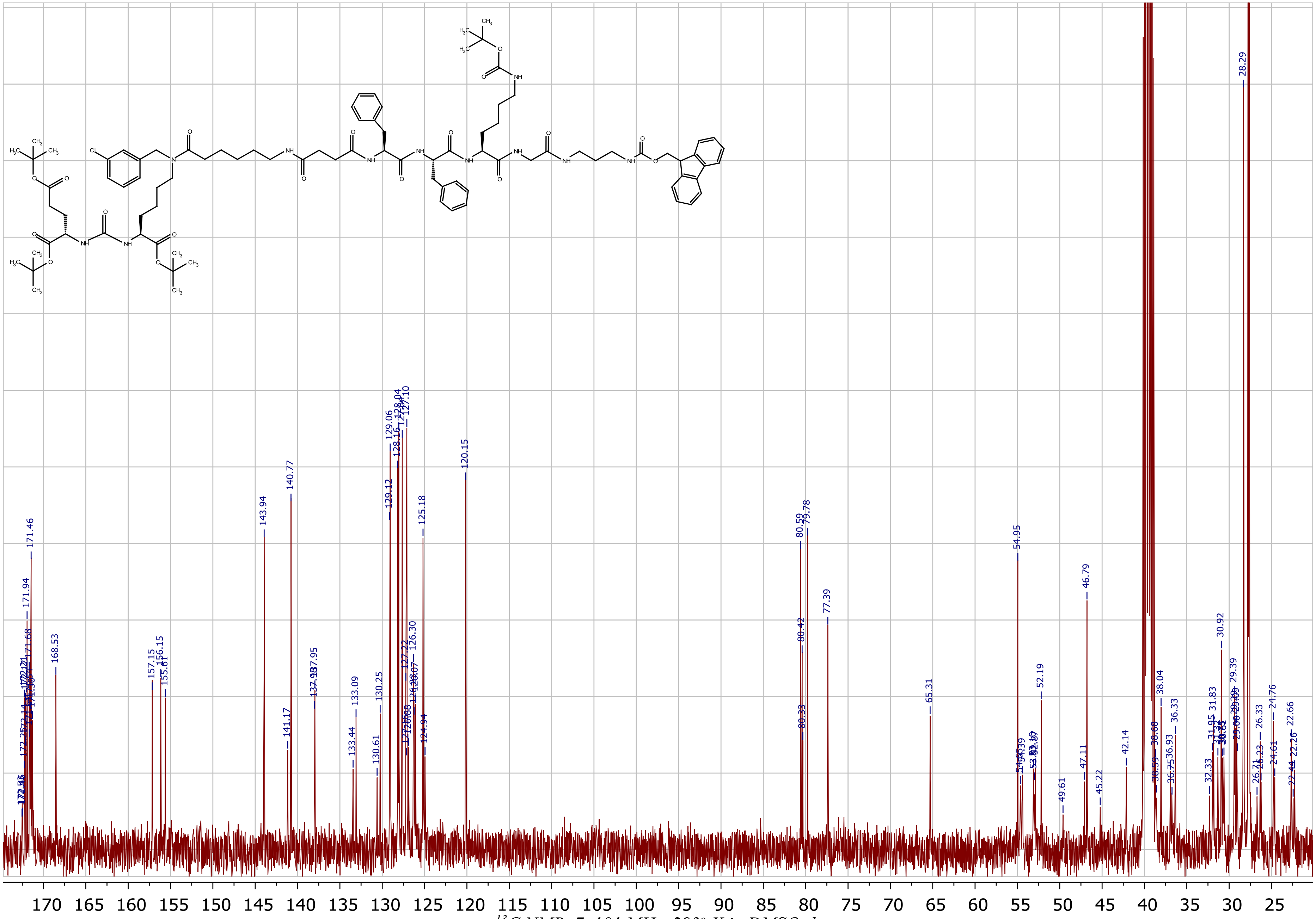

$^{13}$C NMR 7, 101 MHz, 293° K in DMSO-$d_6$

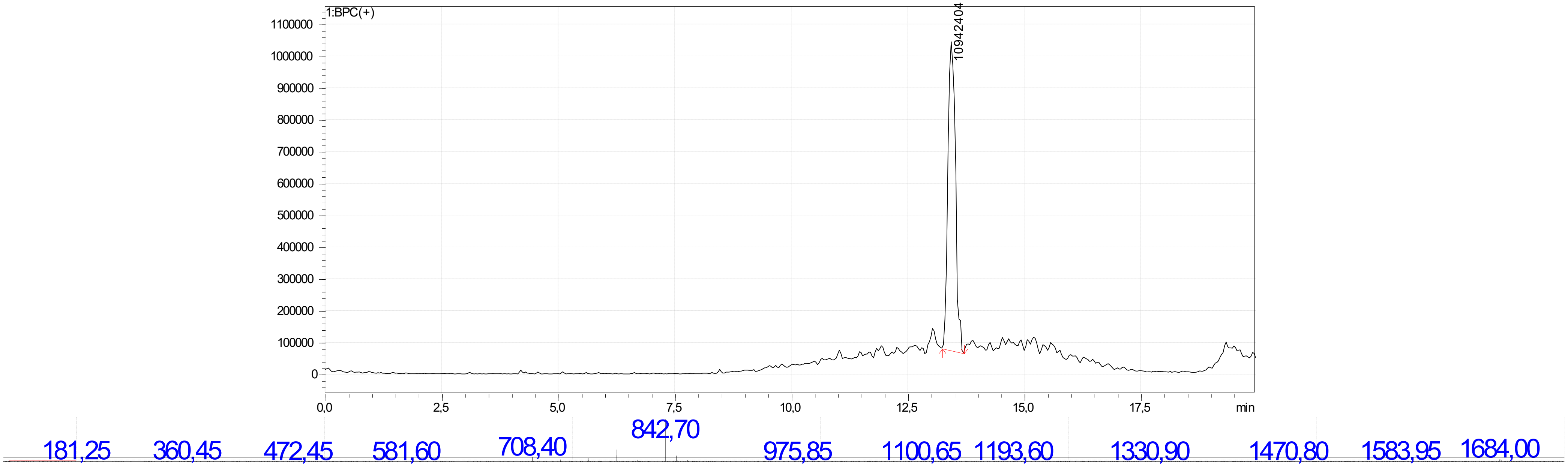


*HPLC-MS (ESI) 7.*

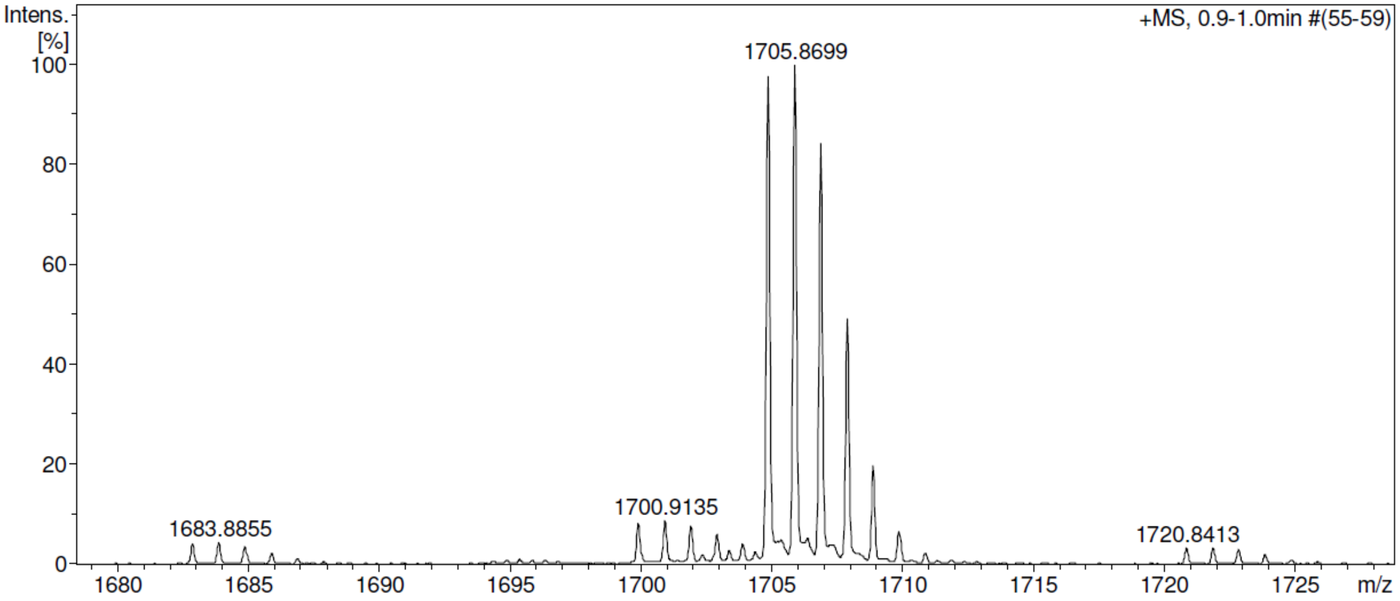


*HRMS 7.*

Compound **8**

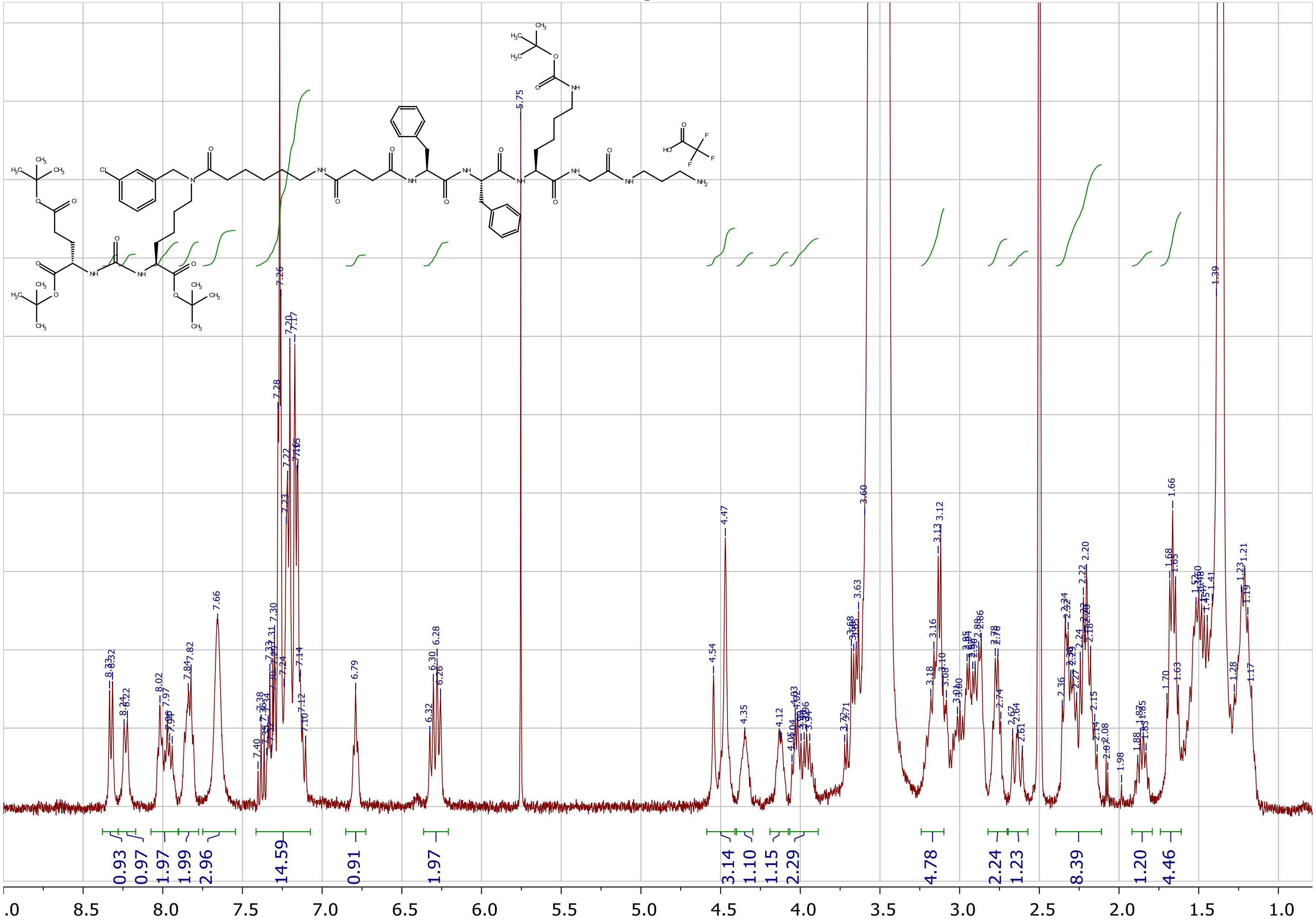


*$^1$H NMR* ***8****, 400 MHz, 293° K in DMSO-d$_6$*

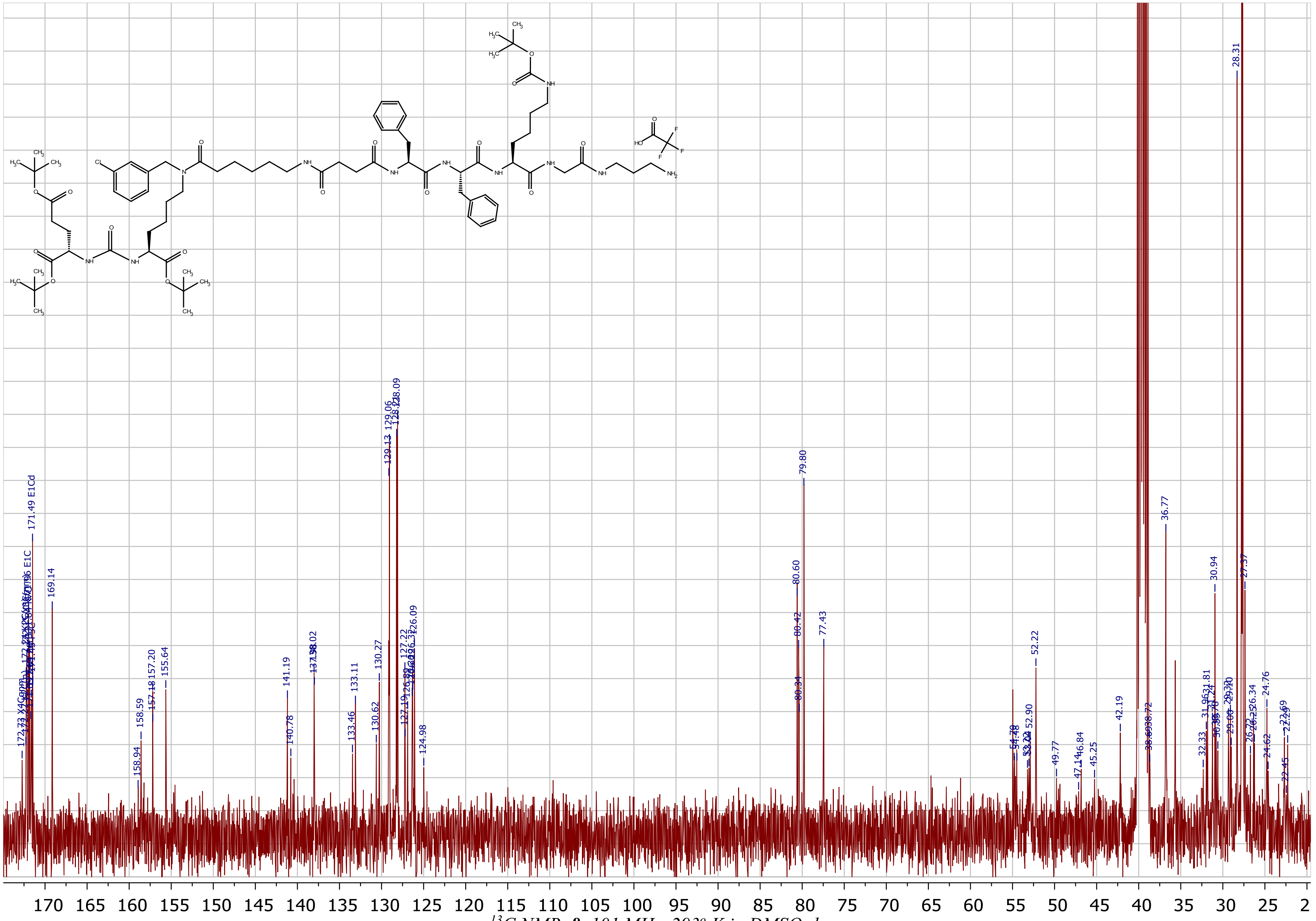

$^{13}C$ NMR **8**, 101 MHz, 293° K in DMSO-$d_6$

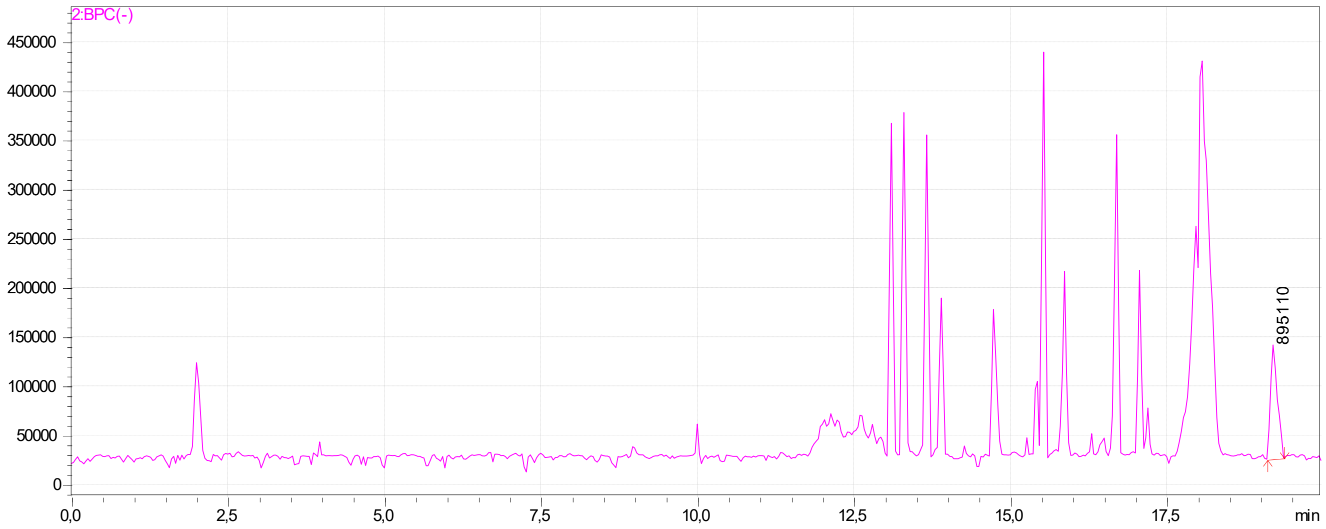


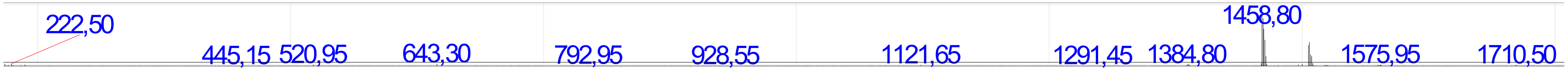


*HPLC-MS (ESI)* ***8***.

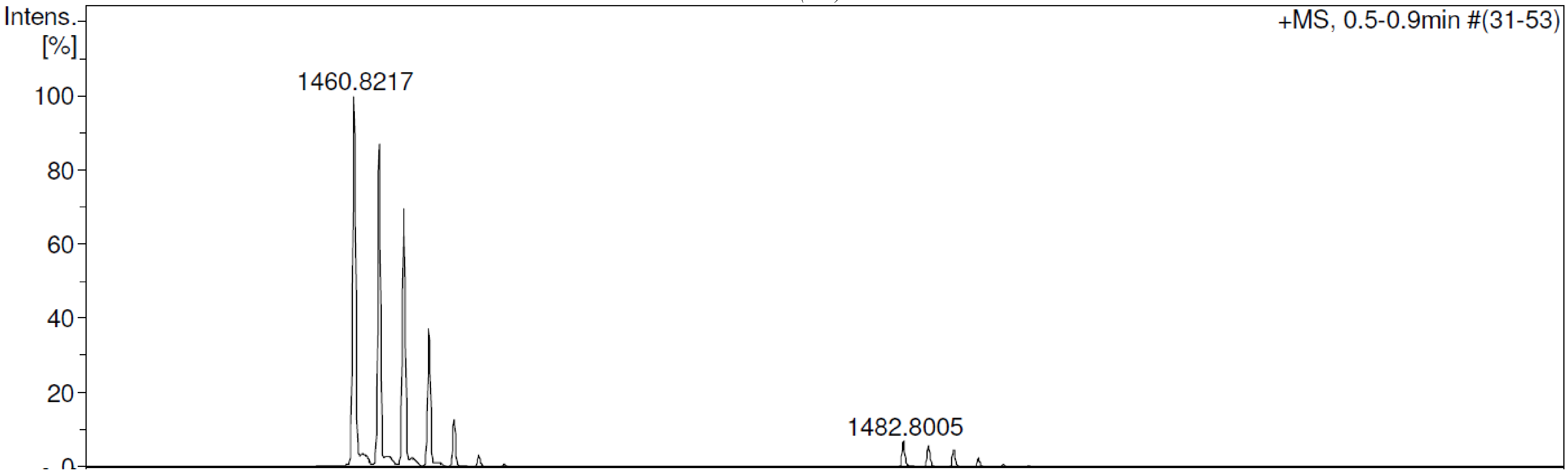


*HRMS* ***8***.

Compound **9**

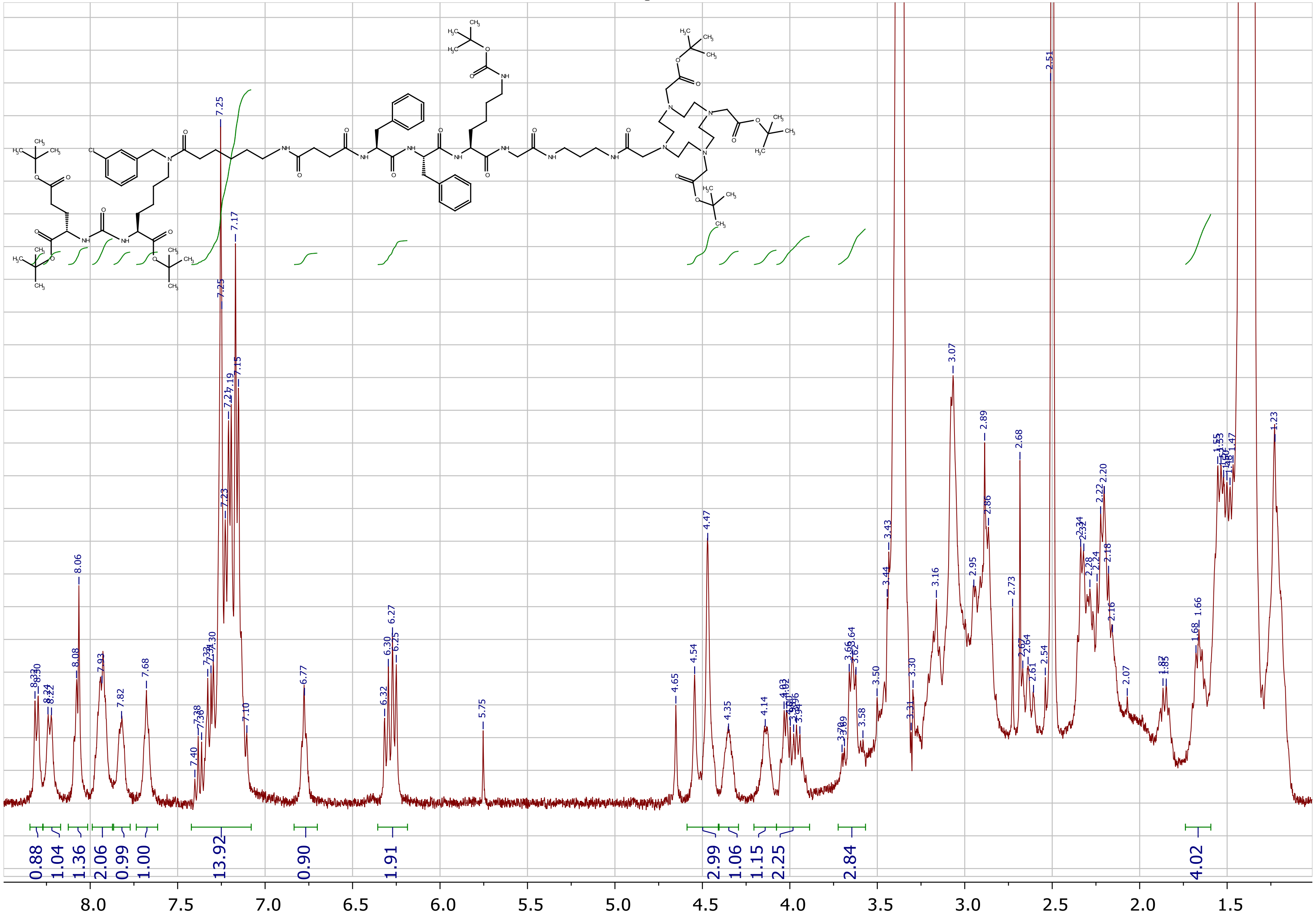

$^{1}H$ NMR **9**, 400 MHz, 293° K in DMSO-$d_6$

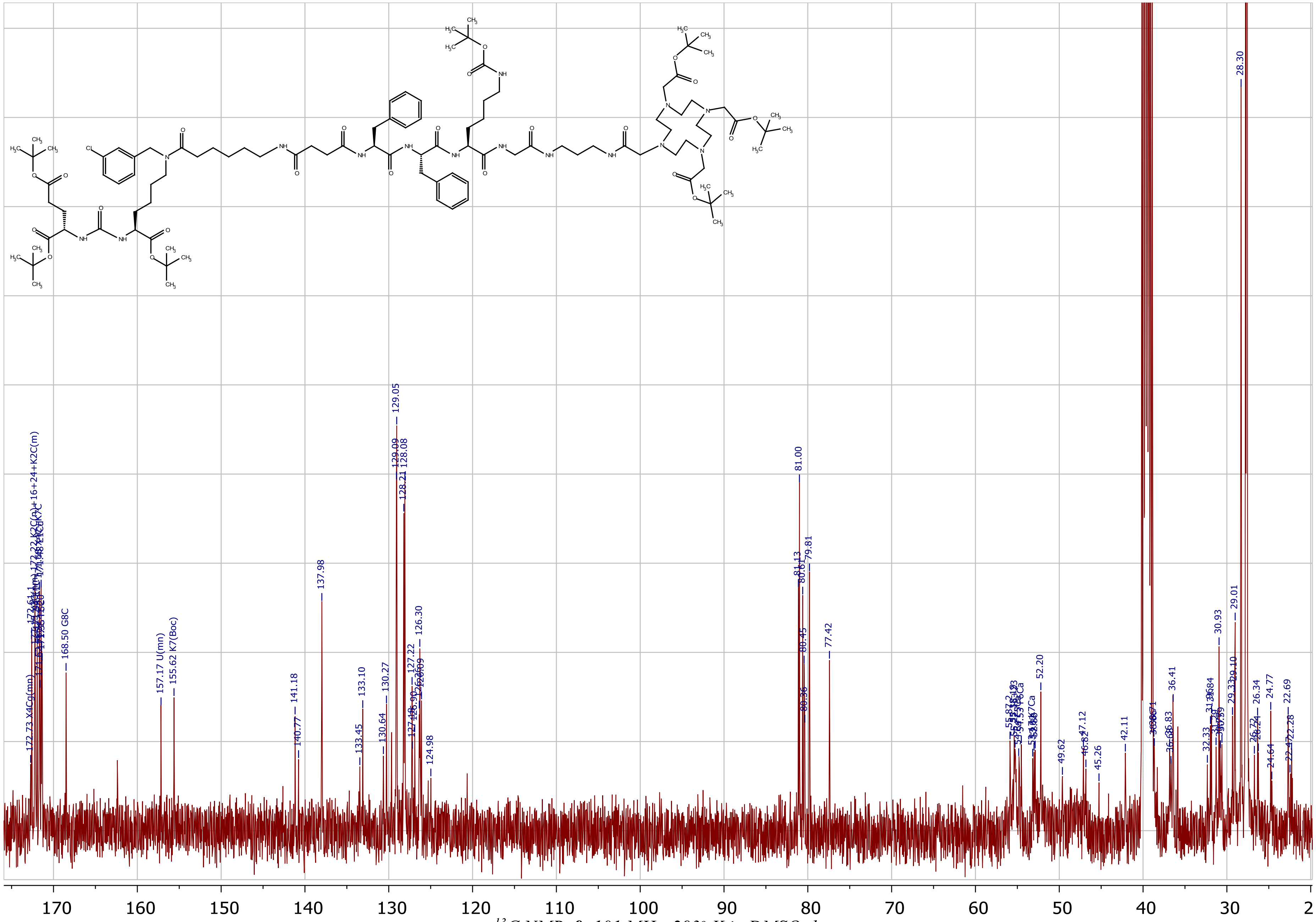


$^{13}$C NMR **9**, 101 MHz, 293° K in DMSO-$d_6$

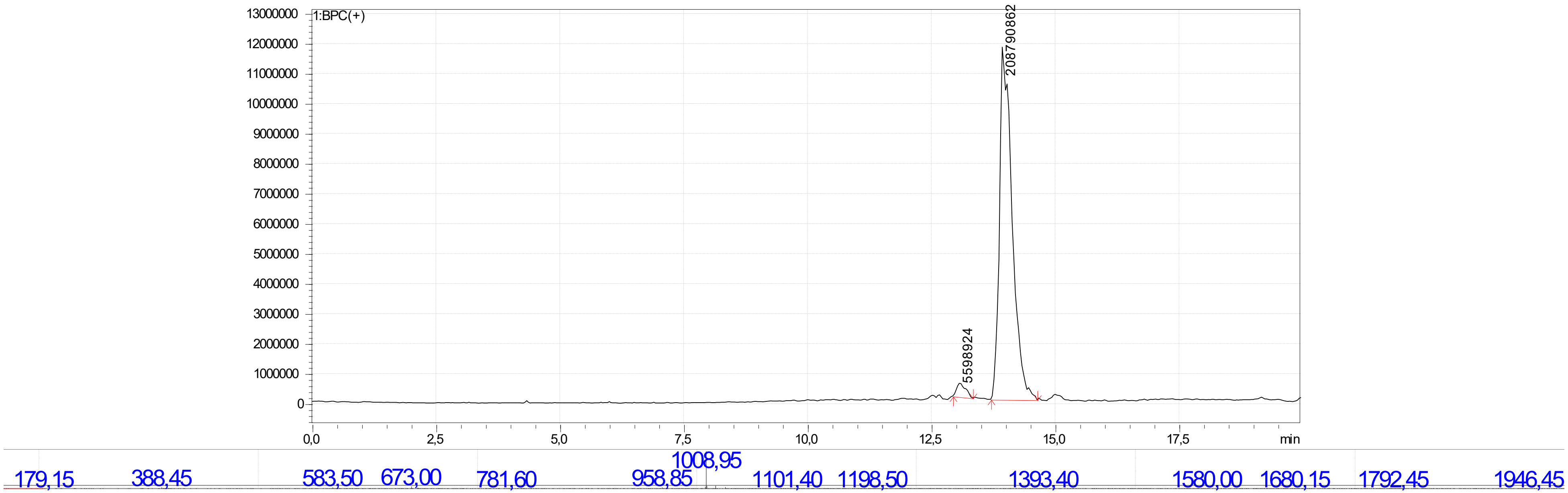


*HPLC-MS (ESI)* ***9***.

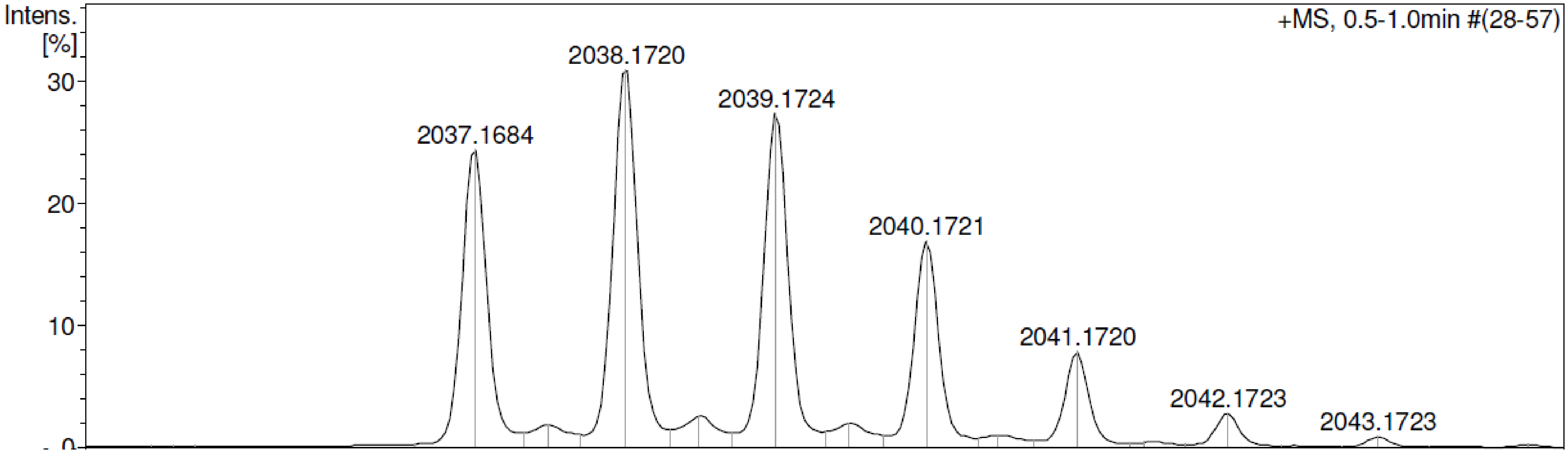


*HRMS* ***9***.

Compound **10**

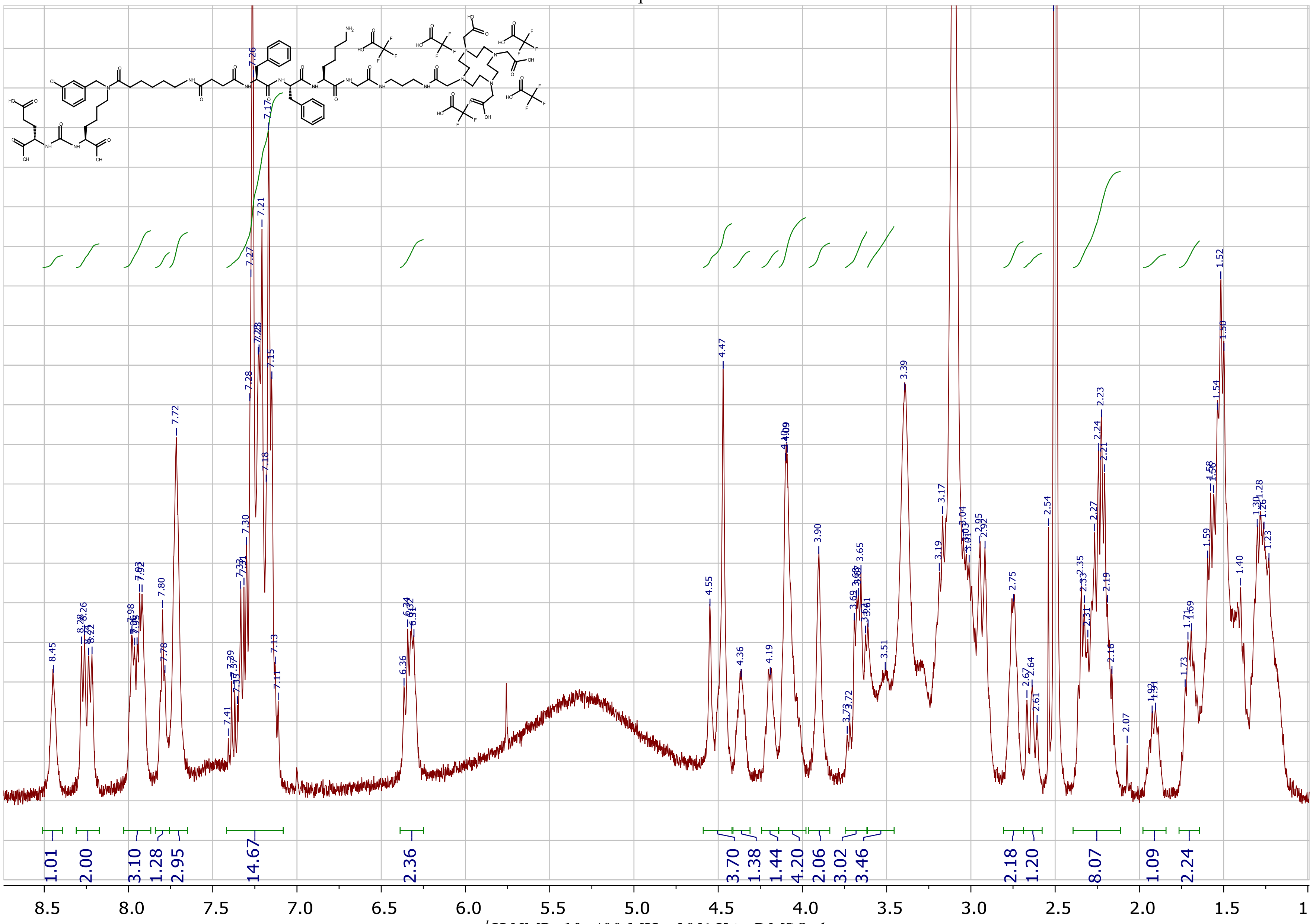

$^{1}$H NMR **10**, 400 MHz, 293° K in DMSO-$d_6$

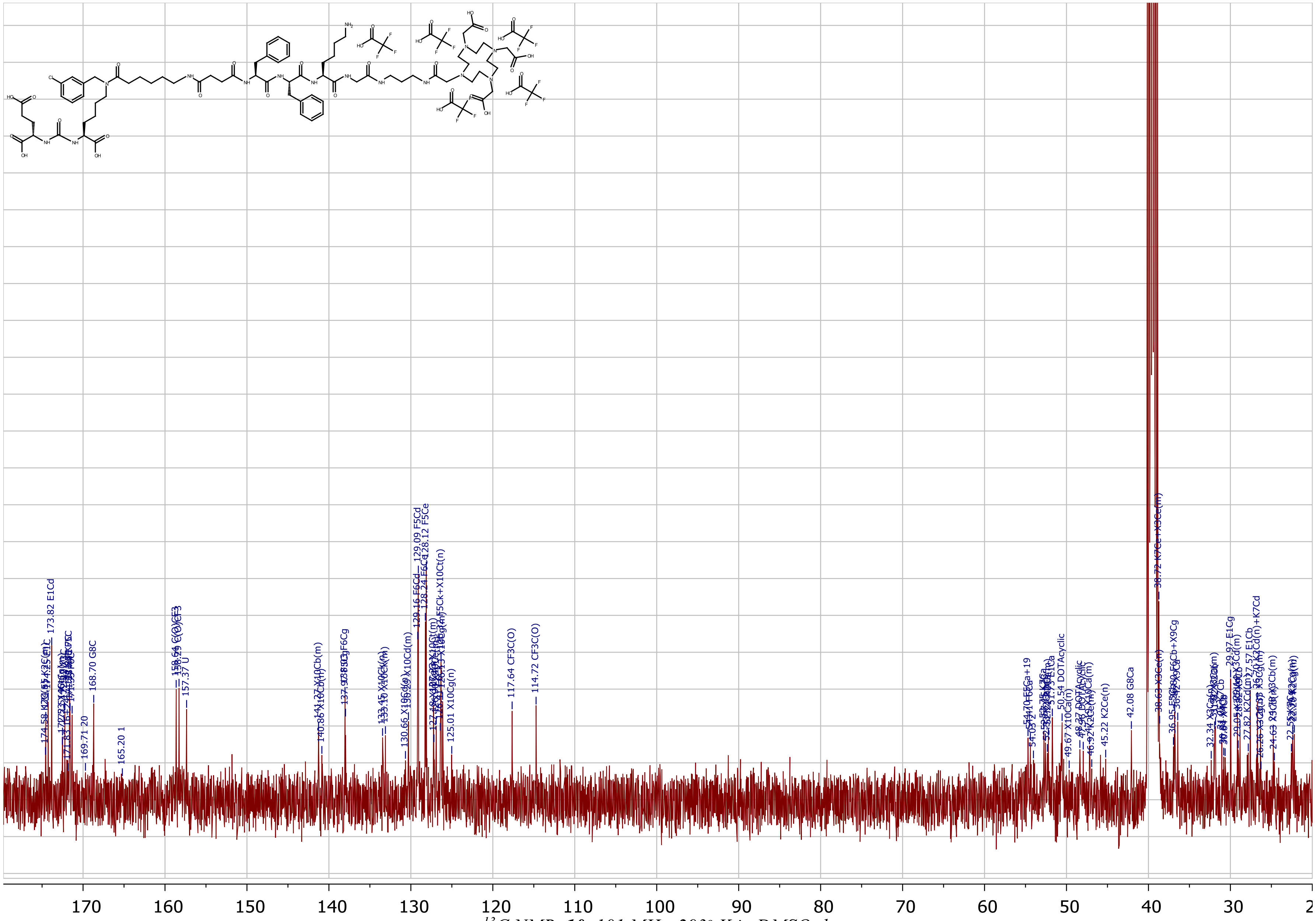


$^{13}C$ NMR **10**, 101 MHz, 293° K in DMSO-$d_6$

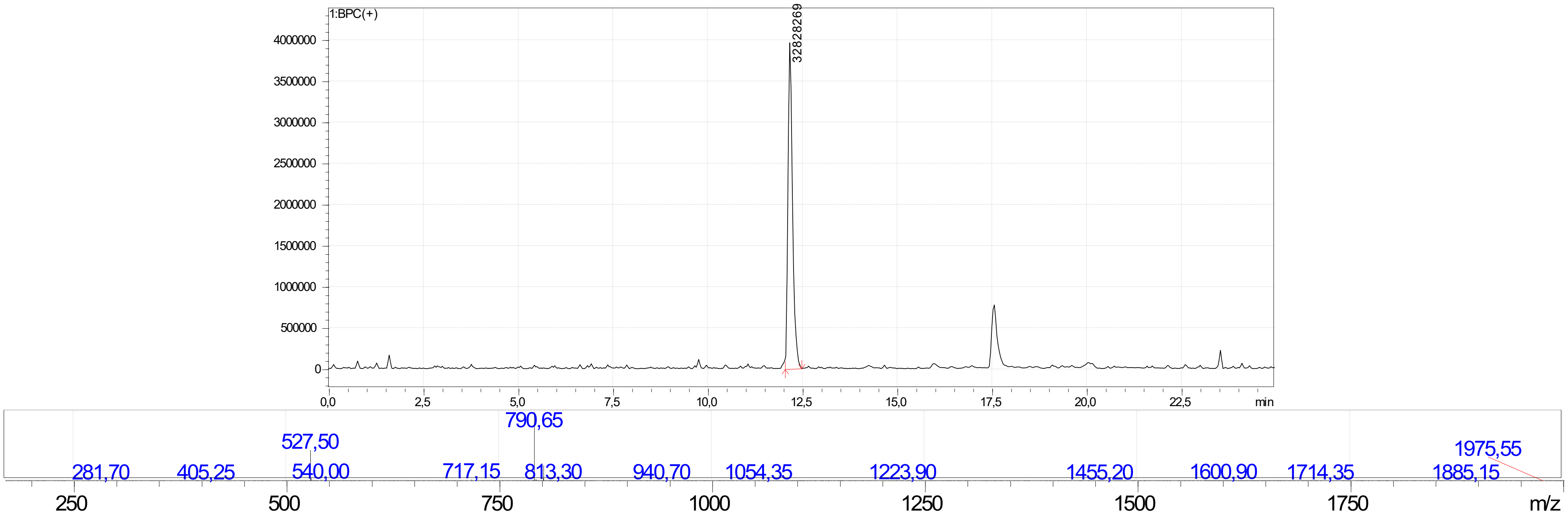


*HPLC-MS (ESI)* ***10***.

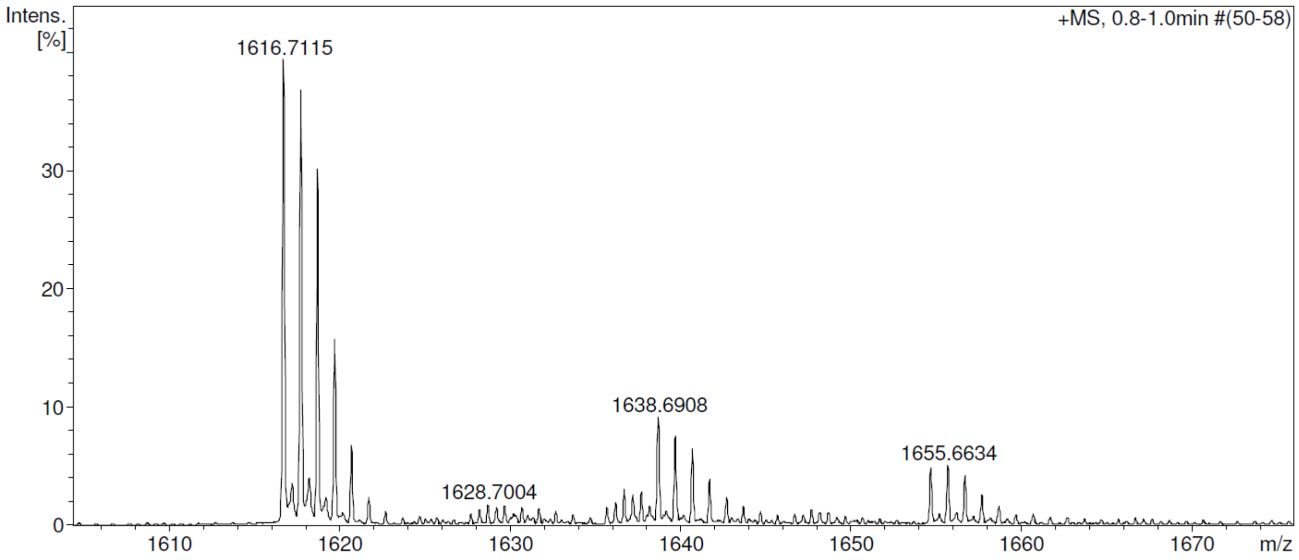


*HRMS* ***10***.

Compound **11**

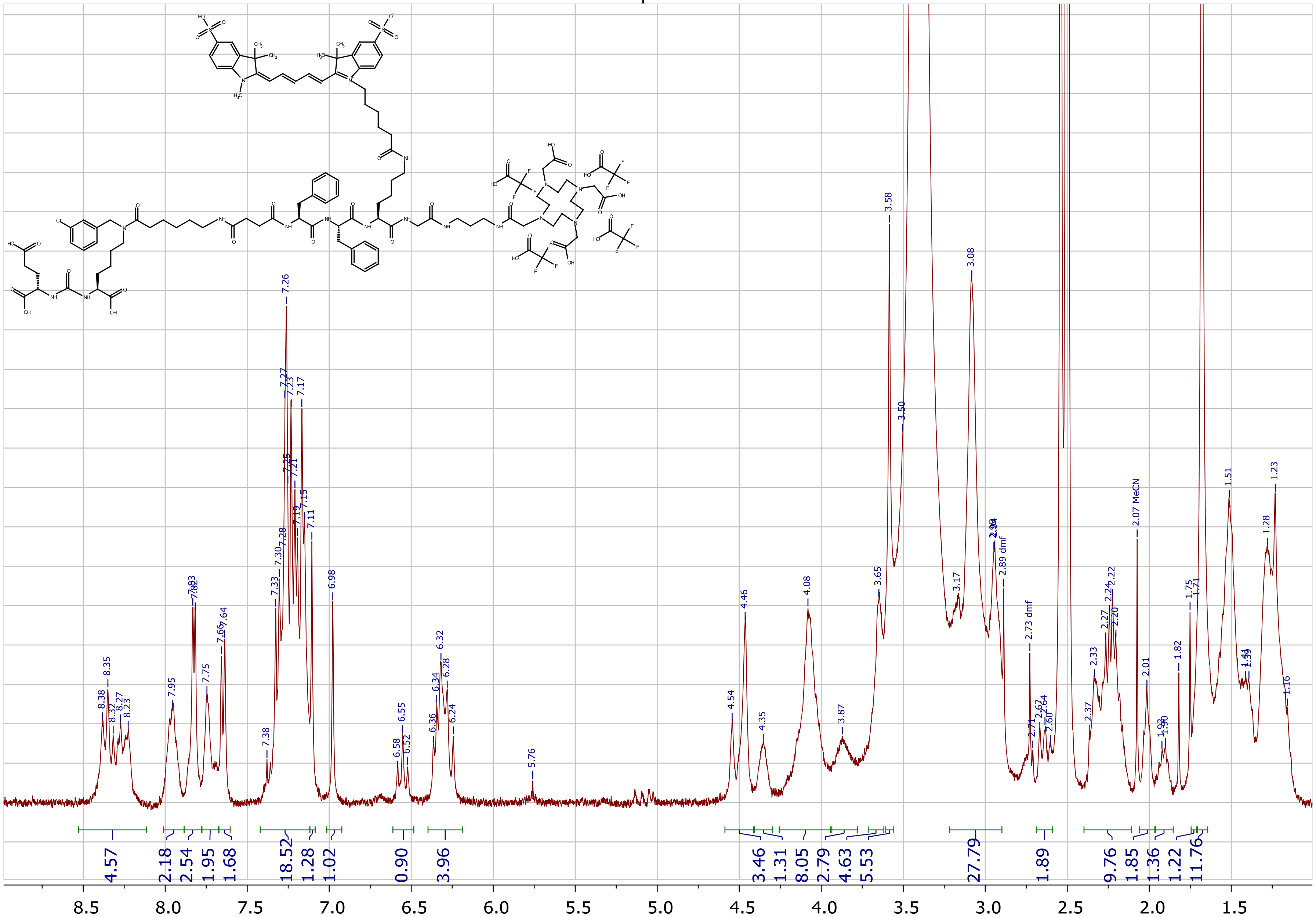


*$^1H$ NMR* ***11****, 400 MHz, 293º K in DMSO-$d_6$*

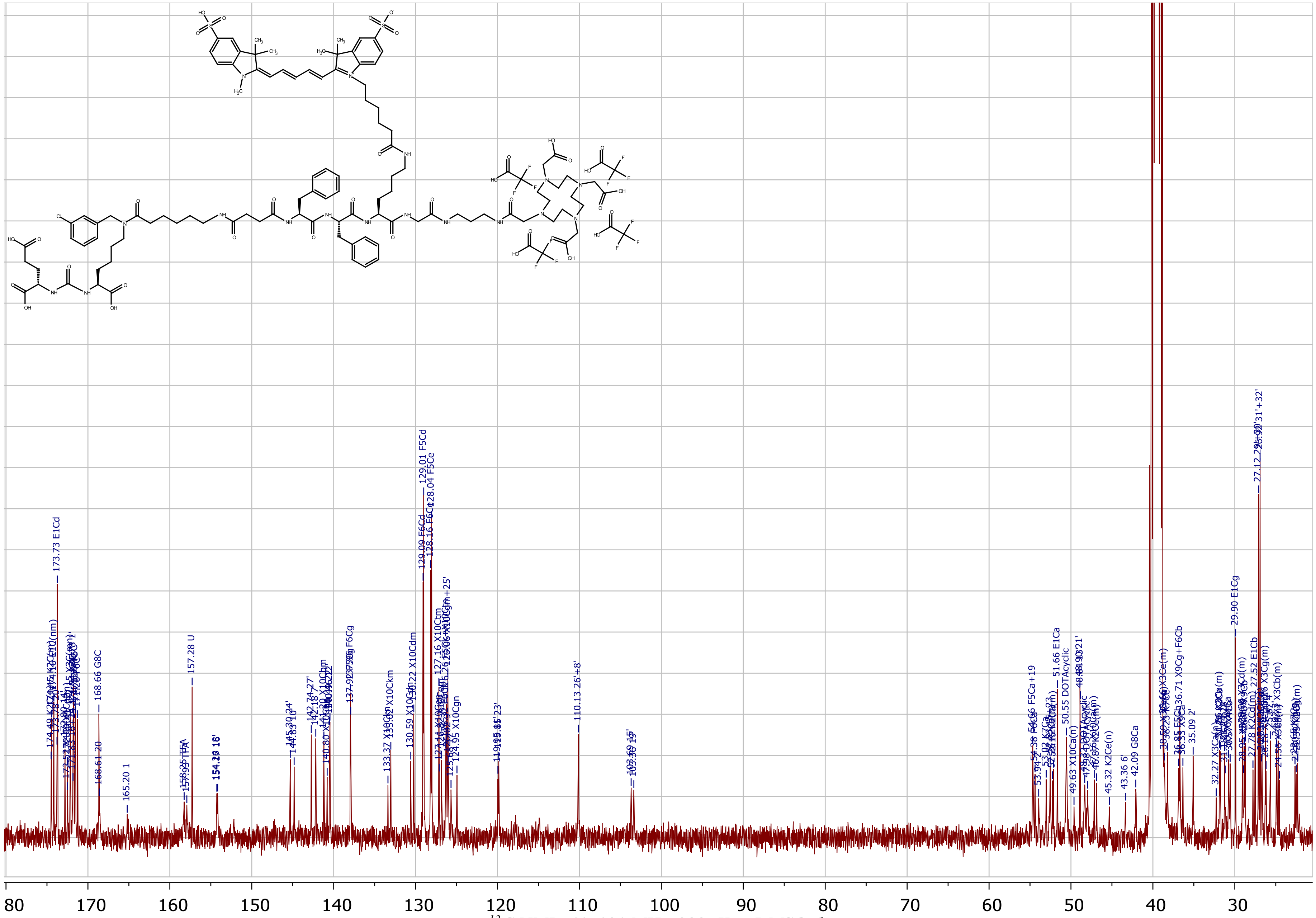

$^{13}$C NMR **11**, 101 MHz, 293$^{o}$ K in DMSO-$d_6$

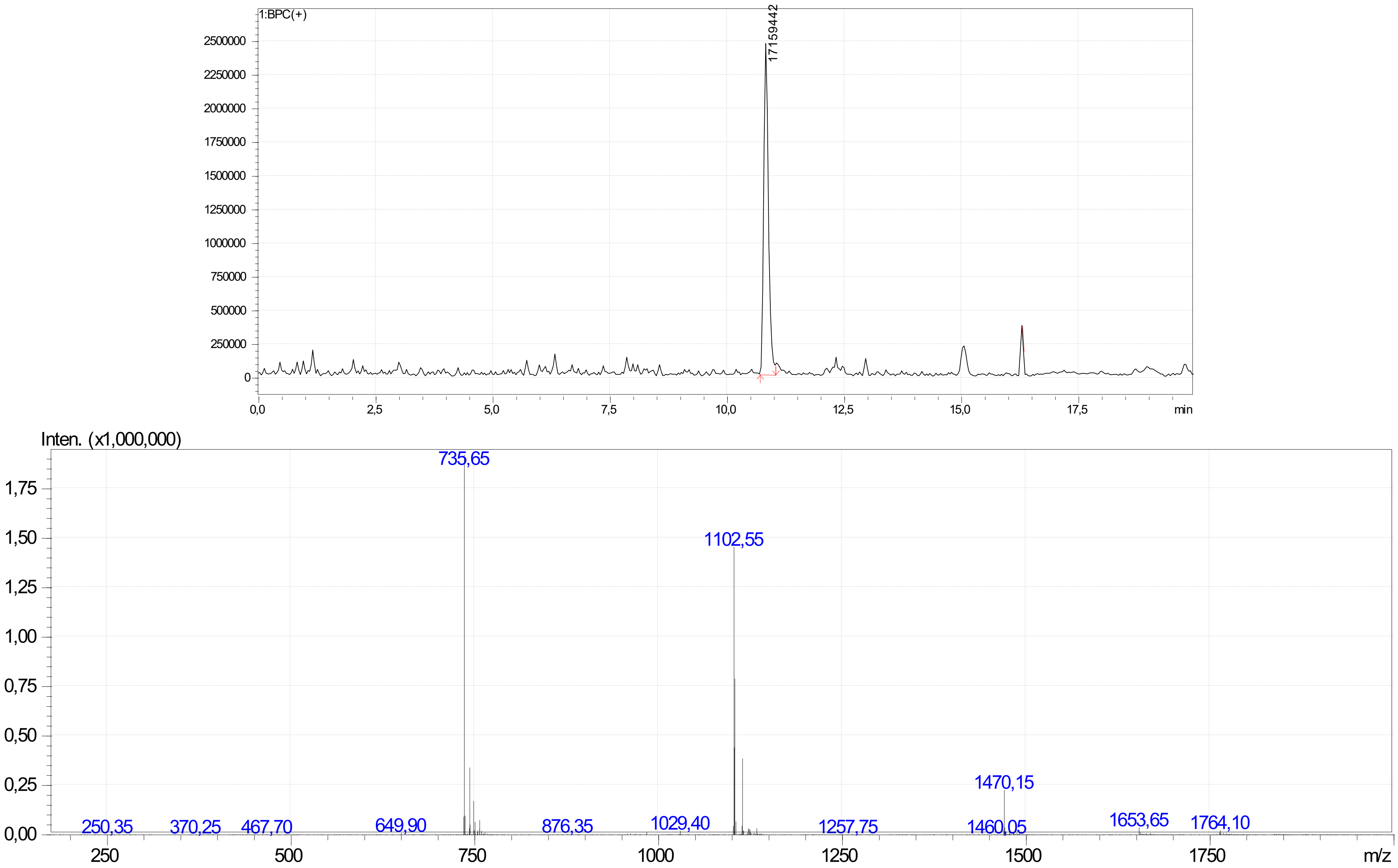

*HPLC-MS (ESI)* ***11***.

Compound **12**

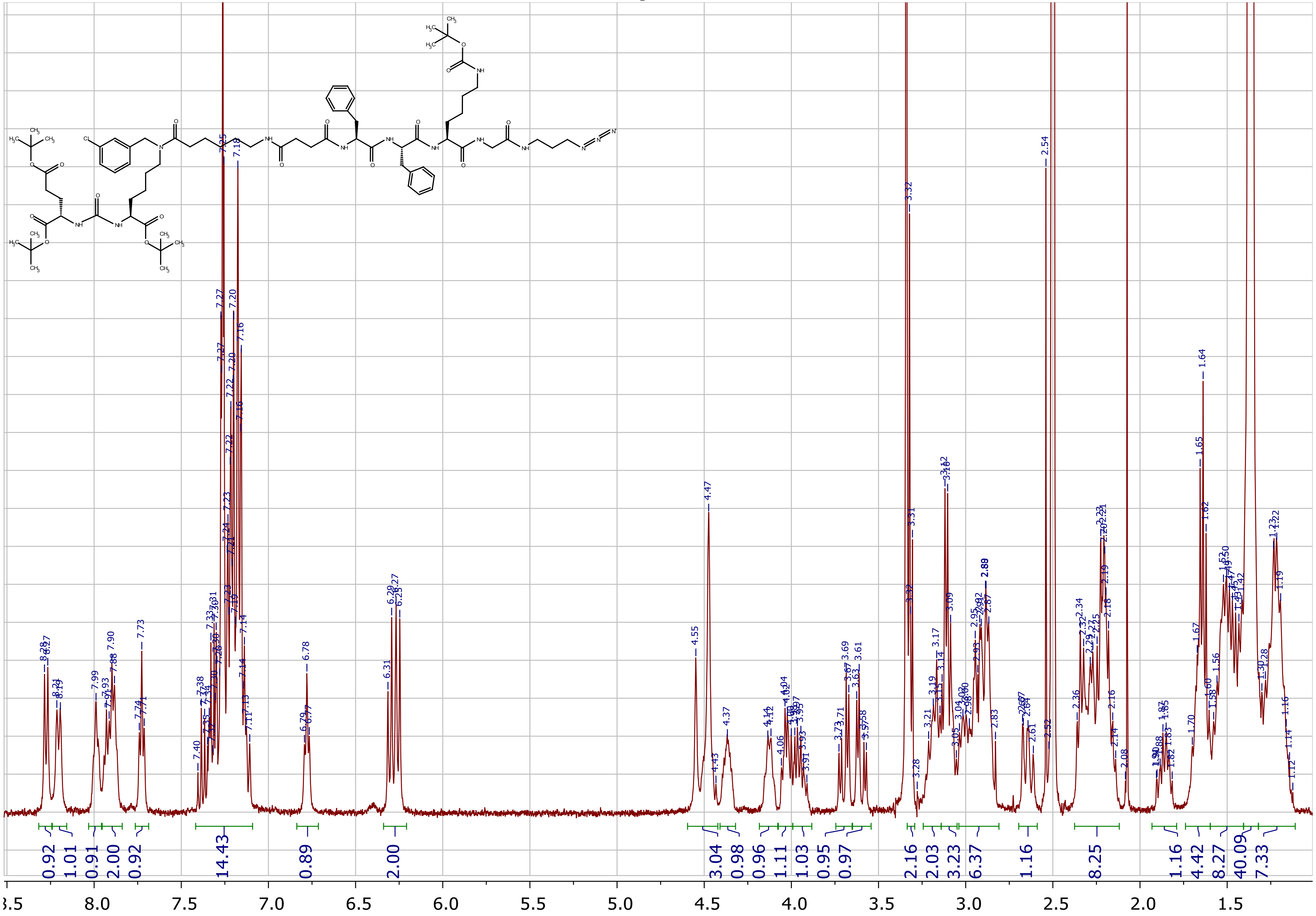

$^1H$ NMR **12**, 400 MHz, 293° K in DMSO-$d_6$

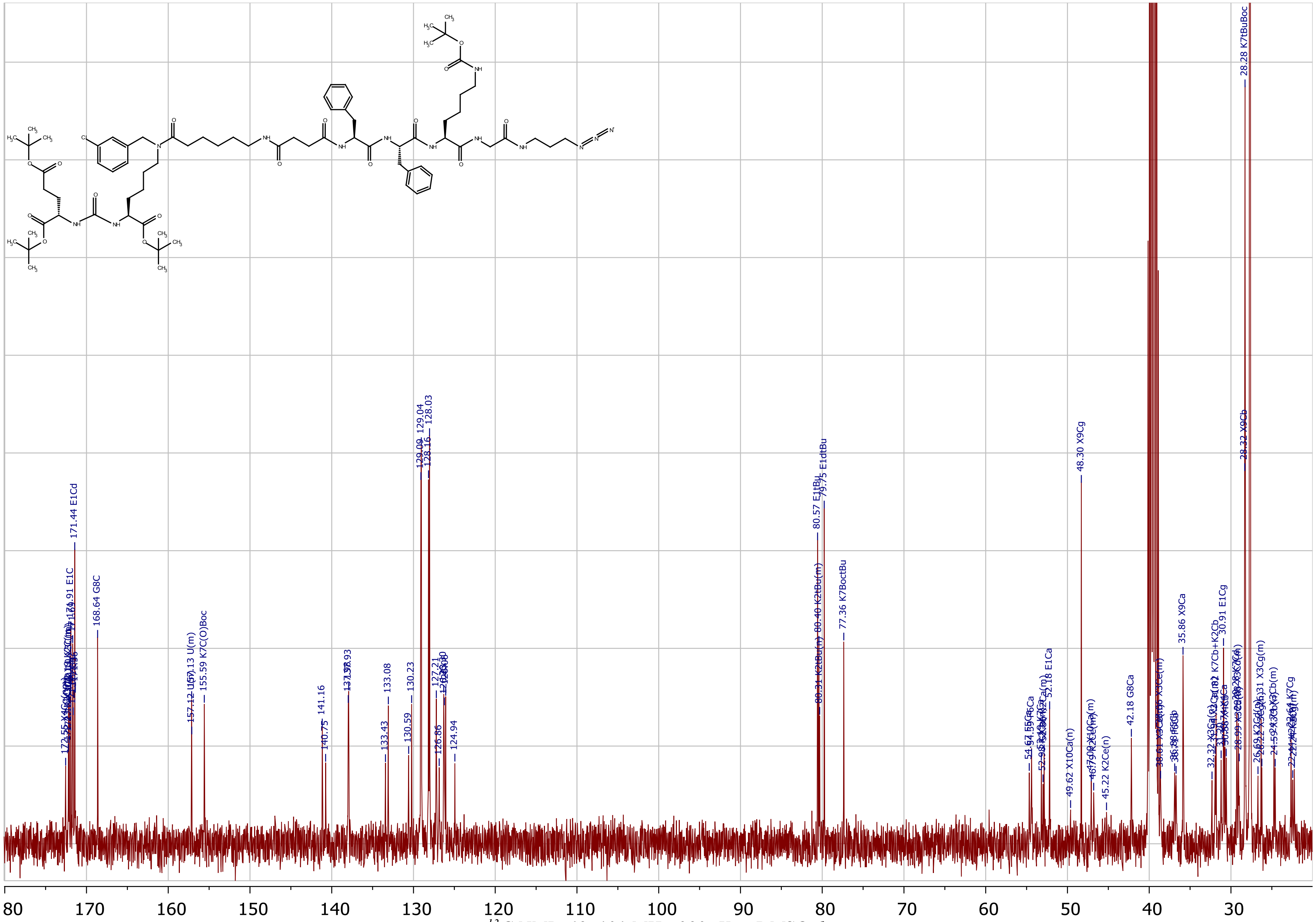

$^{13}$C NMR **12**, 101 MHz, 293° K in DMSO-$d_6$

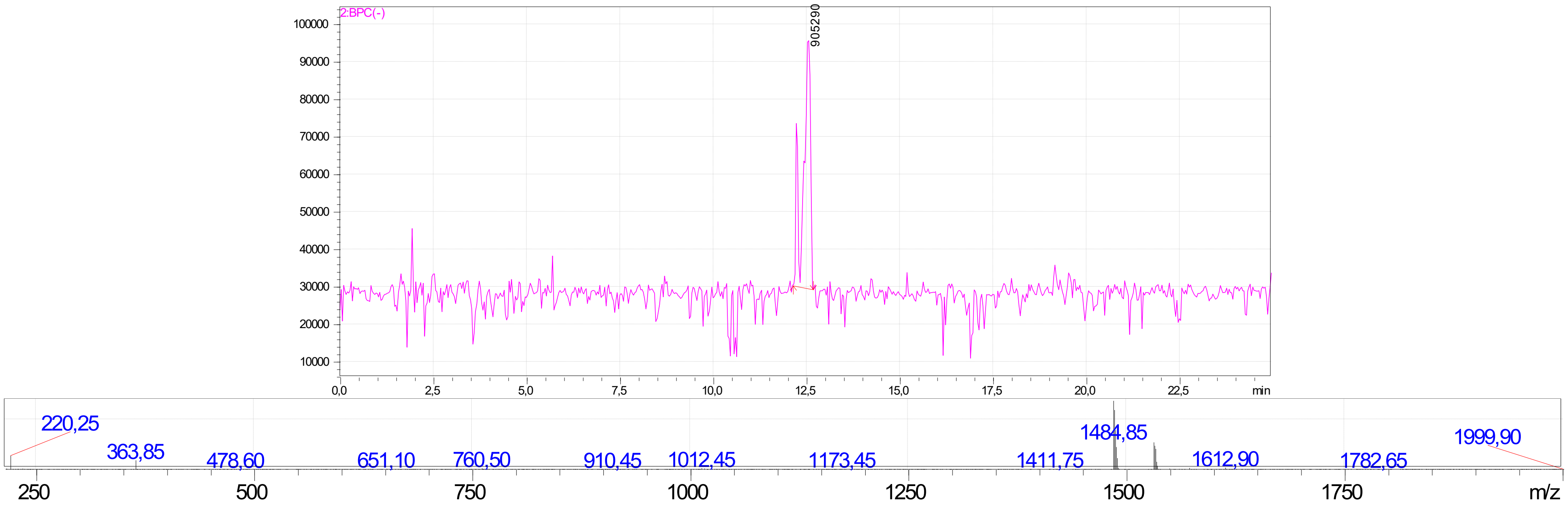


*HPLC-MS (ESI)* ***12***.

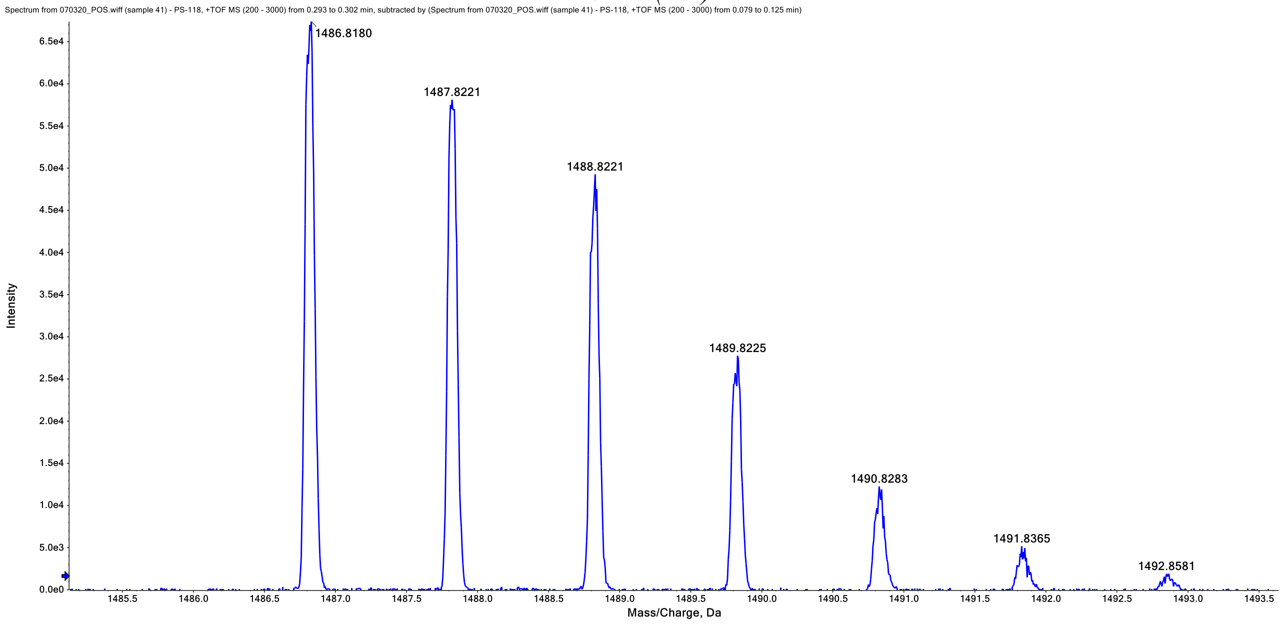


*HRMS* ***12***.

Compound **13**

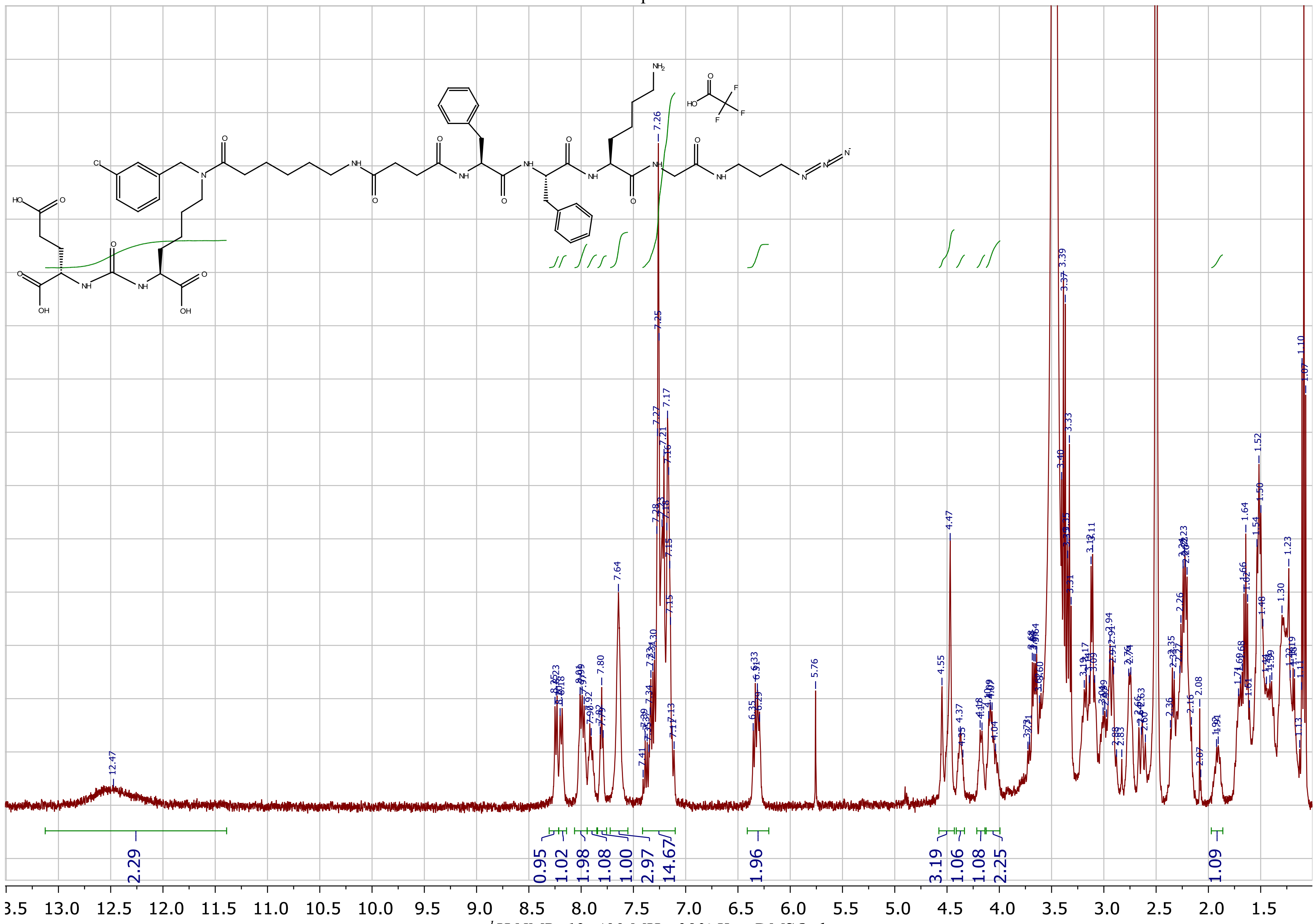

$^{1}$H NMR **13**, 400 MHz, 293° K in DMSO-$d_6$

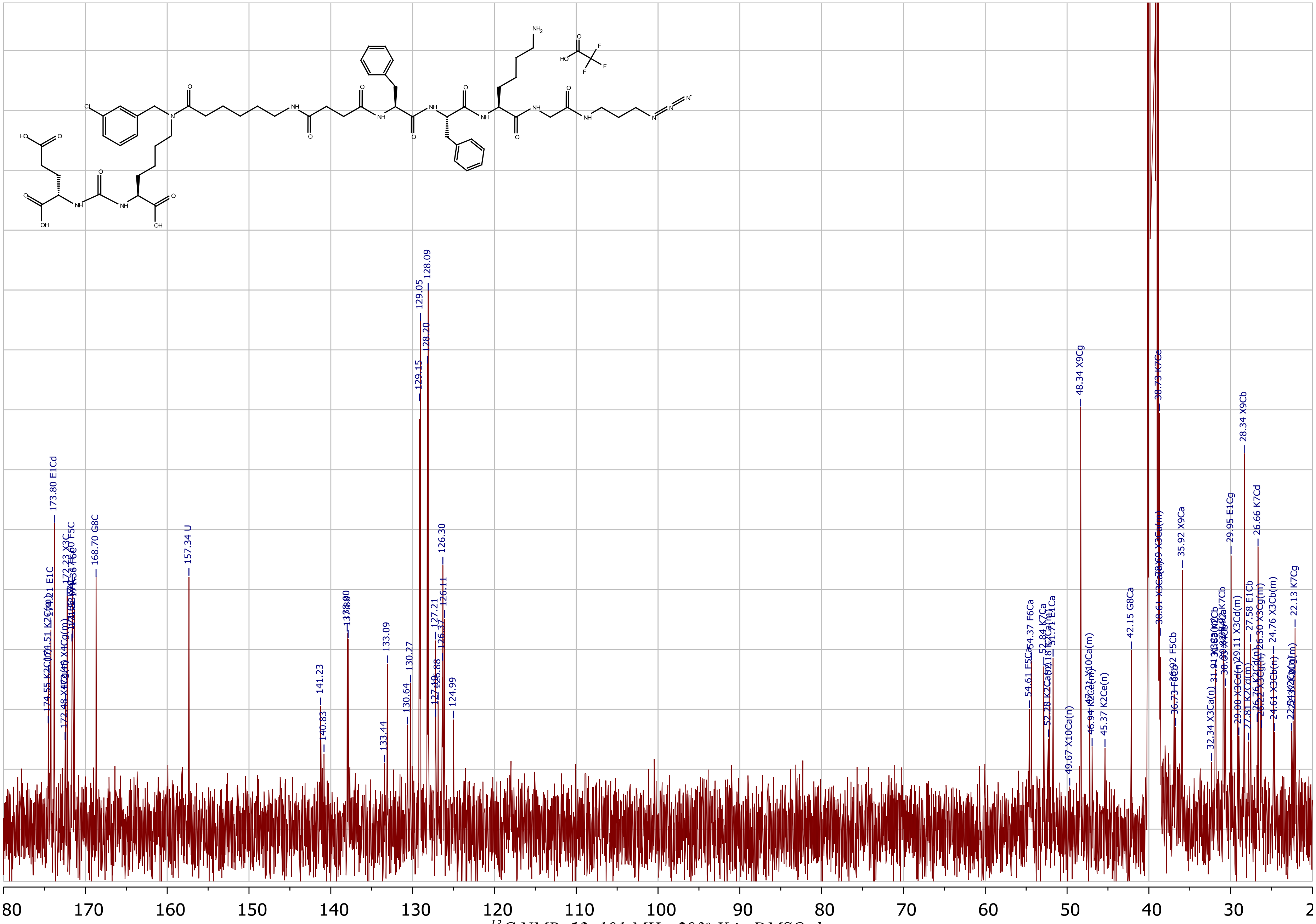


$^{13}C$ NMR **13**, 101 MHz, 293º K in DMSO-$d_6$

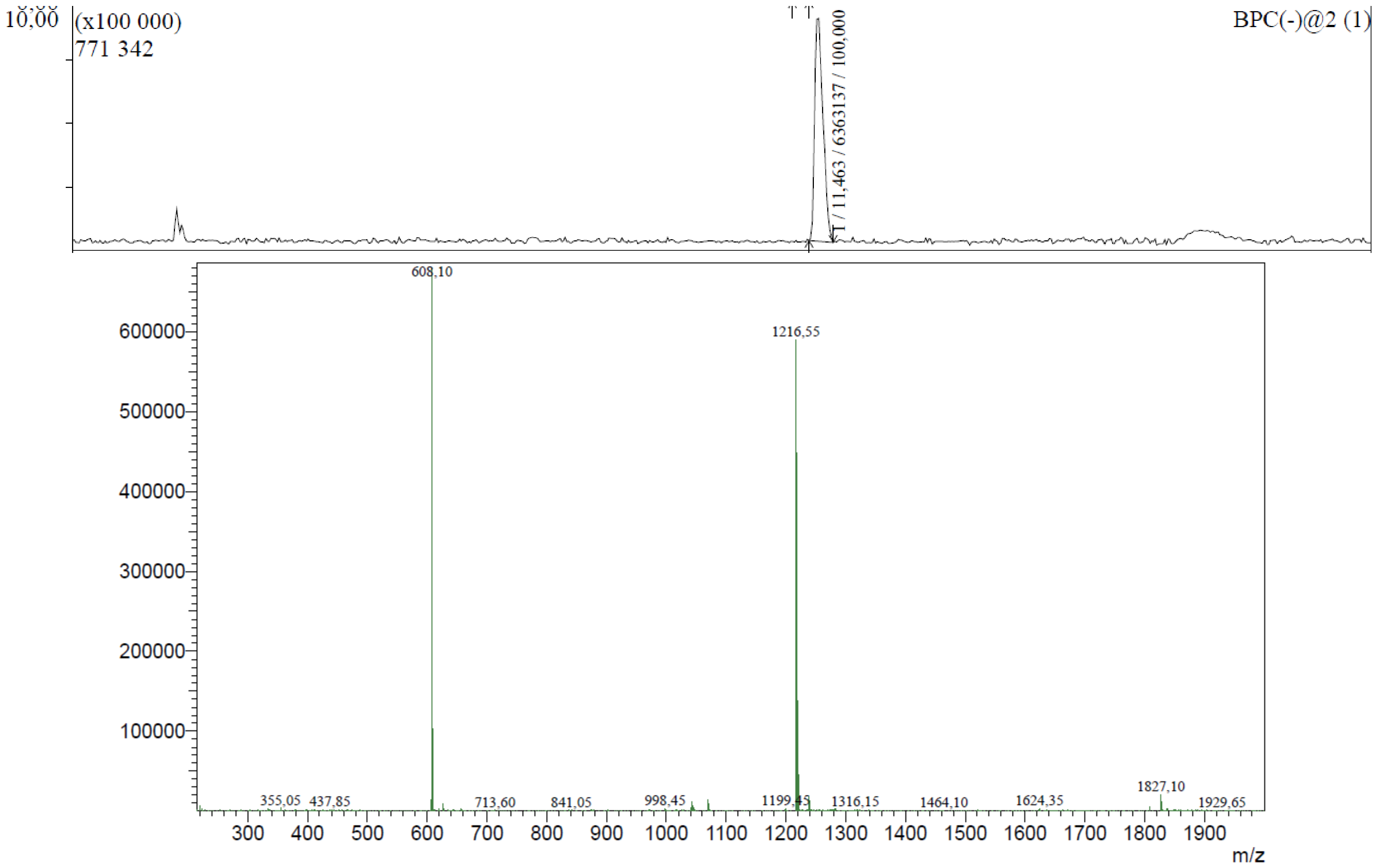


*HPLC-MS (ESI)* ***13***.

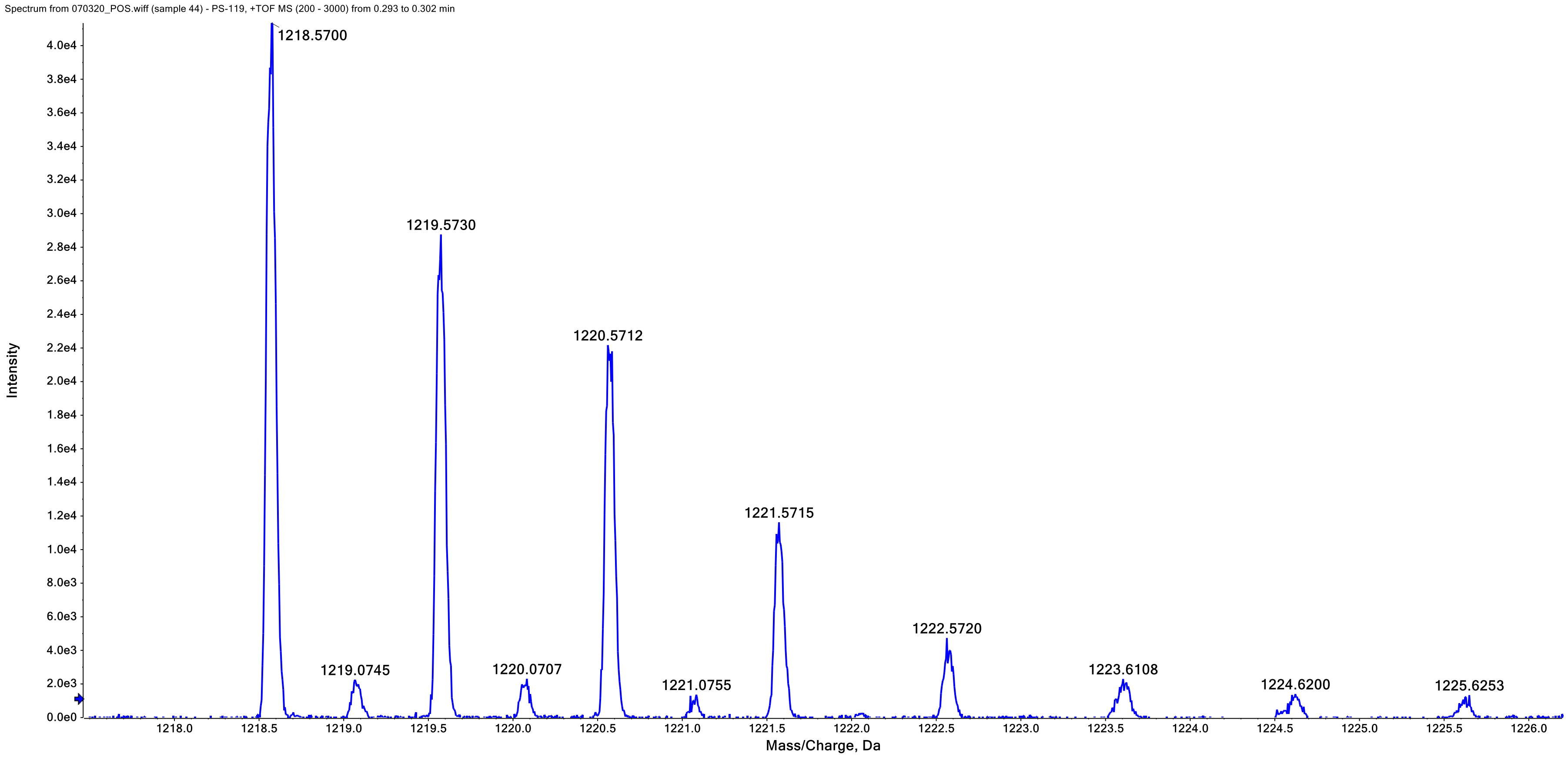

Spectrum from 070320_POS.wiff (sample 44) - PS-119, +TOF MS (200 - 3000) from 0.293 to 0.302 min
Intensity
Mass/Charge, Da
1218.5700
1219.5730
1220.5712
1221.5715
1222.5720
1219.0745
1220.0707
1221.0755
1223.6108
1224.6200
1225.6253


*HRMS **13**.*

Compound **14**

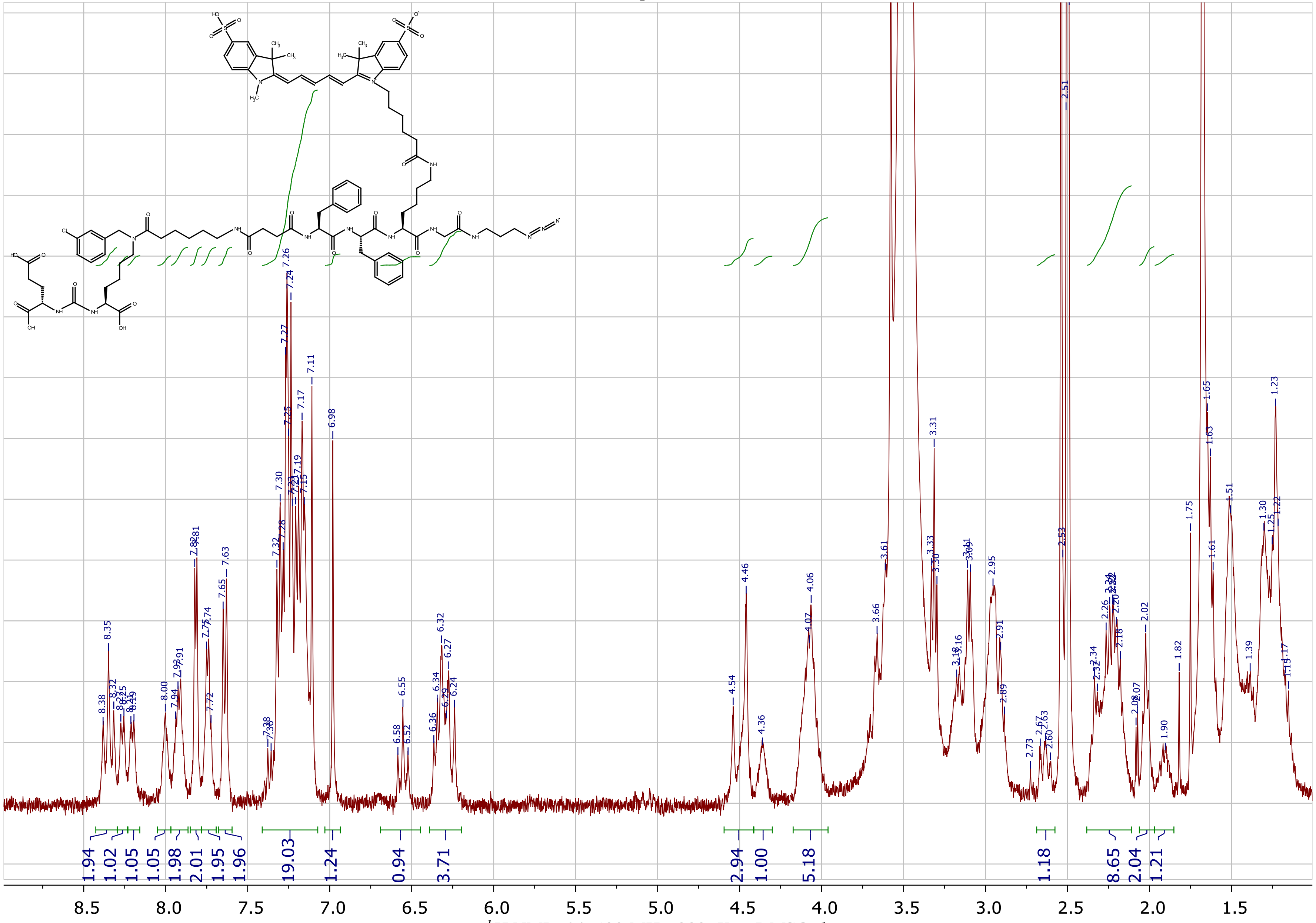


$^{1}H$ NMR **14**, 400 MHz, 293° K in DMSO-$d_6$

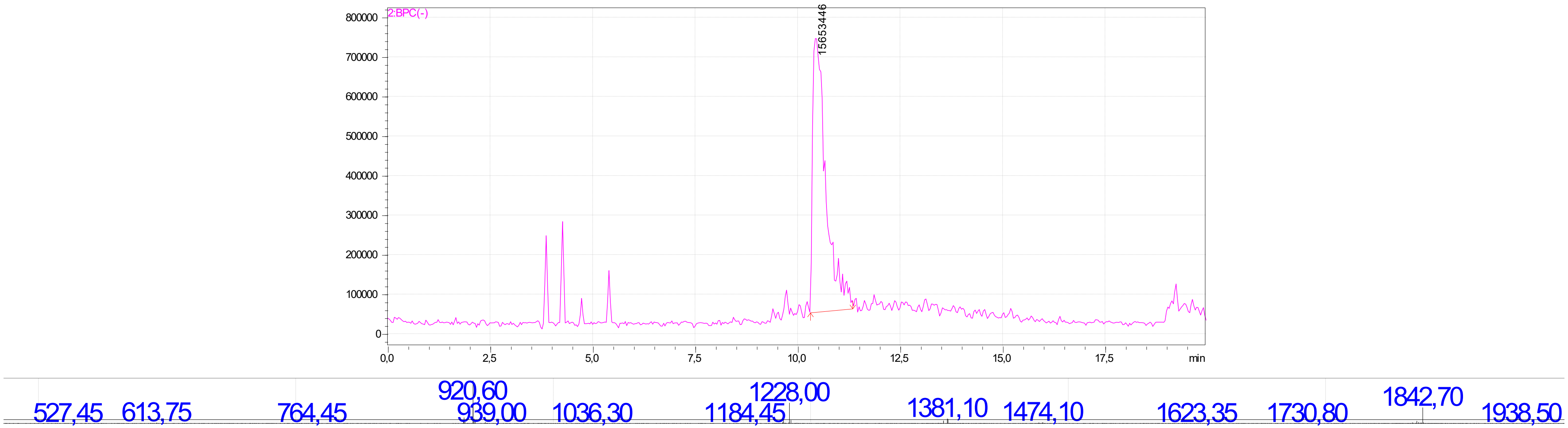


*HPLC-MS (ESI)* ***14***.

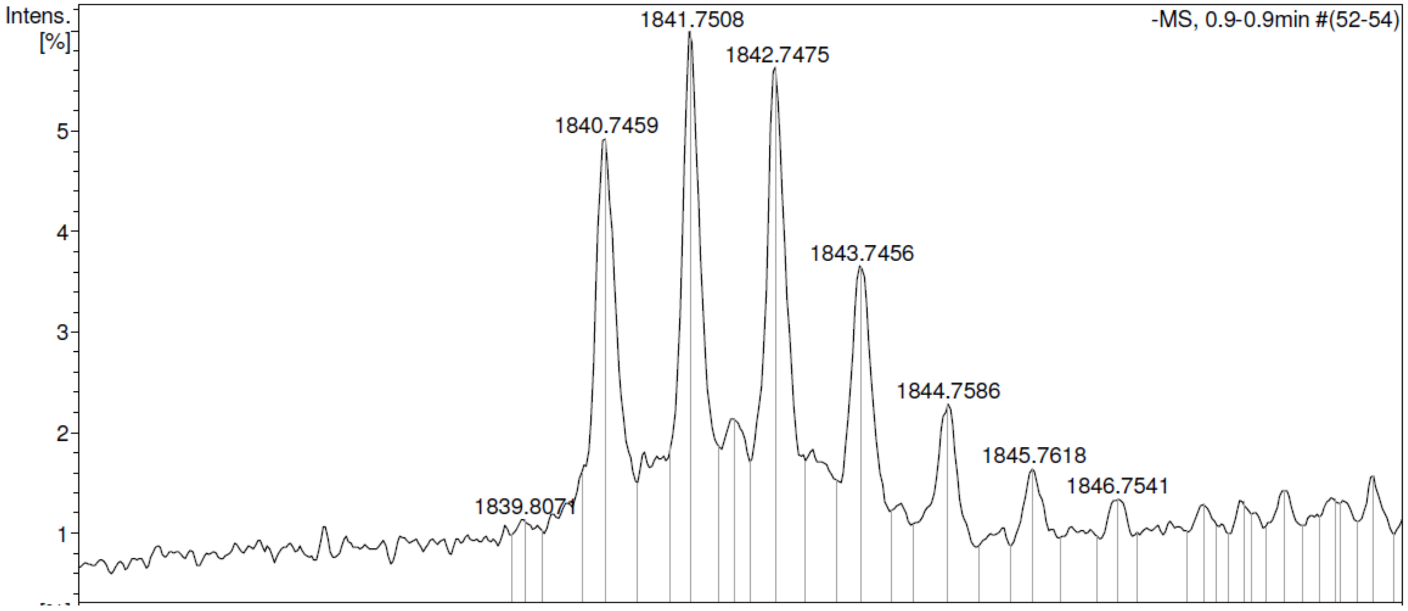


*HRMS* ***14***.

***Flow cytometry***

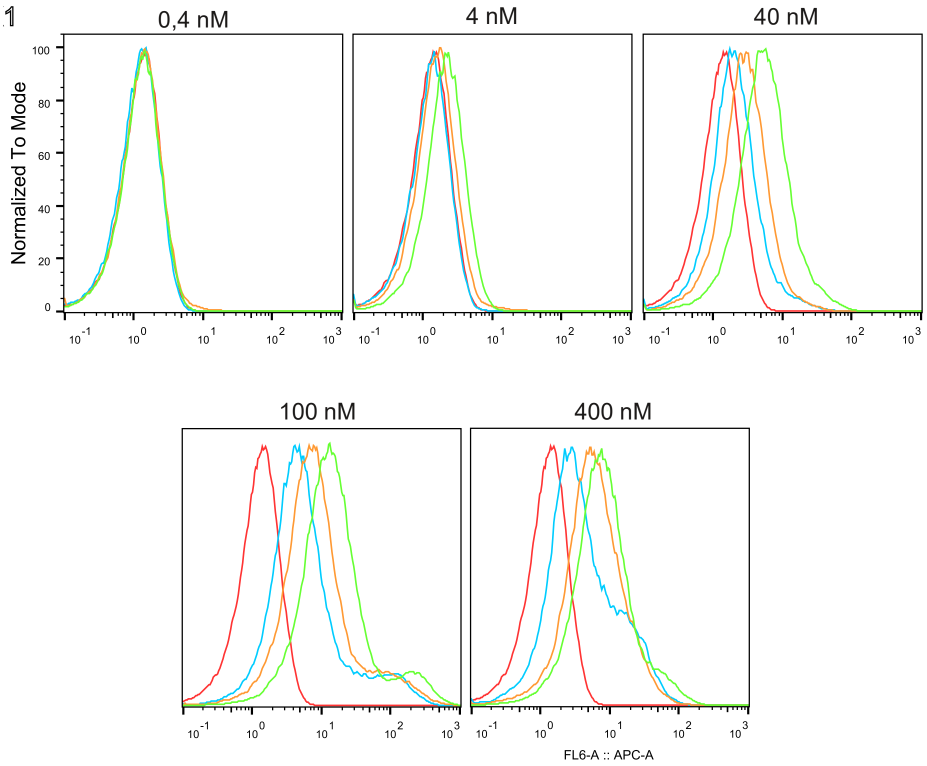


**Figure. S1.** The compounds **11** and **14** stain PSMA-expressing LNCaP cells in a dose-dependent manner, reaching plateau around 100 nM. Histograms of fluorescent signal distribution across the range of compound concentrations (0.4 nM, 4 nM, 40 nM, 100 nM, 400 nM).

***In vivo* biodistribution analysis**

*Normal mice biodistribution*

**Table S1.** Normal biodistribution of compounds [$^{68}$Ga]Ga-**10** and **[$^{68}$Ga]Ga-**11 in mouse models.

| | **ID/g, %** | | | | | | | |
|---|---|---|---|---|---|---|---|---|
| | **30 min** | | **60 min** | | **90 min** | | **120 min** | |
| | **10** PS-204 (m-Cl-PhePhe-Lys-Gly-дота) | **11** PS-205 (m-Cl-PhePhe-Lys(флуор)-Gly-дота) | **10** | **11** | **10** | **11** | **10** | **11** |
| **Blood** | **0.52±0.11** | **0.42±0.14** | **0.3±0.1** | **0.26±0.03** | **0.10±0.02** | **0.33±0.05** | **0.08±0.02** | **0.40±0.03** |
| Salivary glands | 0.15±0.07 | 0.10±0.01 | 0.09±0.03 | 0.07±0.02 | 0.05±0.02 | 0.07±0.02 | 0.07±0.02 | 0.06±0.01 |
| Heart | 0.29±0.11 | 0.22±0.11 | 0.17±0.09 | 0.14±0.03 | 0.03±0.02 | 0.15±0.02 | 0.05±0.01 | 0.19±0.04 |
| Lungs | 0.34±0.11 | 0.20±0.07 | 0.19±0.05 | 0.14±0.02 | 0.06±0.03 | 0.15±0.04 | 0.07±0.01 | 0.18±0.02 |
| Stomach | 0.09±0.06 | 0.06±0.02 | 0.10±0.02 | 0.04±0.02 | 0.01±0.01 | 0.03±0.01 | 0.04±0.01 | 0.07±0.01 |
| Intestine | 0.05±0.01 | 0.04±0.01 | 0.06±0.02 | 0.03±0.01 | 0.02±0.02 | 0.03±0.01 | 0.03±0.01 | 0.05±0.01 |
| Liver | 0.10±0.02 | 0.09±0.04 | 0.08±0.02 | 0.06±0.01 | 0.03±0.02 | 0.08±0.03 | 0.05±0.01 | 0.09±0.01 |
| **Kidneys** | **7.17±1.52** | **2.36±0.74** | **8.42±2.62** | **2.12±0.21** | **6.90±0.83** | **4.04±0.96** | **7.47±0.56** | **4.68±0.3** |
| Spleen | 0.12±0.04 | 0.07±0.04 | 0.08±0.02 | 0.04±0.02 | 0.02±0.01 | 0.06±0.02 | 0.05±0.01 | 0.06±0.02 |
| Prostate | 0.20±0.09 | 0.05±0.02 | 0.06±0.01 | 0.04±0.02 | 0.06±0.02 | 0.05±0.02 | 0.05±0.01 | 0.04±0.02 |
| Muscles | 0.09±0.03 | 0.04±0.03 | 0.06±0.01 | 0.04±0.01 | 0.02±0.01 | 0.06±0.02 | 0.04±0.03 | 0.05±0.01 |

*Xenograft mice model biodistribution*

**Table S2.** – Distribution (% of administered activity per 1 g of tissue) of the radiolabeled conjugate [$^{68}$Ga]Ga-**11** in the bodies of Nu line mice with LNCaP line tumor xenografts.

| Organ/tissue | Time after [$^{68}$Ga]Ga-**11** administration, min | | |
|---|---|---|---|
| | **30** | **60** | **90** |
| **Blood** | 7,30±0,38 | 4,41±0,71 | 3,12±0,12 |
| Salivary glands | 2,00±0,47 | 1,41±0,12 | 1,74±0,33 |
| Heart | 3,04±0,27 | 2,41±0,37 | 1,51±0,03 |
| Lungs | 4,96±0,68 | 3,13±0,74 | 2,75±0,08 |
| Stomach | 5,07±2,74 | 1,81±0,15 | 0,92±0,22 |
| Intestine | 1,30±0,12 | 1,10±0,19 | 0,83±0,09 |
| Liver | 2,29±0,27 | 1,70±0,24 | 1,38±0,19 |
| Kidneys | 40,39±4,22 | 89,93±27,44 | 74,45±14,35 |
| Spleen | 2,00±0,31 | 1,50±0,48 | 1,27±0,18 |
| Prostate | 1,41±0,35 | 1,20±0,58 | 1,27±0,51 |
| Reproductive system | 1,57±0,59 | 1,42±0,29 | 1,73±0,57 |
| Muscles | 0,57±0,14 | 0,48±0,11 | 0,54±0,03 |
| Tibia | 1,13±0,40 | 1,49±0,38 | 1,44±0,18 |
| Tumor | 6,76±1,14 | 10,66±2,98 | 14,06±1,04 |
| Bladder | 1,56±0,32 | 2,94±0,30 | 2,57±0,99 |

**Table S3.** Biodistribution for [$^{68}$Ga]Ga-**PSMA-11** and [$^{68}$Ga]Ga-P**SMA-617** (60 min after drug administration) evaluated on LNCaP xenograft model.

| | **PSMA-617** | **PSMA-11** |
|---|---|---|
| Blood | 0.62±0.11 | 0.25±0.15 |
| Lungs | 0.51±0.11 | 0.32±0.15 |
| Heart | 0.26±0.06 | 0.24±0.07 |
| Salivary glands | 0.59±0.52 | 1.40±0.62 |
| Stomach | 0.25±0.05 | 0.52±0.27 |
| Liver | 0.17±0.04 | 0.10±0.04 |
| Kidneys | 13.39±5.85 | 32.05±6.94 |
| Intestine | 0.26±0.03 | 0.22±0.04 |
| Spleen | 0.24±0.16 | 1.06±0.59 |
| Reproductive system | 0.58±0.32 | 0.22±0.15 |
| Prostate | 0.40±0.07 | 0.90±0.27 |
| Bladder | 3.30±2.74 | 2.07±1.3 |
| Tibia | 0.53±0.22 | 0.38±0.12 |
| Muscle | 0.37±0.23 | 0.16±0.03 |
| Tumor | 16.23±9.19 | 5.98±2.11 |
